# Machine Learning Accelerated Computational Design of Bio‐Inspired Catalysts in the Nitrogen Reduction Reaction

**DOI:** 10.1002/adma.73603

**Published:** 2026-06-06

**Authors:** Leonardo Di Ciano, Zihan You, Haoran Chen, Qifan Zhong, Rong‐Zhen Liao, Shaoqi Zhan

**Affiliations:** ^1^ Department of Chemistry‐Ångström Laboratory Molecular Biomimetics Uppsala University Uppsala Sweden; ^2^ School of Metallurgy and Environment Central South University Changsha China; ^3^ School of Chemistry and Chemical Engineering Huazhong University of Science and Technology Wuhan China

**Keywords:** computational workflow, feature engineering, machine learning, nitrogen reduction reaction

## Abstract

The development of efficient catalysts for nitrogen conversion to ammonia is critical for a sustainable alternative to the energy‐intensive Haber–Bosch process. Yet, rational catalyst design remains highly challenging, compounded by complex structure–function relationships within realistic conditions. Herein, we present an integrated computational framework combining quantum chemical calculations with 27 machine learning models to predict experimental catalytic metrics in metal–ligand complexes. The models are trained and validated on a large experimental database and demonstrate high predictive accuracy across multiple tasks. For classification, family 1 and family 2 catalysts achieved test accuracies up to 1. Regression models yield test *R* ^2^ values of 0.91 and 0.88 for turnover frequency (TOF) and turnover number (TON) predictions in family 1, and 0.96 and 0.99 in family 2. Notably, the models accurately capture time‐dependent variability of TOF and TON for new complexes, with predicted values closely matching experimental results. Moreover, strong transfer learning capability is observed for structurally distinct coordination architectures. Feature interpretation reveals clear design principles for optimal catalysts involving metal spin state, ligand geometry, charge distribution, and experimental conditions. Together, this study established an efficient and practical framework for discovery and inverse design of high‐performance catalysts under realistic conditions, with broader relevance to electrocatalysis.

## Graphical abstract

We introduce a computational workflow that combines quantum chemical calculations and machine learning techniques to predict the catalytic performance of a wide range of catalysts in the nitrogen reduction reaction (NRR). The analysis of the trained models provides insights into the complex structure–activity relationship in experimental catalytic conditions and paves the way for the rational design of high‐performance NRR electrocatalysts.

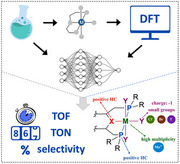

## 1 Introduction

As one of the “holy grails” in chemistry [1, 2, 3], catalyst rational design remains a central and urgent challenge. This challenge is particularly seen in the development of electrocatalysts for small‐molecule activation in sustainable reactions, such as N_2_ fixation to produce NH_3_ [4], and CO_2_ conversion to generate value‐added C_2+_ chemicals [5], where current catalysts have yet to achieve catalytic proficiencies comparable to those of enzymes. The sluggish activity and poor product selectivity stem largely from the complexity of structure–function relationships within catalytic conditions, which hinders catalyst development. In such systems, reaction conditions significantly influence performance, and even subtle changes in factors such as solvents, electrolytes, or applied potentials can have marked consequences on catalytic activity, selectivity, and stability. For instance, strategies that restrict proton or electron transfer to suppress the competition of the hydrogen evolution reaction (HER) in the nitrogen reduction reaction (NRR) also reduce NH_3_ formation rates [6]. This perspective underscores the need to develop approaches accounting for complex catalytic conditions for efficiently evaluating catalyst performance, thereby enabling the truly rational design of catalytic systems to achieve targeted catalytic functionalities.

Computational approaches have advanced significantly over the years, shifting from providing mechanistic insights to incorporating cutting‐edge machine learning (ML) techniques as effective “shortcuts” for the rational design of novel catalysts. Classical computational methods, such as density functional theory (DFT), can provide qualitative structure–property relationships to accurately predict catalytic behaviors at a reasonable computational cost [7, 8]. ML using string‐ and topological structure‐based encodings, including molecular fingerprints, etc. [9, 10] is powerful for the quantitative discovery of catalysts and the prediction of catalytic performance by harnessing data interrelationships [11]. However, they cannot readily trace the physical origins of catalytic performance. Combining quantum chemical calculations with ML algorithms can utilize widely existing data and associated chemical principles to develop predictive models that are generalizable toward complex systems [12]. For instance, only one recent study has combined quantum chemistry and ML to investigate a small set of [Fe(CAAC)_2_] (CAAC = cyclic (alkyl)(amino)carbene) NRR catalysts, primarily exploring metal centers and combinations of CAAC ligands [13]. For the design of molecular NRR catalysts under realistic reaction conditions, such integrated approaches remain largely unexplored, despite the critical importance of the NRR process.

The NRR to produce NH_3_ is imperative as ammonia is an essential feedstock for fertilizers and industrial chemicals, and a potential carbon‐free fuel alternative [14]. The challenge of N_2_ fixation lies in the chemically inert N_2_ molecule, which has a strong homonuclear triple bond (941 kJ mol^−1^), and the competition with the HER [15]. The industrial Haber–Bosch process to produce NH_3_ operates under harsh conditions (400–550°C and 15–25 MPa). This process consumes ∼2% of the global annual energy output and accounts for ∼1.3% of global carbon dioxide emissions. In contrast, biological N_2_ fixation by nitrogenases, which features FeM‐cofactors (*M* = Mo, V, and Fe), occurs under mild conditions (<40°C, atmospheric pressure) with an optimal turnover frequency (TOF) of ∼1 s^−1^ [2]. This has inspired scientists to develop biomimetic catalysts to promote NRR under ambient conditions. Transition‐metal complexes with abundant d orbital electrons and unoccupied orbitals are promising for activating strong N≡N triple bonds [16]. These homogeneous biomimetic complexes have advantages, including well‐defined active sites and coordinated ligands (Table 1) [17] that can determine their electronic and steric properties, modulating catalyst–substrate interaction under given external conditions (solvent, pH, electrolyte). These features facilitate mechanistic insights and, ideally, the identification of key chemical factors that can be optimized to enhance catalytic efficiency.

**TABLE 1:** Categories, topologies and coordination spheres (in red) of reported catalysts compiled for the computational workflow.

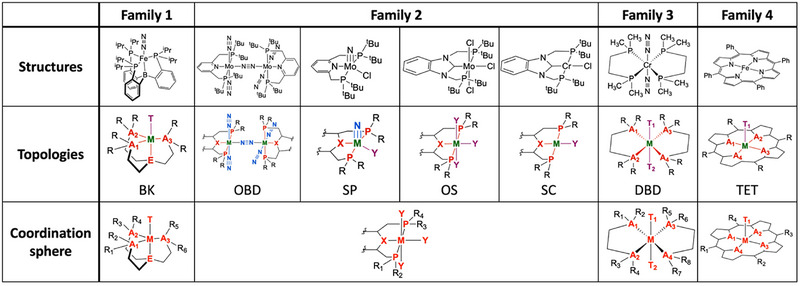

Various NRR catalysts (Figure S1) with distinct ligand motifs have been reported. Among these, tripodal scaffold–based metal complexes have been prominent, exemplified by the family of monometallic molybdenum complexes reported by Schrock's group, such as (HIPTN_3_N)Mo(N_2_) derivatives (HIPT = hexa‐iso‐propyl‐terphenyl) [18, 19]. Similarly, Peters’ group developed bio‐inspired P_3_^B^Fe catalysts and their derivatives with a single Fe atom coordinated by a tripodal P_3_^X^ ligand framework (P_3_^B^ = (*o*‐*^i^* Pr_2_PC_6_H_4_)_3_B) [20, 21, 22, 23, 24, 25]. In addition, Nishibayashi's group reported PNP‐ and PCP‐based dimeric and monomeric molybdenum catalysts (PNP = 2,6‐bis(di‐tert‐butylphosphinomethyl)pyridine, PCP = 1,3‐bis((di‐tert‐butylphosphino)methyl) benzimidazol‐2‐ylidene), including [Mo(III)Br_3_(PNP)] and [Mo(III)Br_3_(PCP)] [26, 27, 28, 29, 30, 31]. These systems achieved turnover numbers (TONs) of up to 1800 and 9000, respectively, as well as related derivatives supported by PPP‐pincer ligands [32]. Additional examples include catalysts featuring structurally unconventional two‐coordinate [Fe(CAAC)_2_] complexes [33] and catalysts with planar coordination environments [34, 35]. Despite these progresses, the accelerated rational design of molecular NRR catalysts under realistic reaction conditions remains limited. To the best of our knowledge, there is currently no efficient approach that enables the systematic prediction of homogeneous, bio‐inspired NRR catalysts, integrating novel catalyst design with reliable evaluation of catalytic performance.

**FIGURE 1:**
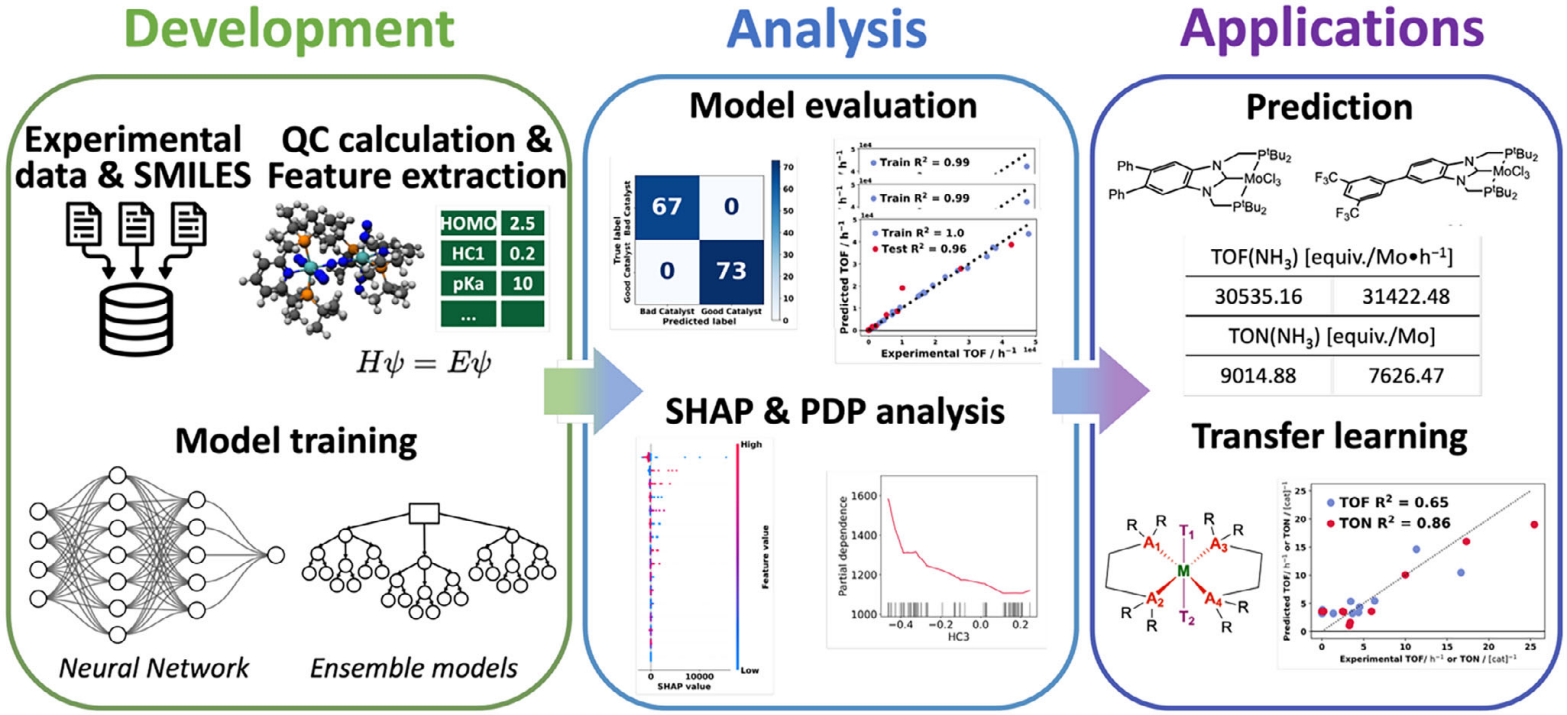
Overview of ML‐integrated computational workflow for molecular NRR catalyst design.

In this work, we developed a streamlined computational workflow integrated with high‐precision ML models to accelerate the design of highly active NRR catalysts under experimental reaction conditions. Our models incorporate experimental condition variables, atomic and molecular structural features, and catalytic performance metrics, that is, TOF, TON, and selectivity ratios, using ∼524 catalytic datasets in training and testing. Both classifier and regression models achieved high test *R* ^2^ scores in predicting experimental catalytic performance metrics. More importantly, the trained models are further applied to predict catalytic metrics of new, structurally distinct complexes, demonstrating strong agreement with recently reported experimental results. Feature importance analysis revealed key characteristics of effective NRR catalytic systems under realistic conditions, aiding mechanistic insights and future inverse design of new catalysts with targeted catalytic performance. In addition, our models exhibit strong transfer‐learning capability, which can be applied to a broad range of homogeneous NRR complexes. Finally, we developed a simplified molecular input line entry system (SMILES)‐based prediction pipeline that enables rapid performance estimation of new unknown catalysts using our well‐trained, interpretable model and can be readily coupled with open‐access databases. Overall, this work provides an efficient and general computational framework for target‐driven design and performance prediction of NRR catalysts under realistic reaction conditions. It can be extended to large‐scale computational screening using open‐access datasets of organic ligands or metal–ligand complexes and integrated with automated experimentation in a closed‐loop experiment–model workflow (Figure 1).

## 2 Results and Discussion

### 2.1 Data Analysis

A database of approximately 524 experimentally reported results was compiled, with each comprising experimental reaction conditions and the NH_3_ production efficiency (Table S1). It corresponds to a total of 218 catalysts, mainly developed by the Peters and Nishibayashi's groups (Figure S1). To formulate an efficient workflow, these catalysts are categorized into four families based on their geometric structures, each characterized by a well‐defined structural motif (Table 1). This framework offers a tool for future ligand modification, as long as the binding atom motif is preserved. Family 1 comprises 103 compiled experimental entries [18, 19, 20, 21, 22, 23, 24, 25, 36, 37, 38, 39, 40, 41, 42, 43], representing 36 catalyst structures. Family 2 contains 391 experimental entries [26, 27, 28, 29, 30, 31, 32, 44, 45, 46, 47, 48, 49, 50, 51, 52, 53, 54, 55, 56, 57, 58, 59, 60, 61, 62, 63, 64, 65, 66], corresponding to 167 catalyst structures. Families 3 and 4 together account for 30 experimental entries [33, 34, 35] and 15 catalyst structures. The most frequently applied metal is Mo (46.89%), followed by Fe (28.09%) (Figure S2). This metal distribution in the catalysts is rather unsurprising, given that Mo, Fe, and V are the metals naturally found in the active sites of nitrogenases [67]. While the datasets for families 1 and 2 are sufficient for training dedicated ML models, this is not the case for families 3 and 4. In particular, family 4 often involves porphyrin systems integrated into metal–organic frameworks, which would require a different evaluation approach. However, the topology of family 4 is included in the computational workflow for future research studies.

### 2.2 Quantum Chemical Calculations

The validated workflow of quantum chemical calculations was subsequently applied to compute chemical properties of the compiled catalytic systems, including acid p*K*_a_ values and reductant redox potentials in their relevant solvents, to construct a quantum chemical dataset. Catalyst chemical properties explicitly sample the first coordination sphere of the metal, defined as the six atoms closest to the metal center (Table 1), for instance, through population analysis. The effect of more distant groups is instead implicitly captured via molecular‐level properties, such as the highest occupied molecular orbital–lowest unoccupied molecular orbital (HOMO–LUMO) energy gap and dipole and quadrupole moments. For the population analysis features, values are extracted only for the metal (indicated as 0) and the six closest atoms, indexed from 1 to 6 according to their distance from the metal center. Overall, descriptors extracted from the lowest energy spin state calculation comprise geometry properties, non‐covalent interactions, steric factors, electronic and atomic level properties, as well as molecular level properties (Table S5). Predicting catalytic metrics directly from a few chemical properties is a challenge, likely because catalytic conditions are complex and affect the results. This is true from our preliminary analysis in exploring potential relationships between HOMO–LUMO gaps versus the maximum experimental TOF (Figures S10–S12), where we didn't identify clear trends. Therefore, more advanced ML methods are needed to uncover the governing factors behind catalytic performance.

### 2.3 Property Refinement

**FIGURE 2:**
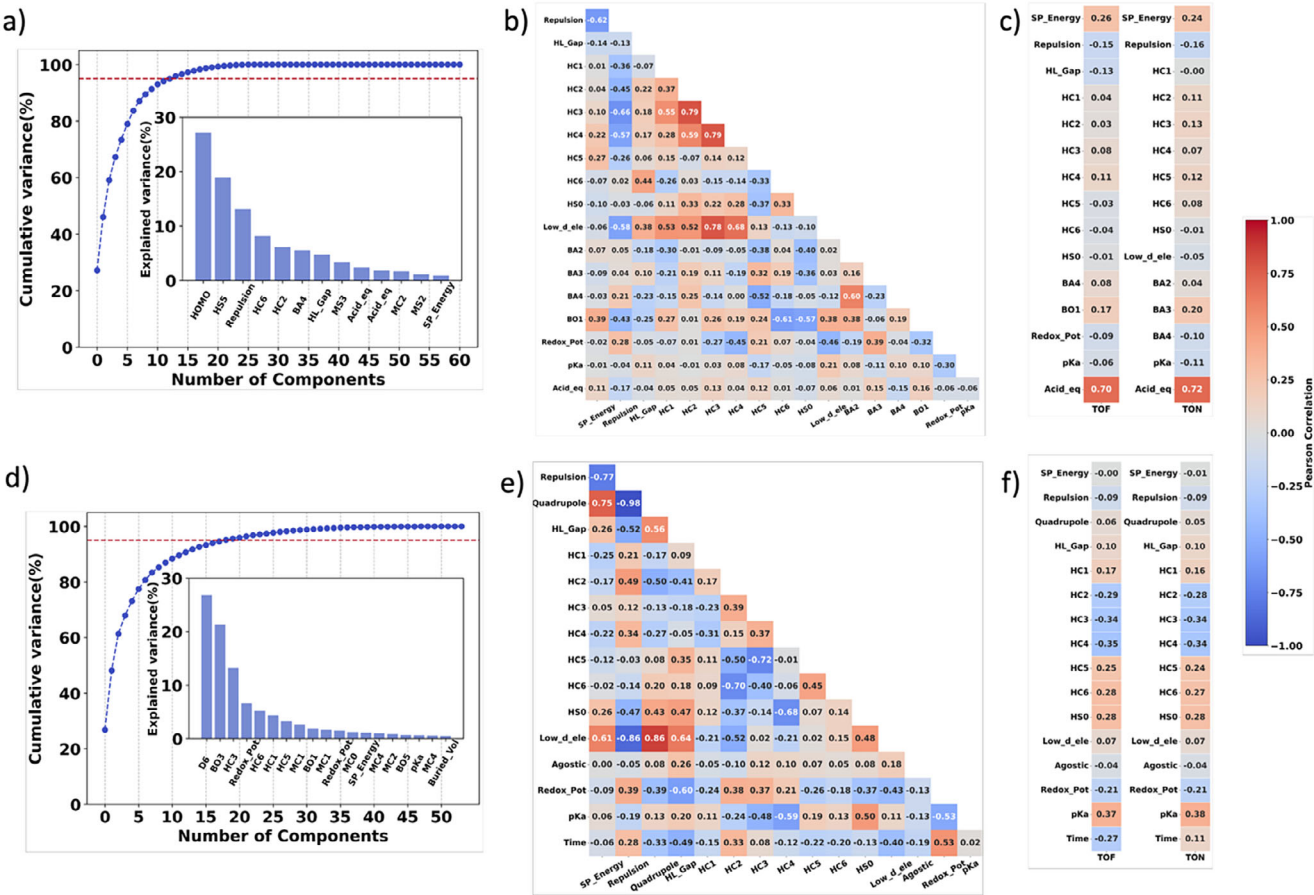
Property analysis of family 1 catalysts: (a) PCA analysis. (b) Pearson correlation coefficient matrix between the selected features. (c) Pearson correlation coefficient matrix between the selected features and the catalytic targets. Property analysis of family 2 catalysts: (d) PCA analysis. (e) Pearson correlation coefficient matrix between the selected features. (f) Pearson correlation coefficient matrix between the selected features and the catalytic targets.

With a total of 62 features from quantum chemical calculations and catalytic performance indicators for each catalytic system (Table S5), we applied multiple techniques to identify the most impactful feature and combined their insights with a heuristic approach to finalize the feature subset for each task. We first applied principal component analysis (PCA) to reduce the dimensionality of the prediction problem. For family 1, PCA identified 13 principal components required to describe 95% of the dataset variance (Figure 2a), while 19 principal components were required for family 2 (Figure 2d). These components are linear combinations of the original features with weights optimized to retain maximum variance. To identify the most influential feature set, we extracted the dominant features from each principal component. For family 1, the most important features in the top three principal components are HOMO (27.2% of explained variance), Hirshfeld spin population HS5 (18.9%), and repulsion energy (13.1%), with acid equivalents identified as a major experimental feature in three principal components. For family 2, the top three components are bond distance D6 (26.8%), bond order BO3 (21.3%), and Hirshfeld charge HC3 (13.2%). Unlike family 1, reductant redox potential and acid p*K*_a_ were major influential experimental features for family 2.

This analysis was complemented by Pearson and Spearman rank correlation matrices, reported for the whole datasets in Figures S14 and S21, to remove redundant variables and retain only those contributing unique information. Strong correlations were observed, for instance, in family 1, among bond order features (BO3–BO6) and between bond distances (D1–D6) and the corresponding Hirshfeld charges (HC1–HC6), as well as the bond distance terms. Similarly, in family 2, strong correlations were identified between Mulliken and Hirshfeld population analysis terms, as well as between them and the bond distance features (D1–D6).

However, these screening techniques are purely numerically driven and can also identify unphysical correlations. To ensure meaningful feature selection for catalyst design, we combined insights from PCA, correlation analysis and chemical reasoning to heuristically conceive the feature subset. This approach led to distinct feature subsets for each family and task, which also account for intrinsic differences in structure and reaction mechanisms across catalyst classes. For instance, in family 1, we included bond angle terms (BA2–BA4) to identify ligand coordination above the metal atom and, together with Hirshfeld charges (HC1–HC6), characterize the local metal environment. Acid equivalents were also retained as an experimental variable highlighted by PCA. On the other hand, for family 2, based on correlation patterns and distinct topology, we included Hirshfeld charges (HC1–HC6) and quadrupole moments. In addition, SP energies were retained to swiftly distinguish between monomeric and dimeric complexes, and the Löwdin d‐electron count and the metal Hirshfeld spin population value (HS0) were included to identify metals and their oxidation states.

Based on these consideration and the heuristic optimization procedure, we selected a subset of 18 features for family 1 (Table S5): single point (SP) energy, repulsion, HOMO–LUMO gap, HC1, HC2, HC3, HC4, HC5, HC6, HS0, Löwdin d electrons number, BA2, BA3, BA4, BO1, reductant redox potential, acid p*K*_a_, acid equivalents (Figure 2b,c). For downstream ML workflows, 15 features were used for classifiers and TOF regression, while three features (HOMO–LUMO gap, reductant redox potential, BO1) have been replaced by Löwdin d‐electron number, BA2, and BA3 in TON regression. For family 2, we selected a subset of 16 features (Table S5): SP energy, repulsion, quadrupole, HOMO–LUMO gap, HC1, HC2, HC3, HC4, HC5, HC6, HS0, Löwdin d‐electron number, agnostic descriptor, reductant redox potential, acid p*K*_a_, and reaction time (Figure 2e,f).

### 2.4 ML Model Performance

**FIGURE 3:**
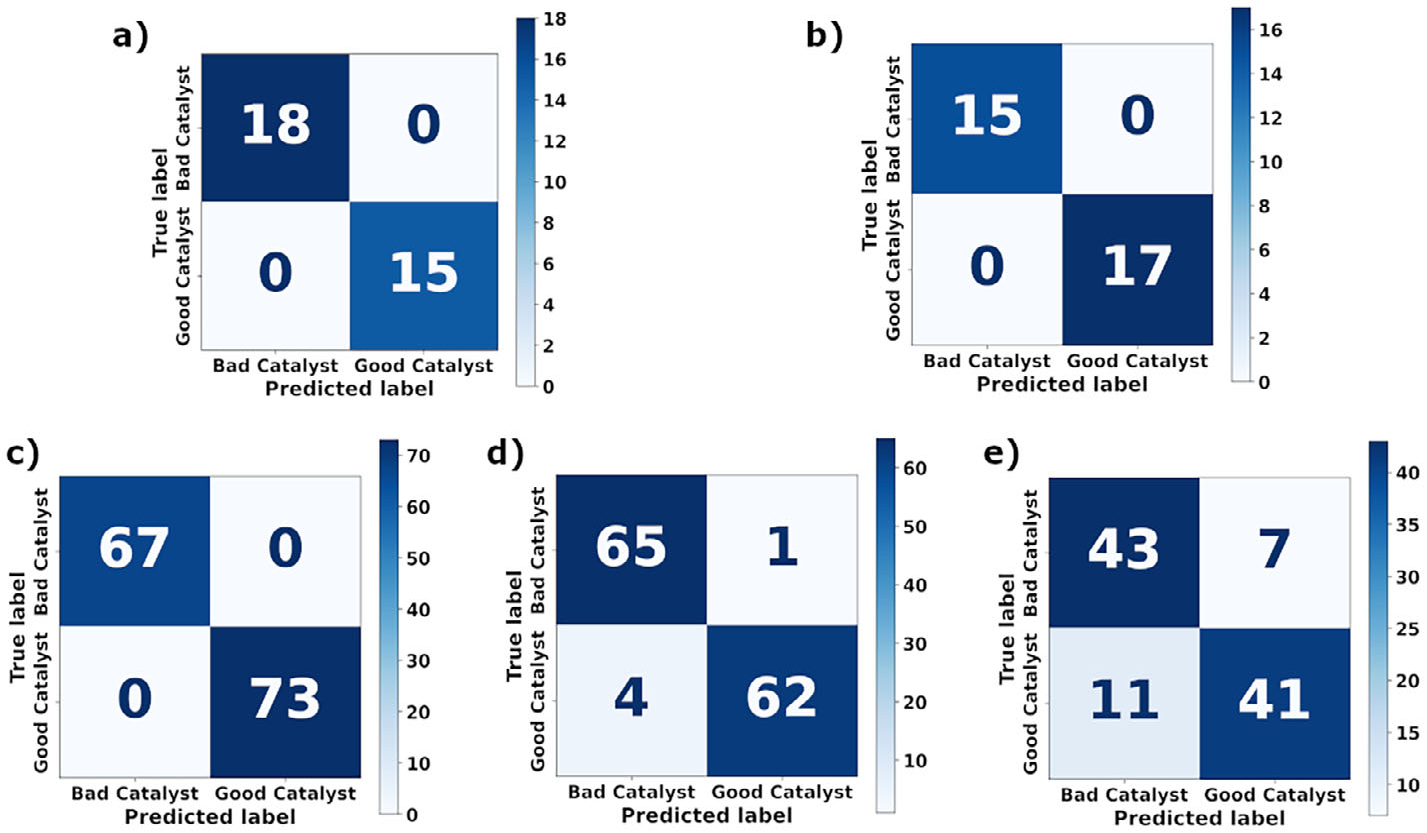
Confusion matrix for the best‐performing classification models on the test set: (a) RF model for reactivity and (b) AB model for stability of family 1 catalysts. (c) DT model for reactivity, (d) AB model for stability, and (e) RF model for selectivity of family 2 catalysts.

Classification targets of family 1 were defined as reactivity (*TOF* > 20 h^−1^) and stability (*TON* > 20 [cat]^−1^). Overall, 10 different classifiers with optimized parameters (Tables S8–S10 and Figure S15) achieved high predictive accuracies, with most models attaining accuracies >0.9 in both the training and test sets. The top‐scoring models included in the workflow are random forest (RF) for reactivity (test accuracy = 1) and adaptive boosting (AdaBoost, AB) for stability (test accuracy = 1). Their excellent accuracy is further confirmed by the confusion matrices (Figure 3a,b), which show perfect class assignments for the test set. Learning curves analysis (Figure S16) showed that, except for the RF model for reactivity exhibiting improved test scores with increasing training set size, the others achieved consistently high training and test scores across all training set sizes, reflecting robust predictive performance. Classification thresholds for family 2 were defined as reactivity (*TOF* > 200 h^−1^), stability (*TON* > 200 [cat]^−1^), and selectivity (TON ratio > 0.75). Using the synthetic minority oversampling technique (SMOTE)‐enhanced dataset (Tables S19–S22 and Figure S22), all 10 classifiers reached test accuracies higher than 0.9 for reactivity and stability. Selectivity prediction yielded lower accuracies, likely because selectivity is a ratio of three factors, which can increase error margins. The best‐scoring models included in the workflow are a decision tree (DT) for reactivity (test accuracy = 1), AB for stability (test accuracy = 0.96) and RF for selectivity (test accuracy = 0.82). Confusion matrices of the DT and AB models (Figure 3b,c) correctly classified the test set. While the RF model misclassified ∼18% of the test set (Figure 3e), with a prevalence of false negative predictions. The RF model for selectivity also exhibited high training but low‐test performance, as indicated by its learning curve (Figure S23), which can be improved with a larger training set to increase generalization ability.

**FIGURE 4:**
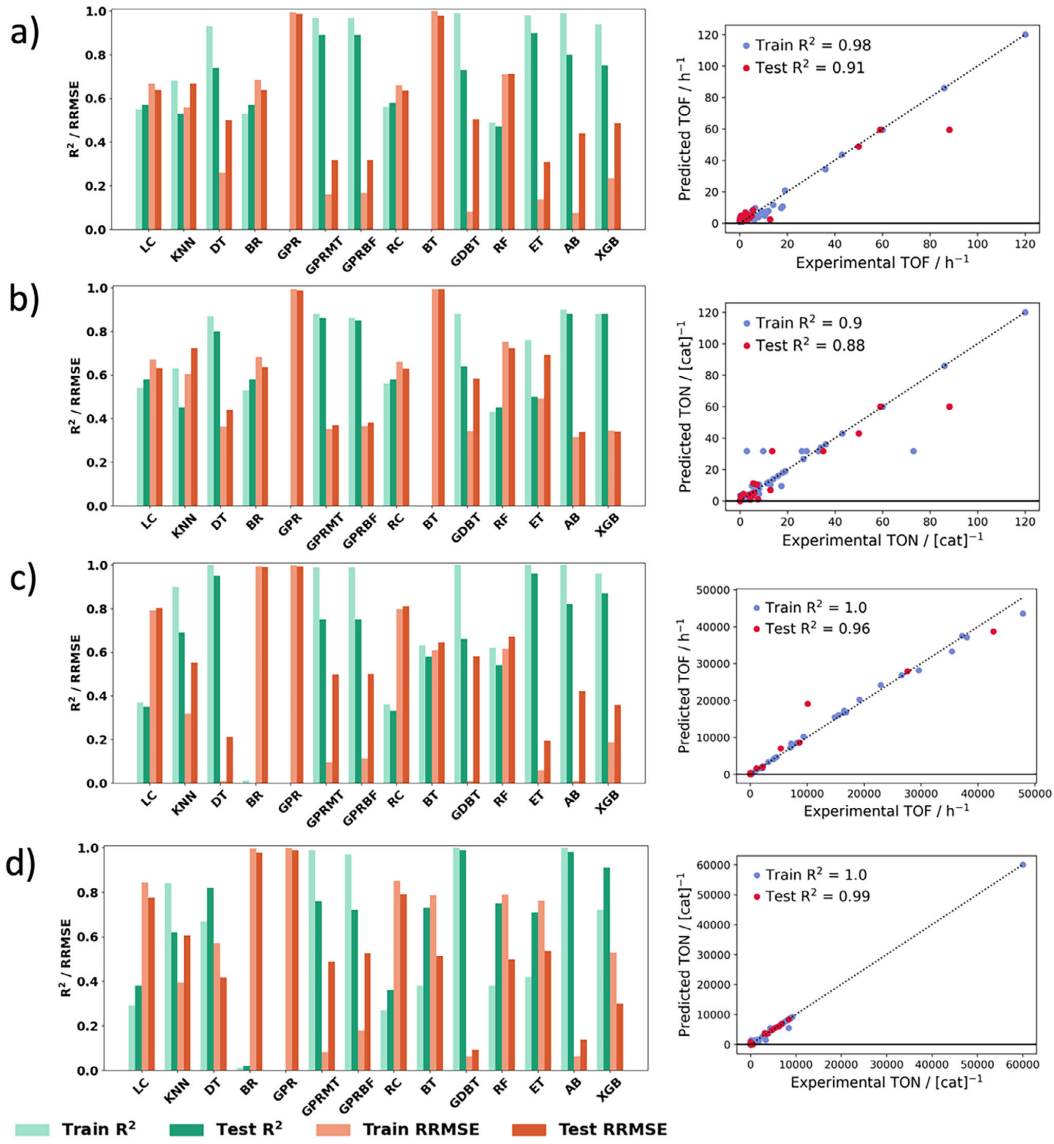
Metrics of all ML regressor models over the train and test set, and scatter plot for the best‐scoring model in each task for (a) TOF of family 1, (b) TON of family 1, (c) TOF of family 2, and (d) TON of family 2 catalysts.

Regression accuracies for TOF and TON exhibited similar trends in both family 1 and family 2 (Tables S11, S12, S23, and S24). In family 1, tree and ensemble models such as DT, extreme gradient boosting (XGB) and AB together with Gaussian process regressors with selected kernels, that is, Gaussian process regression with Matérn kernel (GPRMT) and Gaussian process regression with Radial Basis Function kernel (GPRBF), achieve the best accuracies. Family 2 also showed high accuracies for tree and ensemble models (*R* ^2 ^> 0.82), while Gaussian process regressors yielded acceptable metrics. The MultiLayer perceptron (MLP) regressor performs well for the two families (Figures S17 and S24). We further explored logarithmic scaling (log10(*x* + 1)) of target data and polynomial feature expansion to improve model performance, particularly for linear models (Tables S15, S16, S27, and S28). However, polynomial features capture only simple nonlinearities and are insufficient to model the complex chemical phenomena influenced by multiple experimental factors and a large range of targets. Consequently, their performance is still limited, especially compared to the much higher test accuracy achieved by the MLP model. Overall, the best‐scoring models for family 1 included in the workflow are extremely randomized trees (ET) for TOF prediction (test *R* ^2^ = 0.91) and AB for TON (test *R* ^2^ = 0.88) (Figure 4a,b and Tables S13, S14). These scores are lower than those for family 2, where ET achieved a test *R* ^2^ of 0.96 for TOF prediction, and gradient boosting decision tree (GBDT) reached a test *R* ^2^ of 0.99 for TON, likely due to a smaller training dataset of family 1. However, model performances within family 1 are relatively homogeneous, whereas several models in family 2 predicted quite low metrics, as shown in Figure 4c,d and Tables S25, S26.

Since the range of target values is considerably different in the two families, where TON of 0–120 in family 1 versus 0–9000 in family 2 (Figure S3), we evaluated model performance using several normalized error metrics for a fair comparison: relative mean absolute error (RMAE), relative root mean squared error (RRMSE), and range‐normalized RMSE (NRMSE). These complementary metrics provide an estimate of prediction error across datasets of different scales: RMAE accounts for all the errors equally, while RMSE‐based metrics penalize larger deviations more strongly due to the squared term. In all cases, lower values indicate better performance. In particular, an RRMSE value below 50% indicates a good model performance, reflecting prediction errors that remain low relative to the variability of the target data [68]. For TOF prediction, the family 1 ET model has a test RMAE of 32.3% and a test RRMSE of 30.8%, highlighting good model performance. The close agreement between RMAE and RRMSE suggests that the error distribution is not significantly affected by outliers. Family 2 ET TOF model performs even better, with a test RMAE of 17.8% and a test RRMSE of 19.3%, consistent with its higher R^2^ value and indicating only a mild contribution from outliers. In the AB model for TON of family 1, the test RMAE is 30.9%, and the corresponding RRMSE is 33.9%, again well within the threshold for good model performance. The GDBT for family 2 TON demonstrates excellent predictive capabilities, with a test RMAE of 10.21% and RRMSE of 9.25%. Finally, comparison of the test NRMSE values for the best‐performing models, family 1 (8.27% for TOF ET, 9.07% for TON AB) and family 2 (2.65% for TOF ET, 2.44% for TON GDBT) suggests that the lower accuracies for family 1 models are likely related to the smaller dataset size, which may limit model generalization.

As an additional comparison, we tested the predictive performance using a common feature set for the two families, namely by training Family 1 models with the Family 2 features list and vice versa. The detailed results are extensively reported in Tables S17 and S29, and we observed a steep decline in prediction performance compared to the models presented herein, which highlights the intrinsic differences in the reaction mechanisms between catalyst families. More importantly, we found that experimental conditions significantly impact test accuracy. In family 1, acid equivalents correlated most strongly with TOF and TON (∼0.7), and including them substantially raised the best *R* ^2^ scores from ∼0.5 to ∼0.95 for both reactivity and stability. In contrast, this factor is less important in family 2 catalysts, suggesting that the influence of acid stoichiometry is system‐dependent, potentially related to whether proton transfer occurs in the rate‐determining step or in other key reaction steps. This is consistent with the reaction mechanism proposal by Nishibayashi et al., where, especially for the monomer pathway, the rate‐determining step is predicted to be the N≡N bond breaking [69]. Reaction time also strongly impacts prediction accuracy in family 2. For instance, adding it to the feature set for TOF increased the best *R* ^2^ score from ∼0.53 to ∼0.96, indicating that it encodes additional mechanistic information, such as reaction kinetics or time‐dependent side processes. These observations highlight the importance of accounting for real reaction components, rather than only the catalyst, in predictive ML models.

Overall, we observed poor performances of linear classification and regression models, especially for family 2. This might be related to the vast range of target values and the complexity of the catalytic process, which involves strong non‐linearities and feature interdependence. However, these effects are inherently captured by tree‐ and ensemble‐based models (i.e., ET, AB, GDBT and XGB), which consistently perform best across all tasks. In addition to high *R* ^2^ values, the analysis of normalized error metrics further confirms the validity of these models and indicates a high level of predictive confidence. especially for family 2. We also observed good performance with MLP‐based regression models. Despite the relatively limited dataset size, a shallow architecture with up to two hidden layers was sufficient to train accurate and stable predictive models.

### 2.5 SHAP and PDP‐Based Feature Analysis

**FIGURE 5:**
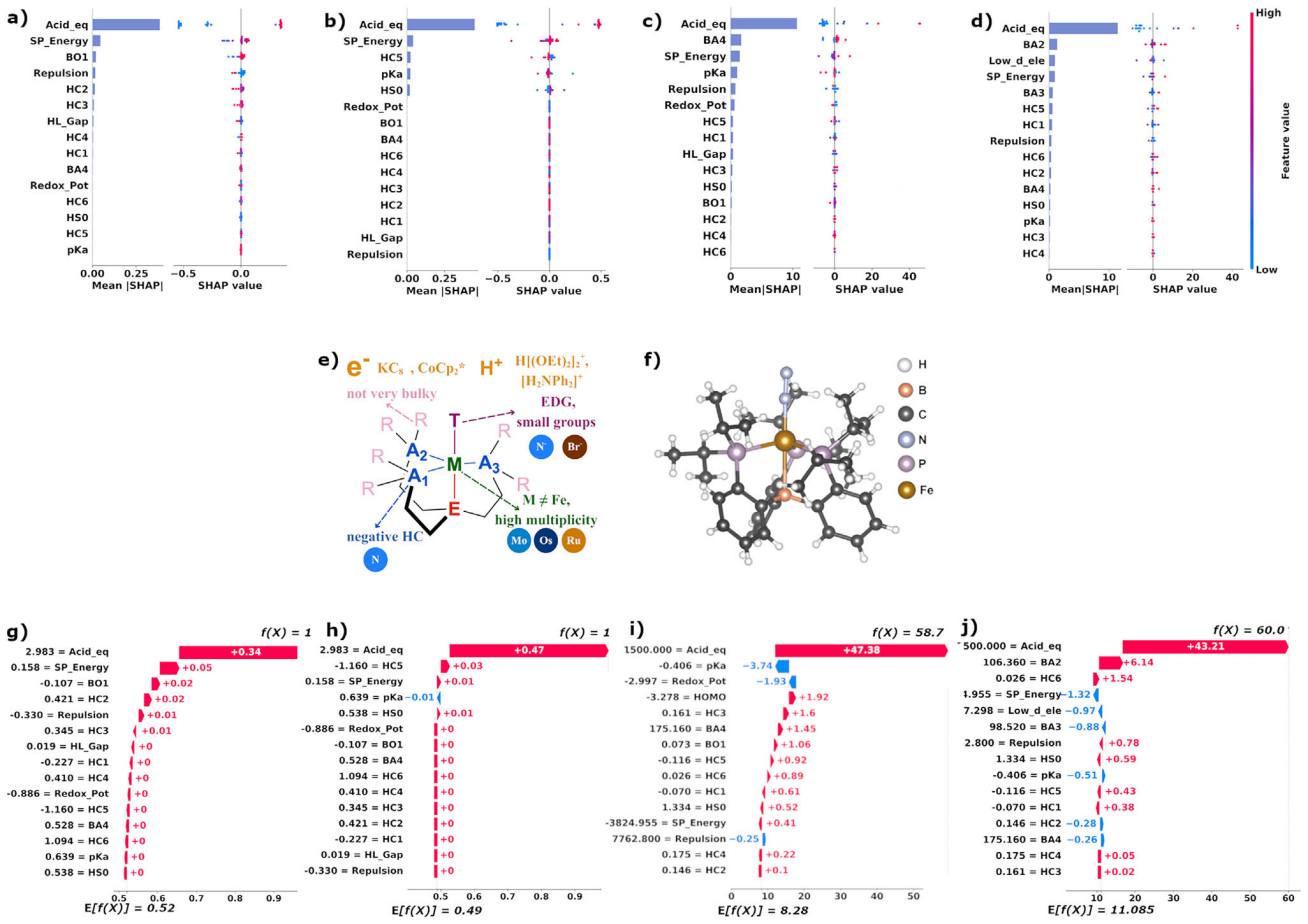
Feature importance and impact on the predicted value by SHAP analysis of the trained ML models for the family 1 catalysts: (a) RF model for reactivity, (b) AB model for stability, (c) ET model for TOF, and (d) AB model for TON. (e) Best values of the most important catalysis features. (f) The structure of the selected Fe catalyst. SHAP waterfall plot to explain ML prediction for the Fe catalyst in: (g) reactivity, (h) stability, (i) TOF, and (j) TON.

To connect the prediction outcome to the molecular feature values, we applied the Shapley Additive exPlanation (SHAP) method to analyze the feature importance in the top‐performance models. By combining SHAP analysis results and the generic structural characteristics of a catalyst family, it is possible to assemble a prototype of an ideal catalyst with maximized performance. In the RF classifier model for reactivity of family 1, the SHAP analysis (Figure 5a–e) showed that the most important feature is the acid equivalents, whose larger values increase the reactivity. Moreover, larger single‐point energies are preferred, which statistically corresponds to complexes with heavier transition‐metal elements, such as Os, Mo or Ru as the metal center. The third preferred feature is lower repulsion energy correlated with higher reactivity, suggesting that smaller ligands are advantageous. Indeed, increased steric hindrance might affect the protonation steps of the reaction by decreasing the accessibility of the nitrogen to the acid. The AB model for stability adds additional insights. Medium‐high values of HS0 indicate that higher multiplicities than the singlet are preferred, while the impact of negative HC5 can be attributed to electron‐rich ligands occupying the top position on the metal center. The ET regression model for TOF prediction highlights further the effects of acid equivalents and SP energy. It also confirms the importance of having a ligand in the top position, as improved TOF corresponds to BA4 at around ∼180°, formed by the top–metal–bottom atoms. AB regression for TON prediction confirms the observed trends, which are further supported by the SHAP analysis of the MLP regressor for TON and TOF (Figure S18).

Partial dependence plots (PDP) analysis was performed to complement the SHAP analysis and obtain further insights. The major strength of PDP analysis is its ability to identify whether the relationship between the target and a feature is linear, monotonic, or a more complex function. Specifically, the most impactful features identified via the PDP analysis of the ET model for TOF (Figure S19) include: higher acid equivalents increase the TOF, an optimal BA4 of approximately 175° and HOMO‐LUMO gaps around 3.25 eV. Other significant impacts are lower repulsion energies and the best redox potential of approximately −3 V (i.e., KC_8_) and −1.5 V (i.e., CoCp*_2_). Dependence of the target on other features is quite low, with limited variation in the model outcome. A similar trend is observed for the AB model for TON (Figure S20), where acid equivalents again have the greatest impact. Acids with p*K*a close to 0, such as H[(OEt)_2_]^2+^ and [H_2_NPh_2_]^+^, improve the TON, as does Löwdin d‐electron count near 5, which potentially identifies oxidized metal states. Higher BA4 and BA2 angles also lead to improved TON, with a small surge observed for BA2 in the 90–100 range. Apart from those, HC has a small influence, for instance, HC2–HC5 values in the 0–0.2 au interval, and negative partial charge for HC1 and HC6 slightly improve TON, with HC6 peaking around −0.2 au and HC1 showing a broader range from −0.4 to −0.1 au.

The SHAP and PDP analysis interpreted that an optimal Peters’ catalyst (Figure 5e) should feature a metal center with high spin multiplicity. The first coordination sphere should avoid bulky ligands to minimize repulsion energies, while the presence of a top group improves performance, as observed from the BA4 PDP trends. Moreover, lateral P atoms (A1, A2, A3 in Table 1) should have a slightly positive charge. Optimal experimental conditions include an acid with near‐zero p*K*a (e.g., H[(OEt)_2_]^2+^ or [H_2_NPh_2_]^+^), a large number of acid equivalents and a reductant, either KC_8_ or CoCp*_2_. These detailed analyses of the global impact of features on the trained models aid performance prediction of targeted catalytic systems using SHAP waterfall plots. In a SHAP waterfall plot for family 1, the prediction starts with an expected value of the classical Fe‐based catalyst (Figure 5f) in diethyl ether with 1500 equivalents of H[(OEt)_2_]^2+^ as acid and KC8 as reductant. Each feature then either increases (positive SHAP value) or decreases (negative SHAP value) the predicted outcome, culminating in the final model output displayed at the top (Figure 5g–j).

In both the RF and AB classifiers, the initial value is ∼0.5. Among all features, large acid equivalents have the highest impact (+0.34 and +0.47, respectively) on the prediction, and this case is correctly assigned as a catalyst with high reactivity and stability, respectively. The ET regressor predicted an initial TOF value of 8.28 h^−1^. Small positive contributions come from several features, such as the Hirshfeld charges of the P atoms (HC2, HC3, and HC4), which comply with the 0–0.2 au range extracted from PDP analysis. Small negative contributions are observed from the repulsion energy, which is in contrast with the PDP observation. More significant negative contributions arise from the reductant redox potential (−1.93) and the acid p*K*a (−3.74). This observation provides insight that strong reductants and strong acids could promote the HER. Despite this, the acid equivalents (+47.38) dominate, resulting in a final predicted TOF of 58.7 h^−1^, in excellent agreement with the experimental value of 59 h^−1^. A similar evolution is observed in the AB TON regressor, albeit with a higher number of negative contributions. In particular, SP energy (−1.28) and BA4 (−0.38) provide a small negative contribution to TON, while positively contributing to the TOF prediction, highlighting the different feature dependencies of TOF and TON. The final TON value delivered by the model is 60 [cat]^−1^, closely matching the experimental value of 59 [cat]^−1^.

**FIGURE 6:**
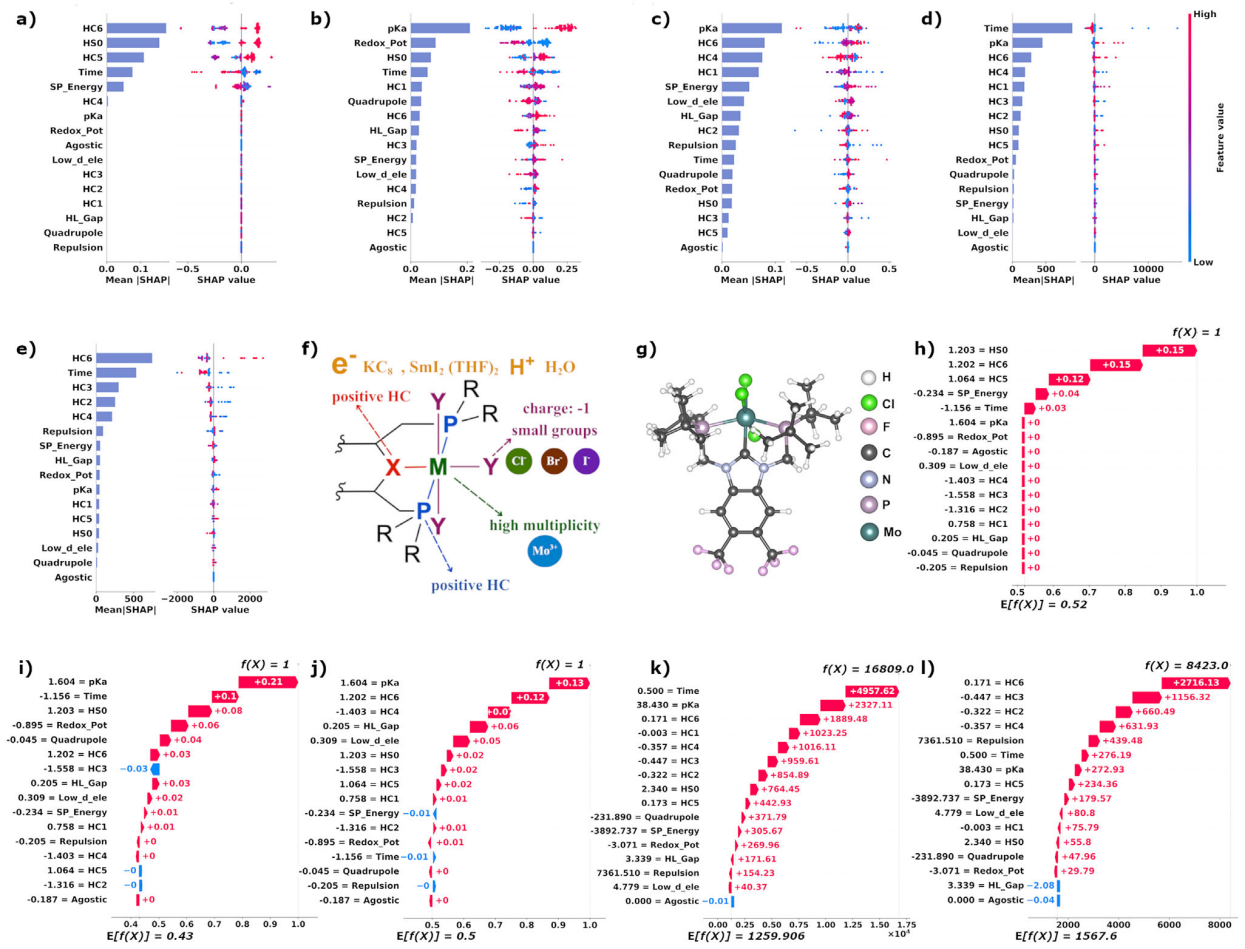
Feature importance and impact on the predicted value by SHAP analysis of the trained ML models for family 2 catalysts: (a) DT model for reactivity, (b) AB model for stability, (c) RF model for selectivity, (d) ET model for TOF prediction, and (e) GBDT model for TON prediction. (f) Best values of the most important catalysis features. (g) The structure of the selected Mo catalyst. SHAP waterfall plot to explain ML prediction for the Mo catalyst in: (h) reactivity, (i) stability, (j) selectivity, (k) TOF and (l) TON.

SHAP analysis for family 2 catalysts (Figure 6a–e) showed that experimental descriptors, such as reaction time, redox potential, and acid p*K*_a_, have a strong impact on the predicted values. The only exception is the DT model for reactivity prediction, where population analysis terms dominate the feature importance. In this model, a larger Hirshfeld spin population at the metal center is preferred, corresponding to metals in a triplet or quartet spin state. In the compiled catalysts, the most common metal, Mo, can access a quartet state only in a +3 oxidation state. Higher reactivity is also associated with medium‐high total energies, indicating monomeric species should be preferred over dimers. Additionally, more positive Hirshfeld charges HC5 and HC6, corresponding to the P atoms of the PXP (X, mostly a C or a N atom) ligand in most of the best‐performing catalysts, correlate with higher reactivity. Thus, tuning the substituents bound to P can help to modulate the partial charge. However, this trend may deviate when the remaining ligands are particularly bulky or when the metal is significantly under‐coordinated. In the AB classifier for stability, the acid p*K*_a_ is the most important feature, with weaker acids improving stability, along with reductants that have a lower redox potential. For catalyst features, high quadrupole moments and HOMO–LUMO gaps hinder catalytic performance, while HS0 and HC6 show a similar trend to that observed for reactivity. Partial charge HC1, which is usually the central one X in the tridentate PXP ligand, also plays a significant role: more positive Hirshfeld charges on this atom improve stability. This charge will likely be affected by substituents on the aromatic ring, suggesting that appropriate introduction of electron‐withdrawing groups at this position can enhance catalyst stability. The RF classifier for selectivity shows trends broadly consistent with those above, exhibiting improved performance with medium‐high p*K*_a_ values and with high‐energy monomeric catalysts. However, unlike the reactivity and stability models, more positive (or negative) HC1 partial charges decrease selectivity, a key divergence in the electronic features governing selectivity relative to reactivity and stability. In the ET regressor for TOF prediction, acid p*K*_a_ emerges as the second most important feature, following reaction time. SHAP analysis also shows a strong relevance of population features. Their impact agrees well with the results observed in these classifier models, while also introducing new insights into the roles of other atoms. In particular, higher TOF values correspond to negative partial charges (∼−0.4 au) on atoms 2, 3, and 4 (HC2–HC4). Comparable conclusions can be drawn on the GBDT model trained for TON prediction and the two MLP models (Figure S25).

PDP analysis of the ET model for TOF (Figure S26) shows trends that are broadly consistent with the SHAP results. Reaction time has the strongest impact, with shorter times leading to exponentially larger TOF values. Acid p*K*_a_ shows a non‐monotonic relationship with TOF, with optimal values obtained for acids with p*K*_a_ around 40 (e.g., water). As observed in SHAP, positive HC5 and HC6 values rapidly increase the predicted TOF, while the opposite trend is observed for HC3 and HC4. HC2 shows a non‐monotonic relation, reaching its maximum positive impact on the TOF value when HC2 of ∼0.3 au. A peak also appears in HS0 and Löwdin d‐electrons number, confirming that higher multiplicity improved TOF values. In particular, HS0 peaks around 2.5 au, which is intuitively intermediate between a triplet and a quartet state. Löwdin d‐electron number less than 5 gives improved TOF, even if the overall impact is small. These values also reflect the interplay between electron‐donating and withdrawing ligands. Given the local environment of the metal and the dataset, these observations are likely due to the Mo^3+^ metal center. Moreover, the SP energy distribution has a peak around −4000 Eh, which refers to high‐energy monomers and agrees with the SHAP analysis for the reactivity classifier. Finally, stronger reductants increase TOF values significantly. The PDP analysis of the GDBT model for TON regression (Figure S27) generally shows more complex feature‐target relationships. Overall, the trends agree well with the SHAP analysis, with the only exception of the reaction time feature. The HC6 and HC5 show a strong increase in TON with partial charges exceeding 0.2 au, while HC2, HC3 and HC4 relationships have peaks at negative charge values. Among these, HC3 displays the strongest effect, with TON maximized when the charge is below −0.4 au. Positive HC1 improved TON. The impact of acid p*K*_a_ and reductant redox potential is small but well aligned with the previous SHAP analysis. Higher TON values can be obtained with reductants, such as SmI_2_(THF)_2_, KC_8_ or CoCp*_2_ and an acid with p*K*_a_ ∼ 40, such as water in THF.

Overall, the SHAP and PDP analysis indicate that the optimal family 2 catalyst (Figure 6f) has a monomeric octahedral geometry, with a high multiplicity metal center, for example, Mo^3+^. The central binding site of the tridentate ligand should have a positive partial charge, while the three monodentate ligands should each have a negative charge. Moreover, the latter groups should bond more closely to the metal than the Mo‐P ones, and the phosphine groups should have a partial positive charge. Finally, the combined computational and experimental descriptors identify the best experimental conditions, being a high p*K*_a_ acid (e.g., water) and either KC_8_ or SmI_2_(THF)_2_ as reductants.

Similarly, we analyzed the model prediction for a selected catalyst (Figure 6g) in THF paired with SmI_2_(THF)_2_ reductant, water acid and a 0.5 h reaction time by means of SHAP waterfall plots (Figure 6h–l). In the DT classifier for reactivity, SP energy and the reaction time have a small positive impact (+0.03), while the HC5 (+0.12), HS0 (+0.15) and HC6 (+0.15) drive the predicted value to 1, corresponding to a good catalyst classification. In contrast, the AB classifier for stability distributes importance across many features, most with marginal positive or negative impact. The three main features are all experimental: the reductant redox potential, the acid p*K*_a_ and the reaction time. Notably, the short reaction time gives a +0.25 increment to the prediction, ultimately assigned to the good catalyst class. It is important to note that the most impactful features in a specific case do not necessarily follow the full model feature importance. For example, the Löwdin d‐electrons number ranks sixth in global importance for the RF selectivity model, whereas in the waterfall plot, it becomes the third feature with a larger positive impact (+0.09). Other major features that drive the predictions toward a good selectivity are HC6 (+0.11) and the acid p*K*_a_ (+0.12).

In the ET regression model for TOF, the initial prediction under the selected conditions is 1259.906 h^−1^. Nearly all the features have a positive impact, with the only exception being the absence of an agostic interaction, which has a marginal effect. As observed above, the Hirshfeld charges play an important role in the prediction. HC6 and HC5 (0.171 au, the P atoms of the tridentate ligand) strongly increase the predicted value, with HC6 alone contributing +1976.27. The Cl^−^ ligands lead to values of HC2, HC3, and HC4 around −0.4 au, yielding an additional cumulative increase of +2724.88. Moreover, the nearly neutral HC1 of the PCP ligand carbon atom further increases the predicted value, in agreement with the PDP analysis. The two larger increments are given by the acid p*K*_a_ (+2442.87) and the reaction time (+4744.47), lifting the prediction to a TOF of 16808.973 h^−1^, which well‐aligns with the experimental values of 16860 h^−1^. In the TON regression model, the expectation value is 1567.6 [cat]^−1^, and the prediction is increased by almost all factors. The strongest contributions arise from the positive Hirshfeld charge on HC6 (+2735.79) and the negative ones on HC2, HC3, and HC4. A smaller positive impact is provided by a short reaction time, which aligns with observations in the SHAP analysis. The final predicted value is 8423.011 [cat]^−1^, in excellent agreement with the experimental value of 8430 [cat]^−1^.

### 2.6 Prediction of Candidate Catalysts

The predictive capability of the models was evaluated by predicting the TOF and TON of 43 reported molecular complexes (Figure S29) that had not yet been included in the NRR dataset, using the three top‐performing models: MLP, ET, and DT for TOF prediction, and GBDT, AB, and MLP for TON prediction. To align with practical considerations, where different catalysts often require distinct experimental conditions, identical experimental conditions were assigned to each candidate based on structure similarity within the database (Table S37). Notably, the predicted trends are highly consistent across different models, with the same seven catalysts repeatedly ranked among the top performers, except in the case of TON predicted by the MLP model (Tables S34, S38, and S39). The predictions also reveal a strong dependence of both TOF and TON on reaction time, in agreement with experimental observations.

The three top predicted candidates possess favorable characteristics for NRR catalysis, consistent with insights from catalyst feature analysis [70]. Among these candidates, two have recently reported experimental data on ChemRxiv, providing additional validation [71]. When predictions are performed under exactly the same experimental conditions as those reported, the order of magnitude of the catalytic metrics is well reproduced (Table 2; Tables S35,S36). For catalysts 22 and 24, a 5 min reaction yields experimental TOFs of ∼37 000 and 54 000 h^−1^, respectively, compared with predicted TOFs of ~30 500 and 31 400 h^−1^. After 2 h, experimental TONs are ∼8500 and 8700, respectively, versus predicted values of ∼9000 and 7600. Normalizing TOF is inherently challenging because TOF values are highest at early reaction times and follow a saturation‐like decay as the reaction progresses. Interestingly, this time‐dependent variability is inherently captured by our models, as training directly on experimental data enables the ML models to reflect such behavior. The ability to capture this intrinsic behavior highlights the robustness and predictive accuracy of the ensemble approach. Overall, these results demonstrate the strong capability of our models to predict NRR catalytic performance for new molecular complexes, providing an efficient and reliable framework for experimental catalyst design and performance screening.

**TABLE 2:** Prediction results of candidate catalysts for the NRR.

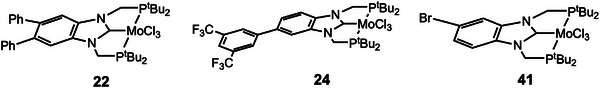				
Candidate catalysts^a^	TOF(NH_3_) (equiv. Mo•^−1^ h^−1^)^b^	TOF(NH_3_) (equiv. Mo•^—1 ^h^−1^)^b^	NH_3_ (equiv. Mo^−1^)^c^	NH_3_ (equiv. Mo^−1^)^c^
	Prediction	Experiment	Prediction	Experiment
22	30535.16	37080 ± 1320	9014.88	8490 ± 400
24	31422.48	54240 ± 6960	7626.47	8740 ± 290
41	30779.67	—	8783.72	—

^a^ The experimental conditions are molybdenum complex, SmI_2_ (28 800 equiv.), H_2_O (28 800 equiv.) in THF solvent at room temperature.

^b^ 5‐minute reactions.

^c^ 2‐hour reactions.

### 2.7 Transfer Learning

**FIGURE 7:**
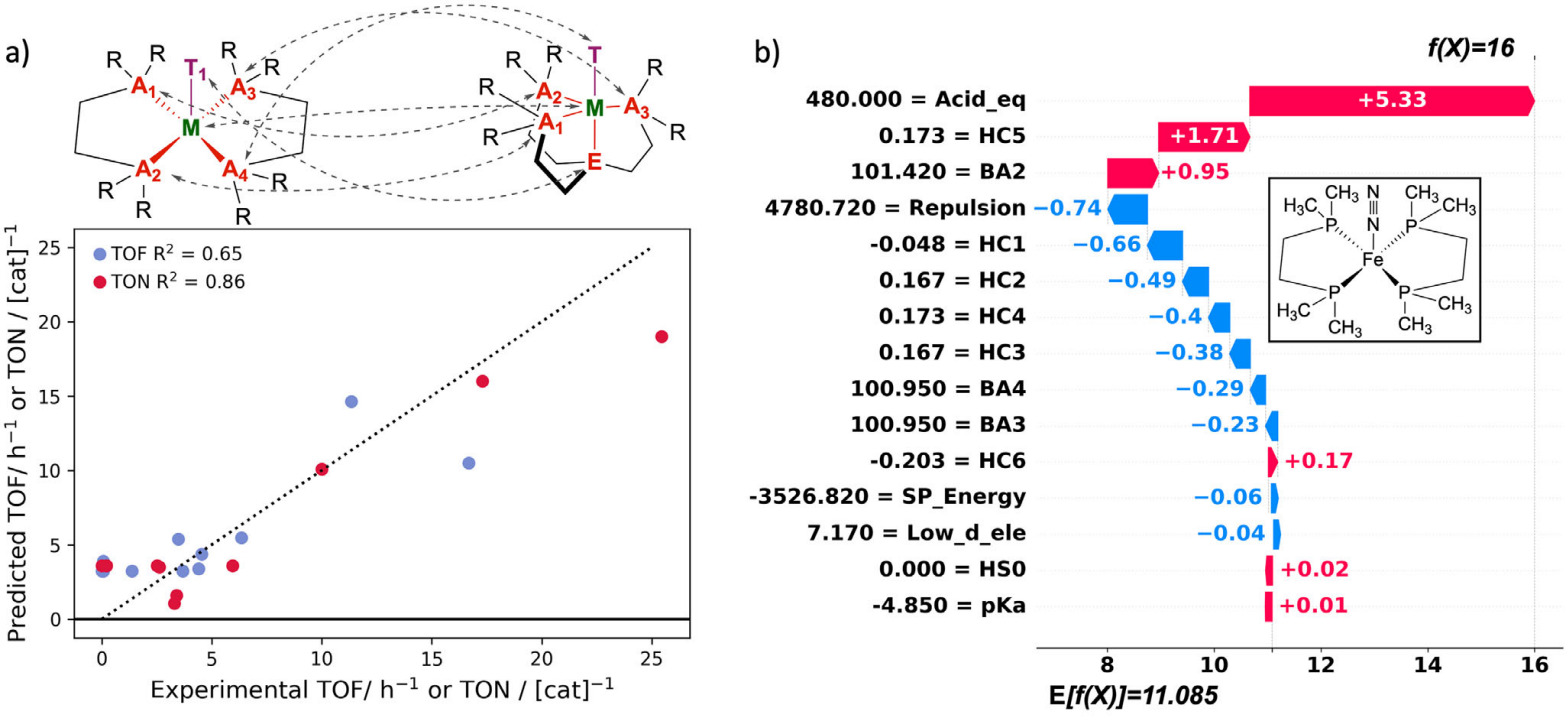
Transfer learning of top‐performance family 1 models to family 3 catalysts: (a) TOF and TON prediction accuracy; (b) SHAP waterfall plots analysis for a representative family 3 catalyst, with its structure shown in the inset.

Given the excellent prediction performance of our models, we further applied the optimal models in the workflow to predict catalytic metrics of family 3 catalysts. It is noteworthy that the validation set used does not overlap with the training or testing set utilized to construct the ML models and provides large coordination topology differences. Predictions on this dataset serve to validate the accurate extrapolation capability of the ML models. Significantly, the predicted TOF and TON achieved *R* ^2^ values of 0.65 and 0.86 (Figure 7a), respectively. These results demonstrate a high degree of consistency between our ML predictions and experimental values. Further confirmation is given by the SHAP analysis (Figure S30) that shows consistent feature importance with the analysis on the family 1 test set (Figure 5). Good accuracy in the transfer learning task was also achieved using a linear regression calibration on top of the pre‐trained MLP models (Figure S31).

Moreover, we analyzed the TON prediction process for an exemplary catalyst of family 3 with the SHAP waterfall plot (Figure 7b). Starting from a value of 11.09 [cat]^−1^, most features give small negative contributions to the model value. Larger contributions (∼−0.5) are given by the small negative charge HC1 and by the positive charge HC2, HC3. The repulsion energy is lower than that of family 1 catalysts and reduces the prediction. The larger BA2 angle given by the N‐Fe‐P atoms, the positive charge of atom 5, and a high acid equivalent increase the predicted value. The final prediction is 16 [cat]^−1^, which is consistent with the experimental value of 17.3 [cat]^−1^. This agreement reflects the universality and accuracy of the ML model's predictions. The efficiency of these models in predicting catalytic activity of a diverse set of geometric frameworks enables rapid screening of highly active NRR catalysts, addressing a long‐standing challenge in identifying superior catalysts from a large pool of candidates.

### 2.8 Model Generalizability

In addition to training independent ML models for each family, which can be directly used to identify design principles within the catalyst class, we also trained a general ML model across all families. The feature set was obtained by combining features from the independent optimized family 1 and family 2 models. The models were trained and tested on a dataset comprising families 1 and 2, and further tested on the family 3 dataset. The TOF models achieve good performance (Table S30), with DF as the best‐performing model, reaching a test *R* ^2^ of 0.95, RMAE of 24.4%, and RRMSE of 21.5%, well within the 50% threshold for reliable performance. TON prediction (Table S31) shows slightly lower accuracies with ET performing best, achieving a test *R* ^2^ of 0.91, RMAE of 29.6%, and RRMSE of 30.2%, which is still identified as a good model. However, in both cases, predictive performance deteriorates significantly when evaluated on the Family 3 dataset, likely due in part to differences in experimental conditions and underlying catalyst structures across families.

To further assess model robustness and reduce the impact of the experimental conditions, we derived an effective activation energy descriptor from the experimental TOF values. In particular, TOF values were normalized by acid and reductant equivalent, and an effective energy barrier was computed by reversely applying the energy span model equation, which can be used to calculate the TOF from computational reaction pathways. This approach requires two main approximations: a single TOF determining state for each reaction pathway and a linear scaling relationship between the acid and reductant equivalents with the TOF. This allows us to train ML models without explicitly including experimental variables. Using this approach, we trained separate models for families 1 and 2. While all tested models for family 2 show some degree of overfitting (Table S33), with the best‐performing model AB achieving a test *R* ^2^ of 0.82 and a low RMAE of 5.39%. Notably, SHAP analysis of this AB model (Figure S28) is overall consistent with that obtained from models with experimental conditions, supporting their ability to learn intrinsic structure–property relationships, instead of trivial correlations with experimental variables. In contrast, family 1 models show lower accuracy, with a maximum test *R* ^2^ of 0.62 achieved by the ET model.

### 2.9 Computational Workflow

To facilitate the rapid simulation and screening for new catalyst design, we developed a streamlined computational workflow for efficient modelling and ML prediction of catalytic performance of metal–ligand complexes for NRR. In this method, users only need to provide the SMILES representation of coordination ligands to automatically generate the corresponding complex structures, from which the predicted catalytic metrics will be output.

**FIGURE 8:**
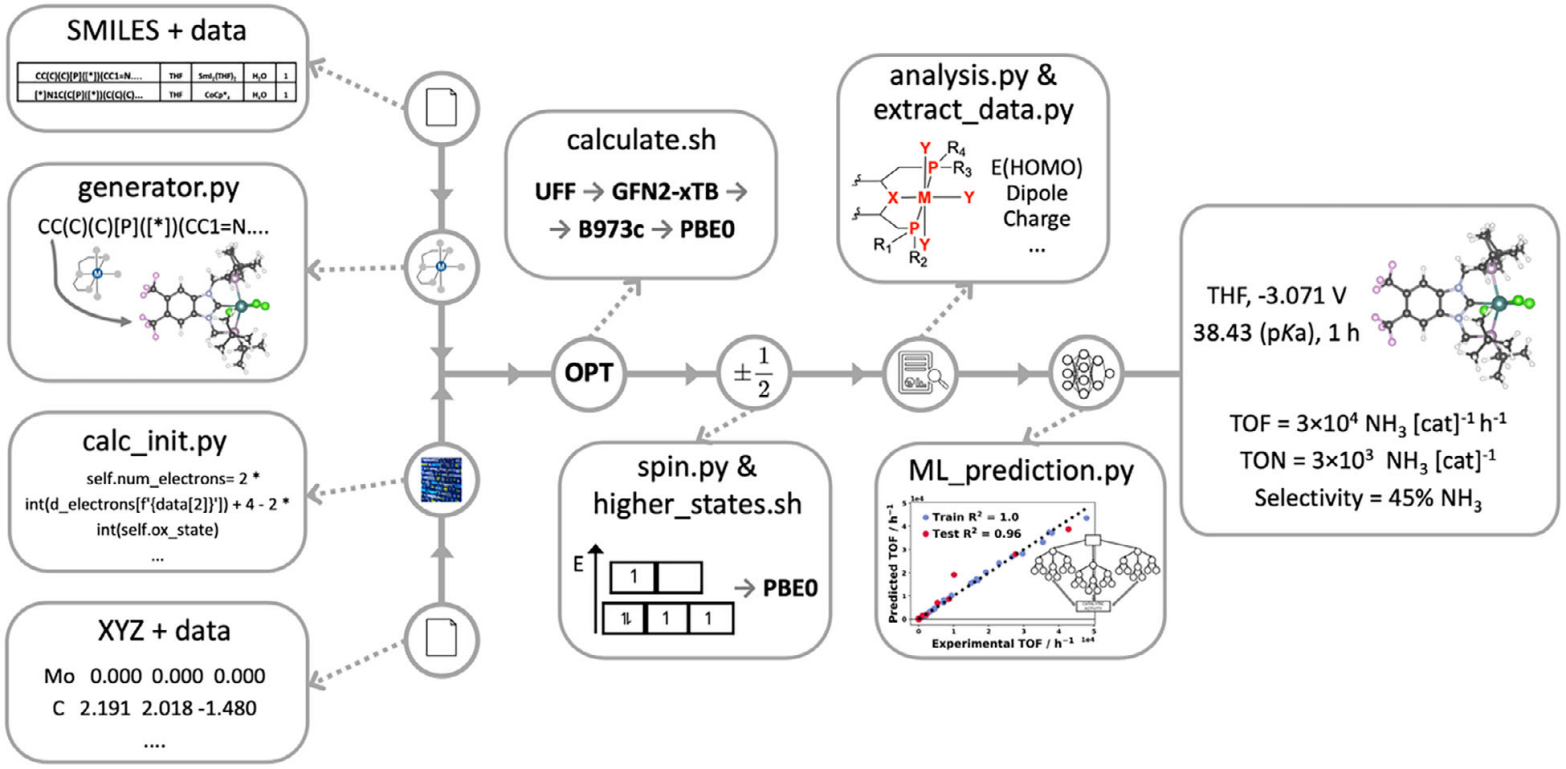
Flowchart of the developed workflow for NRR catalyst evaluation.

The SMILES‐based input provides a potentially valuable route for future development of more efficient and accurate approaches, for instance, when implemented with deep learning models such as graph neural networks, rather than relying on quantum chemical descriptors, which are accurate and interpretable but computationally expensive. It also allows users to input existing XYZ coordinate files, making the workflow easily applicable to structures that differ from the predefined topologies. The entire workflow has been tested on a supercomputer with 32 cores with high efficiency and smoothly. More importantly, the workflow is built on open‐access databases to support transparent, extensible catalyst design. The workflow contains four main sections (Figure 8):

#### 2.9.1 Structure Generation

This section allows the creation of coordination compounds based on the most common topologies of categorized NRR catalysts. The XYZ structure of a potential catalyst is generated from a simple input file specifying the following: structure name, metal atom, solvent name, total molecular charge, metal formal oxidation state, SMILES of each ligand with appropriate dummy atoms and experimental conditions to use in the ML prediction.

#### 2.9.2 Structure Description

Quantum chemical calculations are initially carried out with a multilevel geometry optimization (UFF, GFN2‐xTB, B97‐3c and PBE0‐D4/def2‐SVP) and a final single‐point calculation (PBE0‐D4/def‐TZVPD) in the lowest possible spin multiplicity. Other possible spin multiplicities for the complex are also assessed based on the metal atom and its oxidation state, following crystal field theory, and additional quantum chemical calculations are performed for each of them. It is noteworthy that all quantum chemical calculations include implicit solvation effects by CPCM.

#### 2.9.3 Data Analysis

The results at the most stable multiplicity state from the single‐point calculations are parsed, and molecular descriptors (Table S5) are extracted.

#### 2.9.4 ML Prediction

The extracted data are used by the highest‐ranking trained regressor and classification models to predict the catalytic activity parameters.

## 3 Conclusion

In this work, we developed a computational workflow that integrates quantum chemical calculations and ML techniques to accelerate the prediction of catalytic performances of metal–ligand complexes. Unlike conventional studies, this method directly targets experimental catalytic metrics and was validated against a large, compiled database of experimentally reported results. Through systematic feature analysis and feature set tuning, we trained classifiers to predict catalyst reactivity and stability, and regressors to quantitatively predict TOF and TON. For classification, the RF and AB models achieved high test accuracies for reactivity and stability, respectively, in family 1 catalysts, while the DT and AB models achieved similar strong test accuracy for family 2 catalysts. In addition, the RF classifier for selectivity in family 2 attained a good test accuracy. For regression tasks, the ET model for TOF and the AB model for TON achieved strong test performance in family 1. Even higher test scores were observed for family 2, with *R* ^2^ of 0.96 for TOF (ET) and 0.99 for TON (GDBT). Feature analysis further elucidates that optimal performance of family 1 catalysts is associated with a high spin multiplicity metal center, a four‐valence bottom atom, minimal steric repulsion in the first coordination sphere, and a top group. Slightly positive charges on lateral P atoms were also found to be beneficial. The optimal family 2 catalyst favor a monomeric octahedral geometry with a high multiplicity metal center, a positively charged central binding site on the tridentate ligand, and negatively charged monodentate ligands that bind more strongly to the metal than Mo–P groups. Finally, the combined computational and experimental descriptors identify the best experimental conditions.

These detailed analyses of the global impact of features enabled accurate prediction of catalytic performance, with predicted values in excellent agreement with the experimental values for both catalyst families. More importantly, when applied to previously untrained molecular complexes, the models successfully reproduced the correct order of magnitude and the relative performance trends among catalysts. The models also captured the strong time‐dependent variability of TOF and TON, highlighting the robustness and predictive accuracy of the ensemble ML approach. To further validate model generality, we evaluated transfer learning performance on a structurally distinct set of family 3 catalysts. Despite the large difference in structural scaffolds relative to the training data, the models achieved *R* ^2^ values of 0.86 for TON and 0.65 for TOF, demonstrating strong transferability and universality. To accelerate this design process, we developed a streamlined workflow that predicts the catalytic performance of new catalysts from SMILES input and enables the design of new catalysts via integration with an open‐access database. Overall, this work offers an efficient and experimentally grounded framework for discovering high‐performance NRR catalysts under realistic conditions and provides insights into the design of other electrocatalysts. More importantly, it provides a promising route for future development of more efficient and accurate approaches, for instance by integrating the workflow with deep learning models such as graph neural networks to enable rapid and accurate prediction of new NRR catalysts. In addition, this framework can be extended to large‐scale computational screening using open‐access datasets of organic ligands or metal–ligand complexes, and could be designed to be integrated with automated experimentation and closed‐loop experiment–model feedback workflow. Together, these developments have the potential to further advance ammonia synthesis research through ML‐driven design methods for bio‐inspired catalysts discovery.

## 4 Methods

### 4.1 Quantum Chemical Calculations

The quantum chemical method for geometry optimization was benchmarked using six complexes. We tested several DFT functionals, including: PBE0 [72], B3LYP [73, 74], M06 [75], M06‐L [75], M06‐2X [75], TPSS [76], TPSSh [76], wB97XD [77], B97‐3c [78], r2‐SCAN‐3c [79] with empirical dispersion corrections (either D3 [80] or D4 [81]) and implicit polarizable solvation. The methods have been assessed on accuracy, expressed as RMSD against crystallographic structures, and computational efficiency. Furthermore, we evaluated three different multilevel structure optimization processes starting from the SMILES strings of the complex and going up to DFT calculation. Overall, PBE0‐D3 performed best for geometry optimization among the tested DFT functionals, and the best‐performing pipeline of methods was: universal force field (UFF) [82], GFN2‐xTB [83] with GBSA solvation model, B97‐3c with CPCM solvation model, and PBE0‐D4/def2‐SVP with CPCM solvation model. This multi‐step optimization protocol improves structural accuracy relative to crystallographic structures while providing a threefold speedup over direct PBE0‐D4 optimizations. Therefore, every structure was subjected to this optimization workflow to obtain the lowest possible spin multiplicity, while other spin multiplicities were optimized using the lower state optimized structure as a starting guess for the PBE0‐D4/def2‐SVP optimization. More details on the benchmarking and calculation procedure are available in the Supporting Information. Finally, for each optimized structure, a single‐point calculation was carried out at the PBE0/def2‐TZVPD level of theory. The method was applied to calculate the absolute redox potential of the ferrocene/ferrocenium (Fc/Fc^+^) reference electrode in THF, which is not available in the literature, and is consistent with previously reported computational and experimental values in other solvents [84, 85]. Using this reference, the method reproduced experimental redox potentials for a subset of six molecules (Table S4), achieving good agreement with an average deviation of −0.098 V. Furthermore, it showed high accuracy in calculating acid p*K*_a_ values when compared with experimental values, with an *R* ^2^ score of 0.98 (Figure S8). All the DFT calculations have been performed in ORCA 6.0.1 [86, 87] using the CPCM solvation model.

### 4.2 ML Models

In order to determine the most efficient ML algorithm for the catalytic performance prediction of the metal–ligand catalysts, we included different regressors and classifiers, including logistic regression [88] (LR), k‐nearest neighbors (KNN), Gaussian naive Bayes [89] (GNB), Gaussian process [90] regressors with different kernels (GPR, GPRMT, GPRBF), Lasso [91] CV (LC), Ridge CV (RC), ElasticNet CV (EN), Bayesian regression [92] (BR), support vector [93] classification (SVC), DT [94], RF [95], ET [96], bagged trees regressor [97] (BTR), GBDT [98], AB [99], and XGB [100]. Additionally, a neural network‐based model, MLP [101], was included in the regression tasks. Classification models have focused on three indicators of catalytic performances: reactivity (TOF), stability (TON), and selectivity toward NRR (TON $\mathrm{ratio}\;\;=\frac{\mathrm{TON}(NH_{3})}{\mathrm{TON}\left(NH_{3}\right)+\;\mathrm{TON}\left(H_{2}\right)}\;$). Regressor models have been employed to predict the values of TOF and TON of NRR. Herein, the hyperparameters of each algorithmic model were optimized by grid search with nested 10‐fold cross‐validation (CV) to prevent training overfitting. The optimized hyperparameters for the selected models are reported in Tables S7 and S18. We applied a number of statistical metrics, including *R* ^2^, explained variance, MAE (mean absolute error), MSE (mean squared error), RMSE (root mean squared error) scores to assess the regression models; and accuracy, precision, recall and F1 scores, to evaluate the performance of each classification model. The SMOTE method was used for data enhancement, considering the imbalance of the data samples for the classification models. Its core idea is to generate new synthetic samples for the minority classes by interpolating minority‐class samples, thereby alleviating the model bias caused by the class imbalance. Specifically, it identifies the k‐nearest neighbors of each minority‐class instance and generates new samples along the line segments joining the instance and its neighbors. This augmentation enhances the representation of minority classes during training and helps improve the generalization ability of the models. SMOTE is a well‐established and robust method, previously applied in several ML models for chemical applications [102, 103]. However, in some cases, the introduction of synthetic data might introduce unphysical artefacts and obstruct the definition of clear class boundaries [104]. The 80: 20 train‐test split ratio for all ML studies we carried out was selected after evaluating several train‐test splitting possibilities on a small subset of ML models for TOF regression. Finally, we applied several feature analysis techniques, such as: Pearson correlation coefficient matrix, Spearman's rank correlation, PCA, PDP and SHAP [105] analysis. All the ML tasks have been performed using the 1.6.1 version of the scikit‐learn [106] Python library, ensuring reproducibility.

## Funding

This research was supported by the Swedish Research Council for Sustainable Development, Sweden (N 2023‐01559) and the National Natural Science Foundation of China (22571107).

## Conflicts of Interest

The authors declare no conflicts of interest.

## Supporting information




**Supporting File**: adma73603‐sup‐0001‐SuppMat.docx.

## Data Availability

The data that support the findings of this study are available from the corresponding author upon reasonable request.
