# Supplementary material for: Machine Learning Accelerated Computational Design of Bio‐Inspired Catalysts in the Nitrogen Reduction Reaction
Source: Adv Mater. 2026 Jun 6;38(39):e73603. doi: 10.1002/adma.73603 (PMC13361151; doi:10.1002/adma.73603)
Supplement: Supplementary file 1 — Supporting File: adma73603‐sup‐0001‐SuppMat.docx. [file ADMA-38-e73603-s001.docx]

**Supplementary file for**

**Machine learning accelerated computational design of bio-inspired catalysts in the nitrogen reduction reaction**

Leonardo Di Ciano,^1^ Zihan You,^1,2^ Haoran Chen,^3^ Qifan Zhong,^2^ Rong-Zhen Liao,^3^
Shaoqi Zhan^1^*

^1^Department of Chemistry-Ångström Laboratory, Molecular Biomimetics, Uppsala University, 75120, Uppsala, Sweden.

^2^School of Metallurgy and Environment, Central South University, Changsha 410083, China.

^3^School of Chemistry and Chemical Engineering, Huazhong University of Science and Technology, Wuhan, 430074, China.

Table of Contents

[1 Compiled catalytic systems 3](#_Toc230083642)

[1.1 Dataset composition and examples 3](#_Toc230083643)

[1.2 Catalyst categories and topologies 5](#_Toc230083644)

[2 Quantum chemical calculations 7](#_Toc230083645)

[2.1 Geometry optimization 7](#_Toc230083646)

[2.2 Redox potential and pK_a_ 10](#_Toc230083647)

[3 Chemical properties calculation and analysis 13](#_Toc230083648)

[3.1 Data extraction 13](#_Toc230083649)

[3.2 Property analysis 14](#_Toc230083650)

[4 Machine Learning Protocol 16](#_Toc230083651)

[5 Additional results for family 1 (Peters’) catalysts 19](#_Toc230083652)

[5.1 Feature analysis and selection 19](#_Toc230083653)

[5.2 Classification models for family 1 (Peters’) catalysts 20](#_Toc230083654)

[5.3 Regression models for family 1 (Peters’) catalysts 24](#_Toc230083655)

[5.4 Regression of Family 1 dataset with Family 2 features list 30](#_Toc230083656)

[5.5 Additional SHAP and PDP analysis on family 1 (Peters’) catalysts 31](#_Toc230083657)

[6 Additional results for family 2 (Nishibayashi’s) catalysts 33](#_Toc230083658)

[6.1 Feature analysis and selection 33](#_Toc230083659)

[6.2 Classification models for family 2 (Nishibayashi’s) catalysts 34](#_Toc230083660)

[6.3 Regression models for family 2 (Nishibayashi’s) catalysts 38](#_Toc230083661)

[6.4 Regression of Family 2 dataset with Family 1 features list 44](#_Toc230083662)

[6.5 Additional SHAP and PDP analysis on family 2 (Nishibayashi’s) catalysts 45](#_Toc230083663)

[7 Combined models 47](#_Toc230083664)

[8 Energy span regression models 48](#_Toc230083665)

[9 Prediction of candidate catalysts 49](#_Toc230083666)

[10 Transfer learning 55](#_Toc230083667)

[11 Overall workflow 56](#_Toc230083668)

[12 References 59](#_Toc230083669)

# Compiled catalytic systems

## Dataset composition and examples


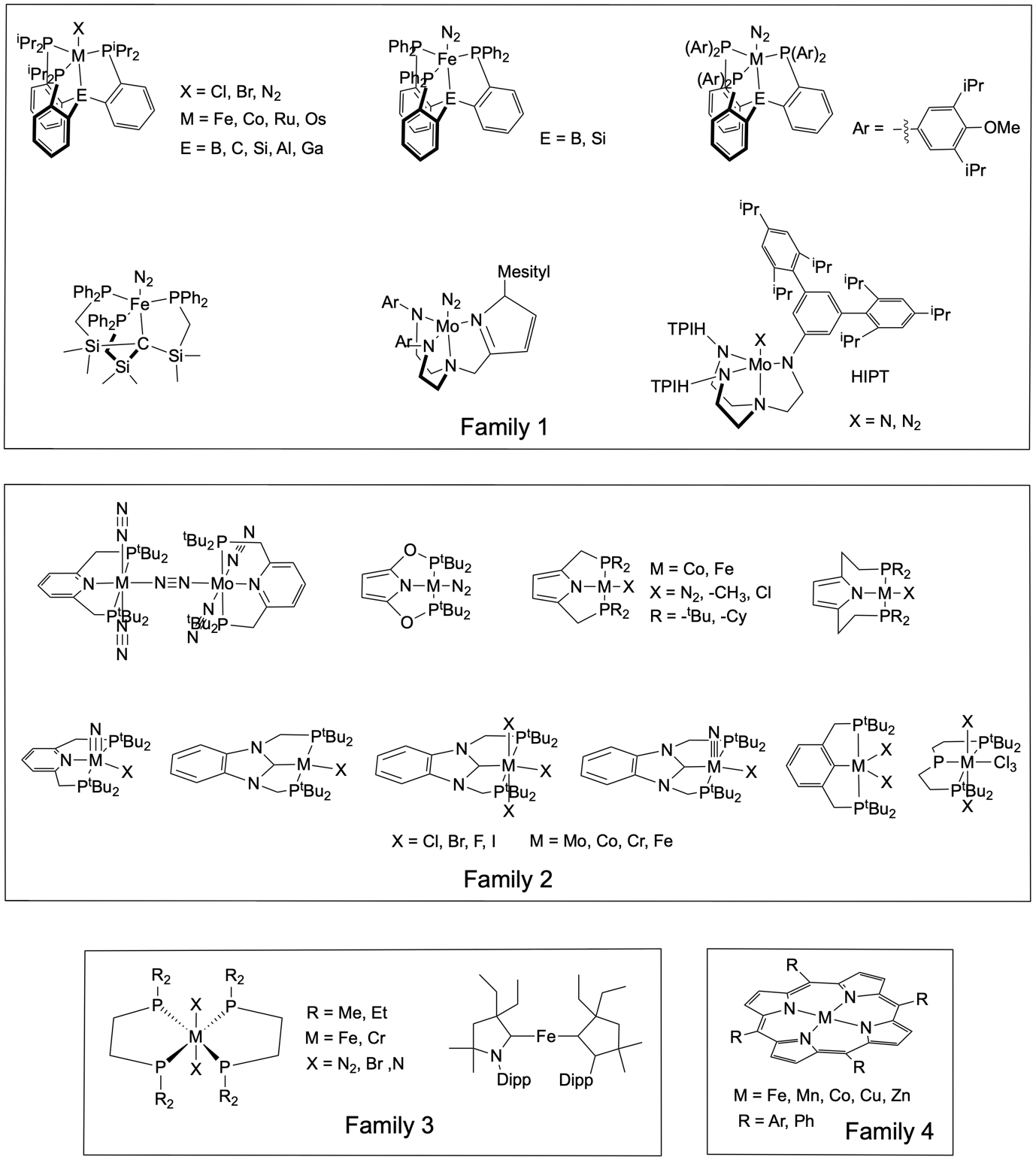


**Figure S1.** Examples of structures of the reported molecular catalyst for the nitrogen reduction reaction.

**Table S1.** Summary of the compiled catalytic systems for the nitrogen reduction reaction.

|  | Family 1  (Peters’ catalyst) | Family 2  (Nishibayashi’s catalyst) | Family 3 and 4 |
| --- | --- | --- | --- |
| Collected experimental dataset | Catalyst structure and its quantity; Solvent; Acid and its quantity; Reductant and its quantity; Temperature; Reaction time; TOF; TON and NH_3_ Yield | Catalyst structure and its quantity; Solvent; Acid and its quantity; Reductant and its quantity; Temperature; Reaction time; TOF; TON and NH_3_ and H_2_ Yield | Catalyst structure and its quantity; Solvent; Acid and its quantity; Reductant and its quantity; Temperature; Reaction time; TOF; TON and NH_3_ and H_2_ Yield |
| Catalysts | 103 entries, 36 catalyst structures | 391 entries, 167 catalyst structures | 30 entries, 15 catalyst structures |
| Acids | (CH_2_OH)_2_, H_2_O, [Li][NTf_2_], [H(OEt_2_)_2_]BArF_4_, HOTf, [LutH]BArF_4_, HCl, [H(OEt_2_)_2_]BArF_4_, [H_2_NPh_2_]OTf, [PhNH_3_]OTf, [H_3_N-2,5-Cl_2_C_6_H_3_]OTf, [N-Me-H_2_NPh]OTf, [ColH]BArF_4_ | [3,5]-LutH, CF_3_CH_2_OH, CH_3_OH, ColH, PyH, HPnBu_3_, H_2_O, H_2_SO_4_, HOCH_2_CH_2_OH, H(OEt_2_)_2_, HOTf, HPCy_3_, HPtBu_3_, iPrOH, [N-Me-H_2_NPh]^+^, LutH, Cl-LutH, Me-LutH, PhNH_3_, H_2_NPh_2_, PhOH, PicH, tBuOH, [H_3_N-2,5-Cl_2_C_6_H_3_], HCl | (CH_2_OH)_2_, H_2_O, HCl, [H_2_NPh_2_]OTf |
| Reductants^a^ | CoCp_2_, CoCp*_2_, CrCp_2_, CrCp*_2_, Sml_2_(thf)_2_, KC_8_, K, Na/Hg, Cr(C_6_H_6_)_2_ | CoCp_2_, CoCp*_2_, CrCp_2_, CrCp*_2_, SmI_2_(thf)_2_, KC_8_, K, Na/Hg, Cr(C_6_H_6_)_2_ | CoCp*_2_, Sml_2_(thf)_2_, KC_8_, |
| Solvents | Diethylether, benzene, heptane, THF | Toluene, THF, diethylether, hexane, MTBE, DMSO, ethanediol, MeCN, dioxane | Diethylether, THF, H_2_O |

^a^ In the absence of an external reductant, the catalyst redox potentials were used.


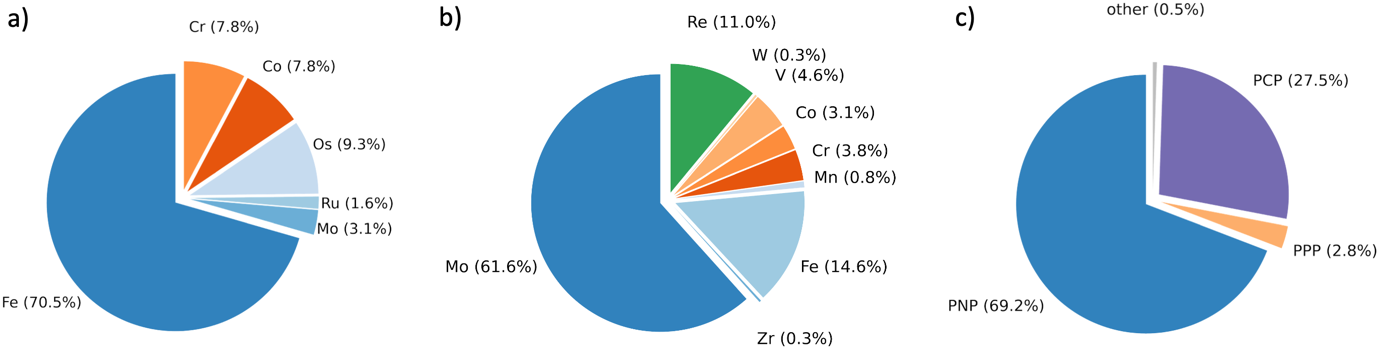


**Figure S2.** Analysis of the compiled catalyst categories. a) Metal center distribution in family 1, 3 and 4 catalysts. b) Metal center distribution in family 2 catalysts. c) Distribution of tridentate ligand binding sites in family 2 catalysts. Definition of family: see 1.2 Catalyst categories and topologies.


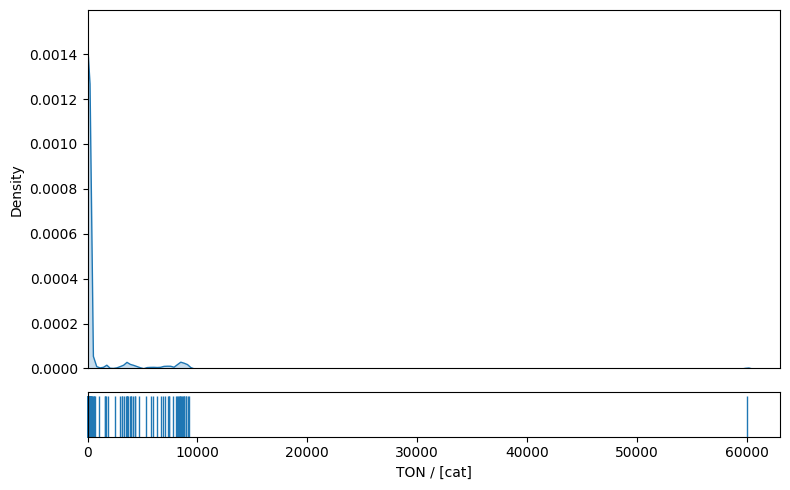


**Figure S3.** Distribution plot of the NH_3_ TON in the data set. The parameter values are highly dispersed in the compiled dataset, ranging from 1 to 10000, with a few data points reaching up to 60000. However, the majority of the data points fall below 50.

## Catalyst categories and topologies

The compiled 218 catalysts are categorised into four families based on their geometric structures, as shown in Table S2.

Family 1 complexes with a topology designated by the ID BK in our established workflow. The topology has two sub-types:

• 0TOP, without a ligand on top of the metal atom;

• 1TOP, with an additional ligand on top of the metal atom.

Family 2 complexes are extracted from the database of Nishibayashi’s catalysts. They all share tridentate ligands, and four different topologies are available:

• OBD, which has an octahedral dinitrogen-bridged dimer topology, with two dinitrogen substituents on each metal atom;

• OS, which has a single octahedral topology, with three halogen ligands on the metal atom;

• SP, which has a square pyramidal topology, with a nitrogen atom and a halogen ligand on the metal atom;

• SC, which has a square planar topology, with a monodentate ligand on the metal atom.

Any of these topologies has three subtypes: PCP, PNP and 5PNP (PNP with pyrrole ring) type.

Family 3 complexes have a topology invoked by the ID DBD. The topology has three sub-types:

• 0TOP, without a ligand on top of the metal atom

• 1TOP, with an additional ligand on top of the metal atom

• 2TOP, with an additional ligand on top and another one on the bottom of the metal atom

Family 4 complexes utilize a topology that is invoked by the ID TET. It can handle most of the porphyrin family ligands. The topology has two sub-types:

• 0TOP, without a ligand on top of the metal atom

• 1TOP, with an additional ligand on top of the metal atom

**Table S2.** Categorization of catalysts into four families, including their geometric structures, topologies, and subtypes.

# Quantum chemical calculations

## Geometry optimization

The quantum chemical method was benchmarked for geometry optimization using a subset of complexes: a Co monomer (Figure S4a) and a Mo dimer (Figure S4b) developed by Nishibayashi^1,2^, a Cr catalyst (Figure S4c) developed by Mock^3^, a Mo catalyst (Figure S4d) developed by Schrock^4^ , and two Fe catalysts (Figure S4e-f) developed by Peters^5^. The DFT functionals evaluated include: PBE0^6^, B3LYP^7,8^, M06^9^, M06-L^9^, M06-2X^9^, TPSS^11^, TPSSh^10^, wB97XD^11^, B97-3c^12^, r2-SCAN-3c^13^ with empirical dispersion corrections (either D3 or D4) and implicit polarizable solvation. Optimized structures were compared with the X-ray crystallography structure taken from the references, and accuracy was determined with two types of RMSD parameters computed with PyMOL^14^. Additionally, the CPU time required for per optimization was recorded to quantify the efficiency of the geometry optimization process. A lower RMSD means that the structure matches more closely the experimental references, while a lower CPU time means reduced computational resources.

For the Mo complexes, the first one considered only the alignment of the core atoms of the structure, which are Mo, N and P. While the second value considers the RMSD for all the atoms, except for hydrogens. The reason to evaluate the two different parameters lies in the presence of tert-butyl substituents on the P, which are the more flexible part of the complex and hence more affected by the difference between the crystal and the solvent environment. In the dimeric Mo complex, the benchmark was carried out in both the singlet and triplet spin states. The RMSD values between the calculated and crystallographic structures for the complex are reported in Figure S5.


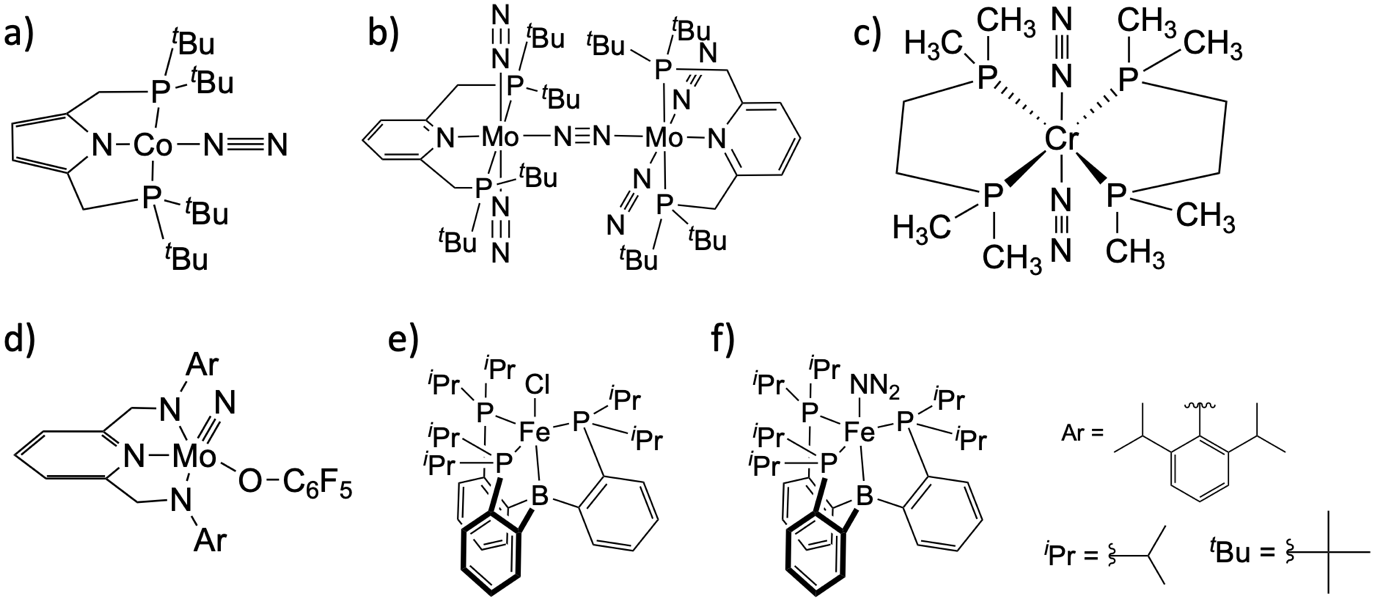


**Figure S4.** Molecular structures of catalysts employed in the geometry optimization benchmark.

For complexes c and d, the RMSD and CPU time were compared after geometry optimization using the functionals PBE0, B3LYP, M06-L, and wB97XD in solvent with the Def2-SVP basis set. The results show that PBE0-D3 is the most suitable functional for complex c, with the RMSD of 0.137 and a CPU time of 9.71 h. For complex d, M06L performs best, with an RMSD of 0.348 and a CPU time of 64.05 h. For the Fe-Cl catalysts (Figure S4e), in the CPCM (Et_2_O) solvent phase, the ranking for functionals for quartet-state optimizations is accuracy: M06L-D3 > TPSS-D3> PBE0-D3 > PBE0 > wB97XD > B3LYP-D3 > M062X-D3, and efficiency: M06L-D3 > TPSS-D3 > B3LYP-D3 PBE0-D3 > PBE0 > wB97XD > M062X-D3. So, the optimal functionals include M06L-D3, TPSS-D3, and PBE0-D3. For the Fe-NN_2_ catalysts (Figure S4f), PBE0-D3, and TPSS-D3 in Et_2_O show good performance in accuracy and efficiency. Overall, PBE0-D3 (hybrid GGA) and TPSS-D3 (meta-GGA) functionals with the Def2-SVP basis set and UltraFine grid in Et_2_O are the optimal DFT method for Peters’ Fe catalysts. Hence, based on these tests on four different metal complexes, PBE0-D3 is chosen as the most suitable DFT functional for geometry optimization.


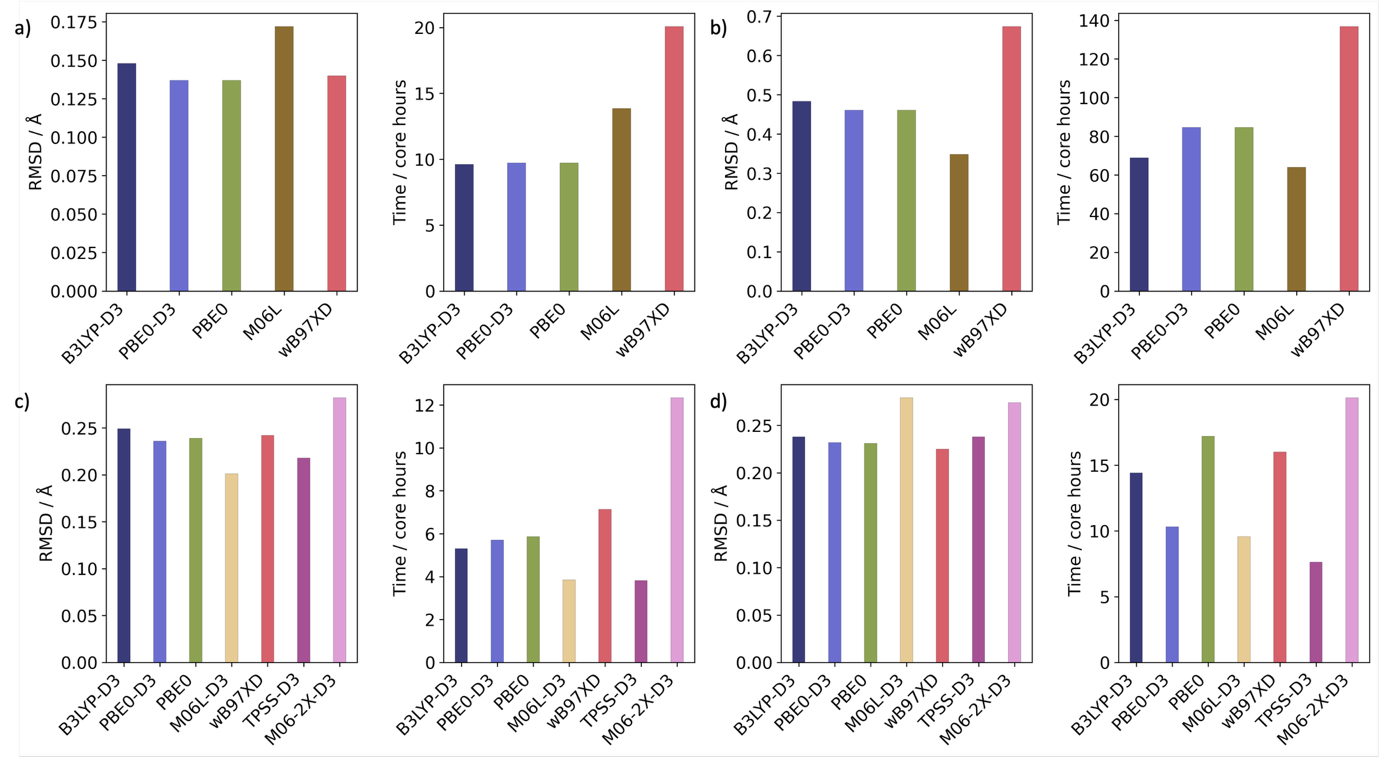


**Figure S5.** Comparison of DFT functionals in geometry optimization methods accuracy (RMSD against crystallographic structure) and computational cost (core-hours on 32 cores). a) complex c, b) complex d, c) complex e, and d) complex f.

To further benchmark the efficiency of quantum chemical calculations, we evaluated three different structure optimization processes using the simplified molecular input line entry system (SMILES) strings as the input of the complex. 1) DFT optimization using xyz structures generated directly from SMILES input. 2) xyz structures undergo a sequential workflow consisting of universal force field (UFF)^15^ optimization in Open Babel^16^, GFN2-xTB^17,18^ method optimization with GBSA solvation model, and finally DFT optimization using the chosen functional within in PCM solvent model. 3) xyz structures undergo sequential optimization with a universal force field, followed by GFN2-xTB optimization with GBSA solvation, then B97-3c functional in PCM solvent, and finally the DFT functional in a PCM solvent model. In this benchmark, we additionally tested several DFT functionals guided by the results obtained from the pure-DFT evaluation.

For the two Mo catalysts, B3LYP-D3, PBE0-D4 and wB97XD displayed the lowest values of the parameters. This aligns well with previously reported studies^27^. It is worth noting that the triplet state generally shows a lower RMSD value. The difference in accuracy between the three methods is about 1%, while their difference in computational time is significant. The PBE0-D4 method is faster, and its accuracy is also good for the monomer Co complex, as reported in Figure S6. Attempts to further reduce computational costs by using r2-SCAN-3c instead of B97-3c or by performing a direct PBE0-D4 optimization starting from the GFN2-xTB optimized structure, yield poor results. For Cr complex, PBE0-D3 showed the best balance of computational cost and accuracy, with the RMSD of 0.280 with respect to the crystal structures and with 16.93 h. It outperformed the direct DFT approach by offering faster calculations and improved convergence, without a significant loss of structural fidelity. For complex d, M06L produced the most accurate structure with the RMSD of 0.458 and CPU time of 115.68 h. PBE0-D3 achieved an RMSD of 0.680 and CPU time of 67.47 h. Overall, PBE0-D3 and M06L are the most suitable functionals for catalysts c and d, respectively. Therefore, the chosen pipeline of methods is: UFF, GFN2-xTB with GBSA solvent, B97-3c with CPCM solvent and PBE0-D4/def2-SVP with CPCM solvation, in a balance between structural accuracy and computational cost in geometry optimization.


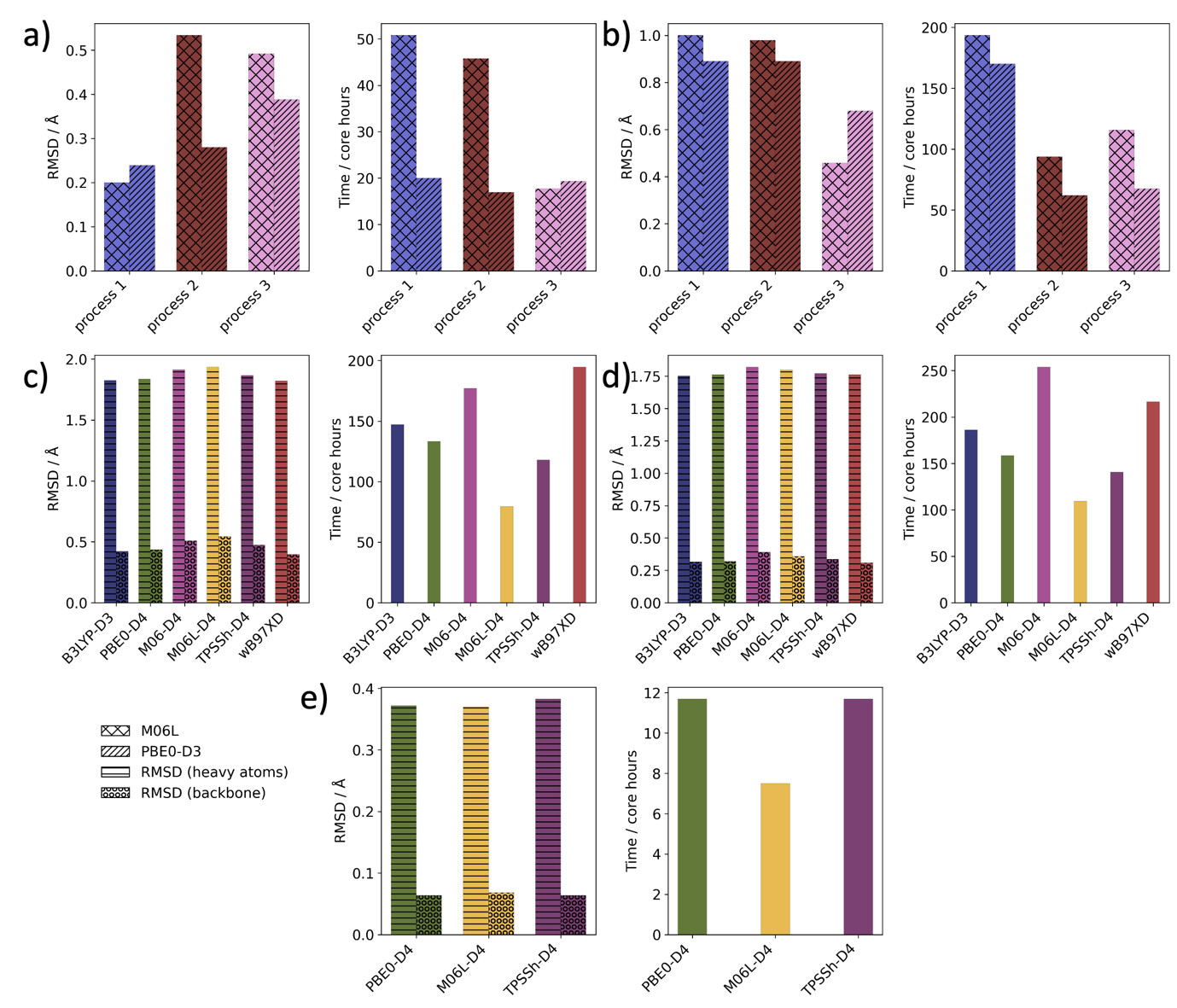


**Figure S6.** Comparison of DFT functionals and workflow processes in geometry optimization methods accuracy (RMSD against crystallographic structure) and computational cost (core-hours on 32 cores). a) complex c, b) complex d, c) complex a with singlet multiplicity, d) complex a with triplet multiplicity, and e) complex b.

With this solid computational benchmark, every structure was subsequently subjected to an optimization workflow composed of up to 10000 steps of UFF minimisation in OpenBabel, followed by a GFN2-xTB^10,11^ with GBSA solvent and frequency calculation to assess the obtainment of a minimum structure. Then, the geometry was further optimized at the B97-3c^12^ level of theory, and a final refinement was done at the PBE0-D4/def2-SVP level^13–15^. The full workflow was performed only for the lowest possible spin multiplicity, while evaluation of the metal oxidation state allowed for the identification of other possible spin multiplicities in a Python script, and for each of them, a geometry optimization was carried out using the lower state optimized structure as a starting guess. When a structure was used in different solvents, the whole workflow was carried out for one of them, and the optimized structure was used to converge a PBE0-D4/def2-SVP optimization in the other solvents. Finally, on every optimized structure, a single point calculation was carried out at the PBE0/def2-TZVPD level of theory. All the DFT calculations have been performed in ORCA 6.0.1^19,20^ using the CPCM solvation model.

## Redox potential and pK_a_

As an important driving force in the reaction, redox potentials play a critical role. The computed redox potentials are derived from DFT-calculated Gibbs free energies, referenced to the ferrocene/ferrocenium (Fc/Fc⁺) electrode (Equation 1). To be able to compare the computed redox potential to the experimental references, the effect of the solvent and the reference electrode used in the measurements needs to be accounted for. From the experimental studies reported by Nishibayashi’s group, the ferrocene/ferrocenium couple (Fc/Fc^+^) was used as the reference electrode in all cases.

$E_{R/O}^{\circ}=-\frac{\Delta G_{R/O}^{\circ}}{n_{e}F}-E_{abs}^{\circ}\left( Fc/Fc^{+} \right)$ **1**

An absolute redox potential in THF was not available in the literature. Therefore, an extensive evaluation of the Fc/Fc^+^ absolute redox potential in THF solution was carried out by means of DFT calculations. To estimate the redox potential in the best possible way, several DFT functionals, in both SMD and CPCM solvation models, have been applied. The evaluated DFT functionals include M06, PBE0, TPSSh, B3LYP, M06-2X and wB97XD, with empirical dispersion D4 and def2-TZVPD basis set. The structures of ferrocene and ferrocenium were optimized at the CPCM-PBE0-D4/def2-TZVPD level. The redox potential was estimated from the free energy difference between a complex and its oxidized/reduced form by calculating the gas phase ionization potential and then adding the solvation contribution for the two species, according to the following equation (Equation 2).

$E_{Fc/Fc^{+}}^{\circ}=-\frac{\Delta G_{Fc/Fc^{+}}^{gas}+\Delta G_{Fc_{\left( g \right)}-Fc_{\left( s \right)}}-\Delta G_{Fc_{\left( g \right)}^{+}-Fc_{\left( s \right)}^{+}}}{n_{e}F}$ **2**

All the calculated ferrocene absolute redox potentials are reported in Table S3. Except for M06, the values obtained by the other functionals are very consistent with each other. The contrast between the M06 and M06-2X can be indicative of the need to properly account for dispersion interactions, which is herein done with the empirical dispersion D4. The SMD solvation models lead to potentials that are always about 0.1 V smaller than the CPCM calculated ones. Specifically, the average values for the redox potential in the SMD solvation model are 4.694 V, while the CPCM scheme generates an average of up to 4.79 V. The values are consistent with previous computational and experimental results in different solvents^21,22^. As the following calculations have been carried out in the CPCM solvation model, 4.79 V was taken as the absolute redox potential for the Fc/Fc+ reference electrode in THF.

**Table S3.** Absolute redox potential of Fc/Fc^+^ calculated with different DFT functionals. All calculations used the def2-TZVPD basis set on a geometry optimized at the CPCM-PBE0/def2-TZVPD level.

| Method | Absolute potential (V) | |
| --- | --- | --- |
|  | CPCM solvation | SMD solvation |
| PBE0-D4 | 4.713 | 4.628 |
| B3LYP-D3 | 4.656 | 4.555 |
| TPSSh-D4 | 4.811 | 4.726 |
| M06-D4 | 5.120 | 5.023 |
| M06-2X | 4.685 | 4.577 |
| wB97XD | 4.755 | 4.656 |
| Average value | 4.790 | 4.694 |

To confirm the possibility of using the PBE0-D4/def2-TZVPD method for single-point calculation, its ability to reproduce experimental redox potential was assessed. A subset of six molecules was chosen (Figure S7), and the results are reported in Table S4, alongside the literature experimental values. The redox potentials were referenced to the Fc/Fc^+^ potentials previously calculated. The values, calculated with Equation 1, are close to the experimental references, with an average deviation of -0.098 V. Hence, the method is reliable for electronic energy calculations for NRR molecular catalysts. The free energy was determined by thermochemical calculation at the PBE0-D4/def2-TZVPD//PBE0-D4/def2-SVP level of theory in the CPCM solvation model. These computed values and methods are then used to calculate all redox potentials for the reductants in the compiled catalytic systems in their relevant solvent, as all reductants, solvents, and acids were considered in the datasets for training machine learning models.

**Table S4.** PBE0-D4/def2-TZVPD // PBE0-D4/def2-SVP level. All the values are referenced against the Fc/Fc^+^ electrode.

| Molecule | Calculated E°_R/O_ (V) | Reference value (V) | Deviation (V) |
| --- | --- | --- | --- |
| Mo29 | 0.367 | 0.11 ^23^ | -0.257 |
| Fe01 | -0.94 | -0.9 ^24^ | 0.040 |
| Mo30 | -1.09 | -1.14 ^25^ | -0.052 |
| Mo28 | -0.87 | -0.99 ^26^ | -0.117 |
| Mo34 | -0.99 | -1.18 ^25^ | -0.188 |
| Co01 | -0.58 | -0.60 ^2^ | -0.016 |

**Figure S7.** Structures of catalyst employed in the redox potential benchmark: (a) Mo29, (b) Fe01, (c) Mo30, (d) Mo28, (e) Mo34 and (f) Co01 catalysts.

Additionally, the same optimization and single-point methods were used to calculate the acid pKa in the relevant solvent applied in the nitrogen reduction reactions. The calculated pKa values for 18 acids: [H_2_NPh_2_]^+^, [3,5-LutH]^+^, [2,4,6-ColH]^+^, [2-PicH]^+^, HOCH_2_CH_2_OH, CH_3_OH, CH_3_CH_2_OH, *i*PrOH, *t*BuOH, CF_3_CH_2_OH, PhOH, [*t*Bu_3_PH]^+^, [Cy_3_PH]^+^, [*n*Bu_3_PH]^+^, [PyH]^+^, [N-Me-H_2_NPh]^+^, [H_3_NPh]^+^, [H_3_N-2,5-Cl_2_C_6_H_3_]^+^ show excellent correlation with experimentally reported data, reaching an R^2^ score of 0.98 (Figure S8). Therefore, this method was applied to estimate the pKa values of all other acids in their corresponding solvents where no experimental measurements are available.


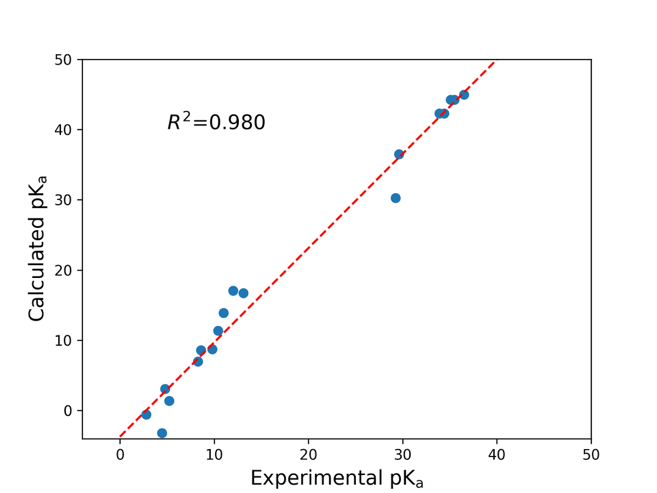


**Figure S8.** Correlation between experimental and calculated pKa of 18 acids.

# Chemical properties calculation and analysis

## Data extraction

**Table S5.** List of all descriptors extracted by the developed workflow, classified according to the property type. In the descriptors nomenclature, M stands for the metal atom, while X and Y are generic atoms in the first coordination sphere of the metal atom. The abbreviation, if any, used in the plots and for discussion of each feature is written in parentheses.

| Property type | Descriptors |
| --- | --- |
| Geometrical | M-X bond lengths (D1-D6), X-M-Y bond angles (BA1-BA6),  Wiberg bond indices (BO1-BO6) |
| Non-covalent interactions | Agostic interactions |
| Steric | Buried volume percentage (Buried_Vol) |
| Electronic, molecular level | Total (SP_Energy) and repulsion energy, dipole and quadrupole moments, HOMO/LUMO energies and HOMO-LUMO gap (HL_Gap) |
| Electronic, atomic level | Mulliken charge (MC0-MC6) and spin (MS0-MS6), Hirshfield charge (HC0-HC6) and spin (HS0-HS6), d electrons population on the metal from Löwdin orbital reduced pop analysis (Low_d_ele), number of metal d electrons by crystal field theory prediction (CFT_d_ele) |

The tested approach was utilised for chemical properties calculations, which will be fed into ML models for training and testing. The features have been chosen to explicitly sample the first coordination sphere of the metal (Table S5). The first coordination sphere was assumed to be formed by the six closest atoms to the metal centre, except for Family 1 complexes, where the carbon atoms connected to the bottom atom have also been included. The coordination spheres are highlighted in Figure S9.

The HOMO-LUMO gap and the Mulliken Analysis function have two different forms, corresponding to unrestricted (UHF, spin multiplicity > 1) or restricted (RHF, spin multiplicity = 1) calculations. In the UHF approach, the HOMO-LUMO gap is computed as the smallest energy difference between the highest occupied and lowest unoccupied orbitals, without considering if they have the same or opposite spin state (α or β). For the Mulliken analysis, the difference is that only the UHF calculation presents spin population data. In both charge and spin population analysis (Mulliken and Hirshfeld), the values are extracted only for the metal (indicated as 0) and for the six closest atoms to it, as shown in Figure S9, which are numbered 1 to 6 by distance from the metal centre. The list of atoms to extract is calculated locally and if more than one metal centre is present (e.g., in OBD topology), the process is repeated for each metal. The bond lengths are termed D1 to D6, ordered by distance between the ligand atom and the metal centre. The bond angles are listed BA1 to BA6 and are formed by the metal centre and pairs of atoms of the first coordination sphere, ordered by distance. The Wiberg bond orders (BO1-BO6) are listed by their value, following the quantum chemical calculation output order. At the end, the extracted data are compiled and exported to a CSV file.

**Figure S9.** Coordination sphere for each family of complexes, in particular, the considered atoms are highlighted in red.

## Property analysis


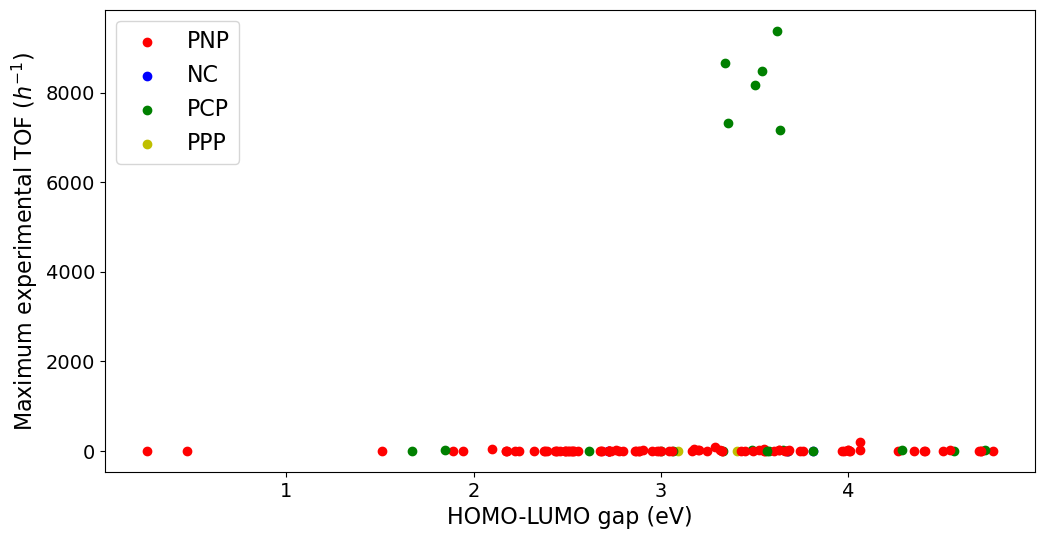


**Figure S10.** The distribution of HOMO–LUMO gaps versus the maximum experimental TOF. It shows an extremely weak correlation, likely due to the wide range of TOF values among these catalysts. Most catalysts are located around a TOF of 100 h^-1^, while a small group composed exclusively of PCP complexes stands out from the rest. This subset consists of six molecules with very similar structures, each containing three chlorines (Figure S11). Interestingly, the unsubstituted tridentate has the higher TOF.


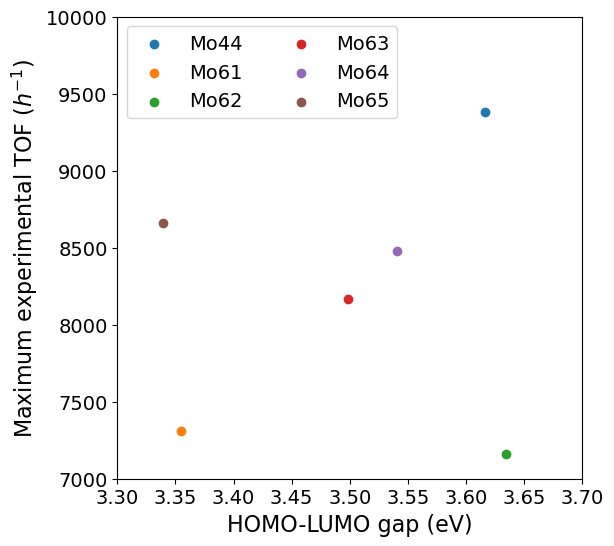

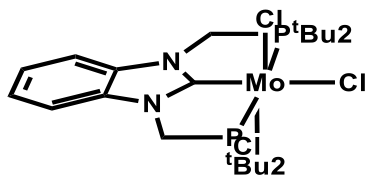

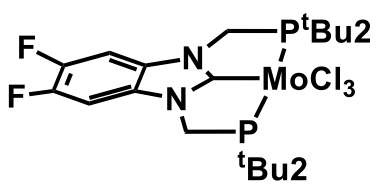

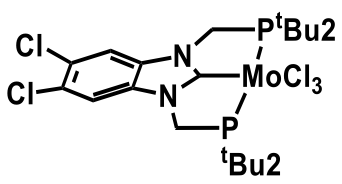

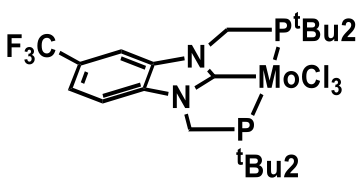

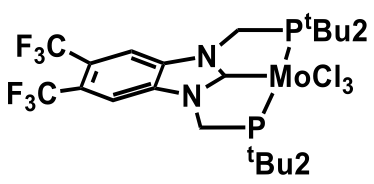

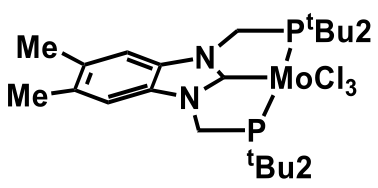


**Figure S11.** The distribution of HOMO–LUMO gaps versus the maximum experimental TOF of the six molecules with PCP ligands.


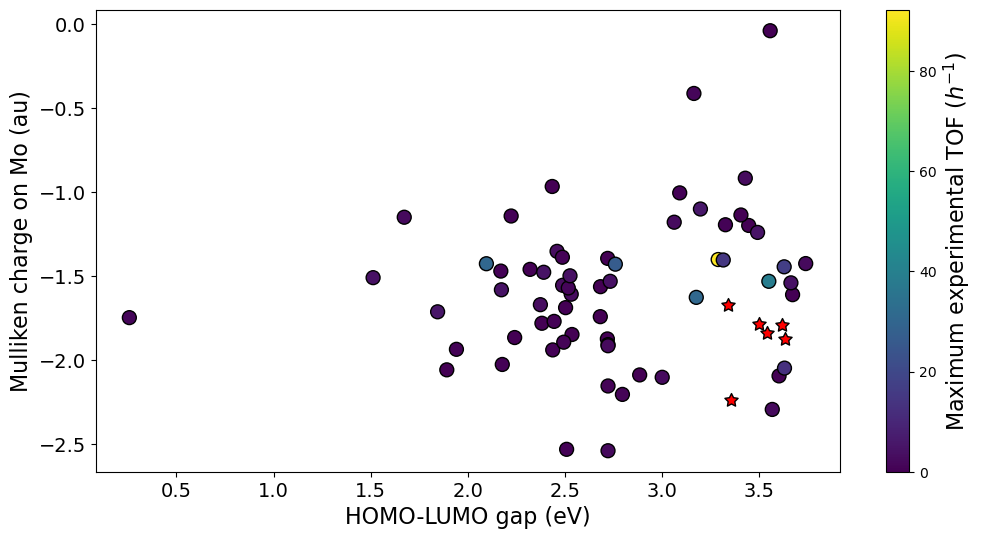


**Figure S12.** The scatter plot of HOMO–LUMO gap versus Mulliken charge on the Mo atom for all Mo complexes. The six complexes with “out-of-scale” TOF values (discussed previously) are highlighted as red stars, while the remaining points are colored according to their TOF. Almost all of the best-performing complexes, both the out-of-scale stars and the top performers within the scale, occupy the same region of the plot.

# Machine Learning Protocol

In order to determine the most efficient ML algorithm for the catalytic performance prediction of the metal-ligand catalysts, we included 27 regression models and classifiers, which are summarized in Table S6. Classification models have been focused on three indicators of catalytic performances: reactivity (TOF), stability (TON) and selectivity towards NRR (TON $\text{ratio}=\frac{\text{TON}\left( NH_{3} \right)}{\text{TON}\left( NH_{3} \right)+\text{TON}\left( H_{2} \right)}$ ). Regressor models have been employed to predict the values of TOF and TON of NRR.

We evaluated several train-test splitting possibilities in the initial phases, ranging from 50:50 to 90:10 on a small subset of ML models for TOF regression. As shown in Figure S13, the impact of the split ratio on both the train and test R^2^ significantly varies between different models. However, the highest scoring ones have improved training and test performance with an 80:20 ratio. Thus, we selected the 80:20 train-test split ratio for all machine learning studies we carried out.

**Table S6.** Classification and regression models applied in this study.

| Models class | Task | Included models |
| --- | --- | --- |
| Linear | Classification | Logistic Regression (LR) |
|  | Regression | Lasso CV (LC), Ridge CV (RC),  Bayesian Ridge (BR), ElasticNet CV (EN) |
| Nearest Neighbor | Classification | K-Nearest Neighbor (KNN) |
|  | Regression | K-Nearest Neighbor (KNN) |
| Support Vector Machine | Classification | Support Vector Classifier (SVC) |
| Naive Bayes | Classification | Gaussian Naive Bayes (GNB) |
| Gaussian Process | Regressor | Gaussian Process Regressor with standard kernel (GPR), Gaussian Process Regressor with Matern kernel (GPRMT), Gaussian Process Regressor with RBF kernel (GPRBF) |
| Tree and ensemble | Classification | Decision Tree (DT), Random Forest (RF), Extremely Randomized Trees (ET), Bagged Trees (BT), Gradient Boosting Decision Tree (GBDT), Adaptive Boosting (AB), Extreme Gradient Boosting (XGB) |
|  | Regressor | Decision Tree (DT), Random Forest (RF), Extremely Randomized Trees (ET), Bagged Trees (BT), Gradient Boosting Decision Tree (GBDT), Adaptive Boosting (AB), Extreme Gradient Boosting (XGB) |
| Neural Network | Regressor | Multi-layer Perceptron (MLP) |


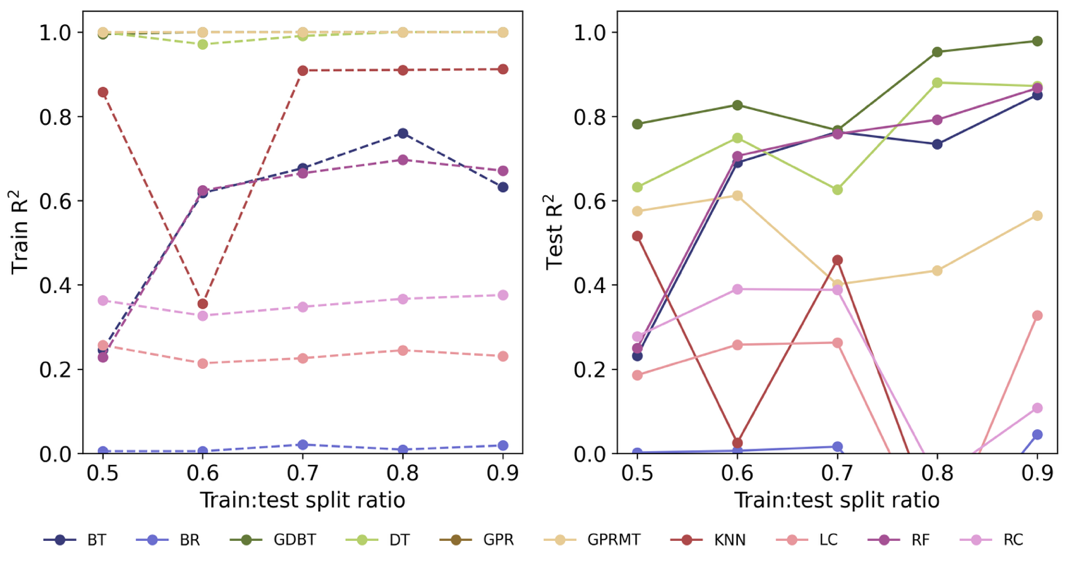


**Figure S13.** Effect of different train:test split ratios on the train and test R^2^ score for selected ML models.

Model performance was evaluated using several statistical metrics. For regressors, we reported R^2^, explained variance, MAE, MSE, and RMSE scores. For classifiers, we assessed accuracy, precision, recall and F_1_ scores. The explained variance represents the proportion of variance explained by independent variables, calculated as Equation 3. Similarly, the coefficient of determination R^2^ accounts for the explained variance as well as the residual between true and predicted values, which indicates the accuracy of the fit and its ability to predict unseen samples, as computed according to Equation 4. The mean absolute error (MAE) is a measure of errors between pairs of observations of the same phenomenon, as given by Equation 5. The mean squared error (MSE) is a risk metric given by the average of the squares of the errors (Equation 6), while RMSE (Equation 7) is the square root of MSE. In addition, for the regression models, we evaluated three relative error parameters, that are normalized over the scale of the target: relative MAE (RMAE, Equation 8), relative RMSE (RRMSE, Equation 9) and range-normalized RMSE (NRMSE, Equation 10). These complementary metrics provide an estimate of prediction error across datasets of different scales: RMAE accounts for all the errors equally, while RMSE-based metrics penalize larger deviations more strongly due to the squared term. In all cases, lower values indicate better performance. In particular, an RRMSE value below 50% indicates a good model performance, reflecting prediction errors that remain low relative to the variability of the target data.

For a classifier model, the accuracy expresses the fraction of correct predictions of the model and is computed using Equation 11. The precision (Equation 12) quantifies the ability of the classifier to correctly label the sample, while the recall (Equation 13) is a measure of the fraction of positive samples recovered. Finally, the F1 score, given by Equation 14, is the harmonic mean of the precision and recall metrics, and it is a measure of the predictive performance.

$\text{R}^{2}\left( y,\hat{y} \right)=1-\frac{\sum_{i=1}^{n} \left( y_{i}-\hat{y_{i}} \right)^{2}}{\sum_{i=1}^{n} \left( y_{i}-\bar{y} \right)^{2}}$ **3**

where $\bar{y}=\frac{1}{n}\sum_{i=1}^{n} y_{i}$ , $\sum_{i=1}^{n} \left( y_{i}-\hat{y_{i}} \right)^{2}=\sum_{i=1}^{n} \epsilon_{i}^{2}$ , $y$ is the true value, $\hat{y}$ is the predicted value, $n$ is the number of samples and $\epsilon$ is the error between true and predicted value.

$\text{explained variance}\left( y,\hat{y} \right)=1-\frac{Var\{y-\hat{y}\}}{Var\{y\}}$ **4**

$\text{MAE}\left( y,\hat{y} \right)=\frac{1}{n}\sum_{i=0}^{n-1} \left| y_{i}-\hat{y_{i}} \right|$ **5**

$\text{MSE}\left( y,\hat{y} \right)=\frac{1}{n}\sum_{i=0}^{n-1} \left( y_{i}-\hat{y_{i}} \right)^{2}$ **6**

$\text{RMSE}\left( y,\hat{y} \right)=\sqrt{\text{MSE}\left( y,\hat{y} \right)}=\sqrt{\frac{1}{n}\sum_{i=0}^{n-1} \left( y_{i}-\hat{y_{i}} \right)^{2}}$ **7**

$\text{RM}\text{AE}\left( y,\hat{y} \right)=\frac{MAE\left( y,\hat{y} \right)}{\bar{y}}$ **8**

$\text{RR}\text{MSE}\left( y,\hat{y} \right)=\frac{RMSE}{\sigma(y)}$ **9**

$\text{NR}\text{MSE}\left( y,\hat{y} \right)=\frac{RMSE}{y_{max}-y_{min}}$ **10**

$\text{accuracy}\left( y,\hat{y} \right)=\frac{1}{n}\sum_{i=0}^{n-1} 1\left( \hat{y_{i}}=y_{i} \right)$ **11**

$\text{precision}=\frac{tp}{tp+fp}$ **12**

$\text{recall}=\frac{tp}{tp+fn}$ **13**

$\text{F}_{\text{1}}=2\frac{\text{precision}\times\text{recall}}{\text{precision}+\text{recall}}=\frac{2tp}{2tp+fp+fn}$ **14**

where $tp$ stands for “true positives”, $fp$ for “false positives” and $fn$ for “false negatives”.

For classification tasks, the synthetic minority oversampling technique (SMOTE) method was used for data enhancement, considering the initial class imbalance of the dataset. Its core idea is to generate new synthetic samples for the minority classes by interpolating minority-class samples, thereby alleviating the model bias caused by the class imbalance. Specifically, it identifies the k-nearest neighbours of each minority-class instance and generates new samples along the line segments connecting the point to its neighbours. This augmentation enhances the representation of minority classes during training and helps improve the generalization ability of the models.

For all models, the hyperparameters of each algorithmic model were optimized by grid search with nested 10-fold cross-validation (CV) to prevent training overfitting.

Shapley Additive Explanations (SHAP)^27^ analysis was conducted on the trained ML models. SHAP stands out as one of the widely accepted methods for elucidating ML models. In this approach, each feature is assigned an importance scale, where a higher absolute SHAP value denotes a more substantial contribution to the outcomes of ML models. Moreover, a positive or negative SHAP value signifies that the feature exerts a positive or negative effect on the prediction. Additionally, partial dependence plots (PDP) have been calculated for the regression models. The PDP represents the relationship between the target and a feature value, allowing for complementing the SHAP analysis by identifying if this relationship is linear, monotonic or a more complex function. Thus, it can provide a quantitative estimation of the optimal range of a feature.

# Additional results for family 1 (Peters’) catalysts

## Feature analysis and selection


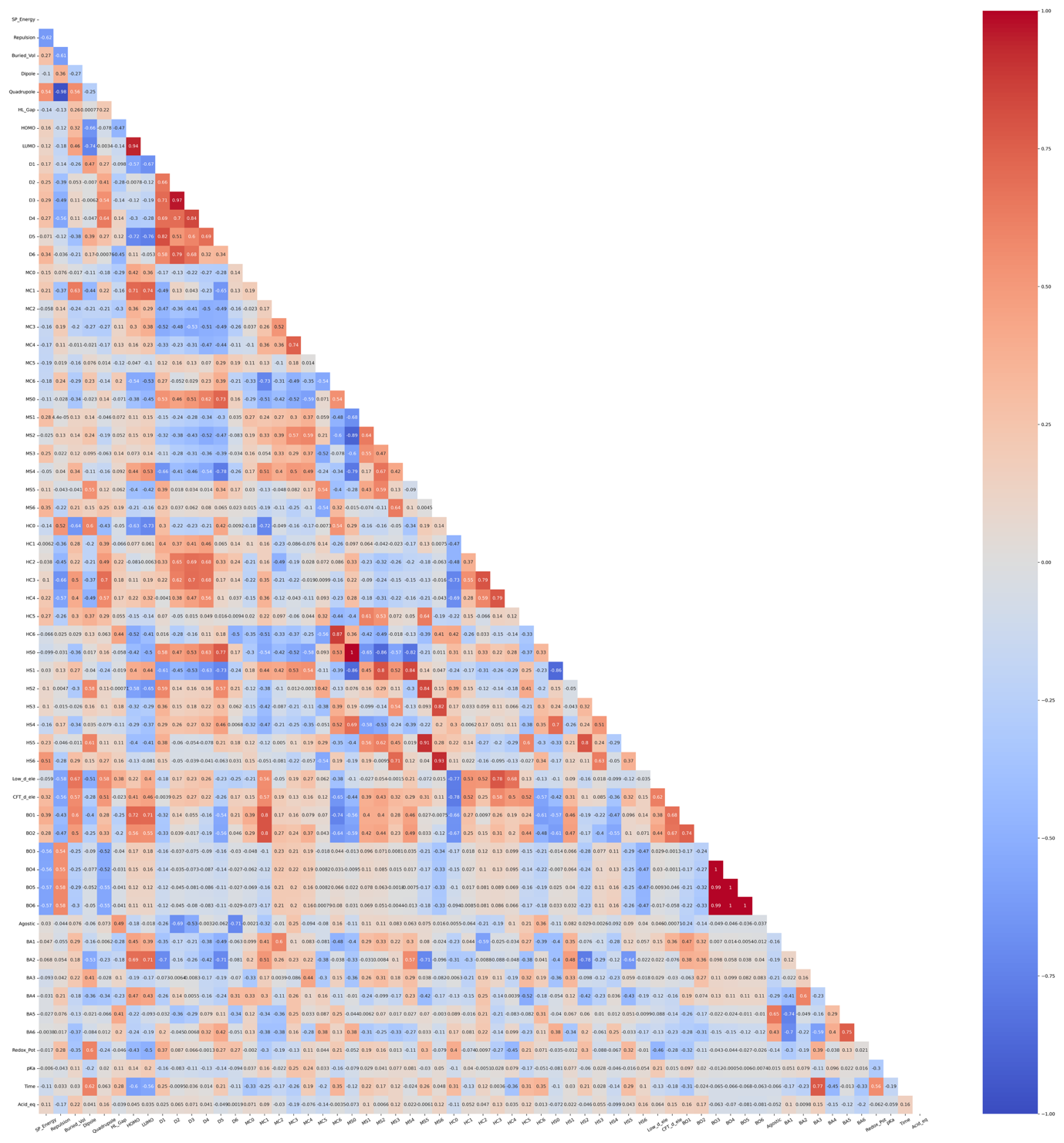


**Figure S14.** Pearson correlation coefficient matrix for features in family 1 catalyst dataset.

## Classification models for family 1 (Peters’) catalysts

The training of all classification models for family 1 catalysts was carried out using 15 features: SP Energy, Repulsion, HOMO-LUMO gap, HC1, HC2, HC3, HC4, HC5, HC6, HS0, BA4, BO1, reductant redox potential, acid pKa and acid equivalents. The optimized hyperparameters for the selected models are listed in Table S7Table S16.

**Table S7.** Hyperparameters of the best ML model for family 1 catalysts.

| Target | Model | Best hyperparameters |
| --- | --- | --- |
| Reactivity | RF | max_depth=32, max_features='log2', min_samples_leaf=2, min_samples_split=4,  n_estimators=112, random_state=4659 |
| Stability | AB | learning_rate=2.0, n_estimators=135, random_state=4659 |
| TOF | ET | max_depth = 5, n_estimators=15 |
|  | MLP | Activation=’relu’, hidden_layer_size=(24,12,), max_iter=10000, solver=lbfgs |
| TON | AB | learning_rate=0.02, loss='square', n_estimators= 30 |
|  | MLP | Activation=’relu’, hidden_layer_size=(50,25), max_iter=10000, solver=lbfgs |

**Table S8.** Accuracy of the train and test sets was enhanced with SMOTE of trained classification models for family 1 catalysts.

| Reactivity | | | Stability | | |
| --- | --- | --- | --- | --- | --- |
| Model | Train accuracy | Test accuracy | Model | Train accuracy | Test accuracy |
| RF | 1.0 | 1.0 | AB | 1.000 | 1.000 |
| ET | 0.992 | 1.0 | GDBT | 1.000 | 1.000 |
| KNN | 1.0 | 1.0 | RF | 0.992 | 1.000 |
| GDBT | 1.0 | 1.0 | ET | 0.992 | 1.000 |
| AB | 1.0 | 1.0 | LR | 0.984 | 0.969 |
| DT | 0.992 | 1.0 | SVC | 0.984 | 0.969 |
| LR | 0.985 | 1.0 | KNN | 0.984 | 0.969 |
| SVC | 0.985 | 1.0 | DT | 0.977 | 0.969 |
| XGB | 0.969 | 1.0 | XGB | 0.977 | 0.938 |
| GNB | 0.824 | 0.788 | GNB | 0.719 | 0.750 |

| a)  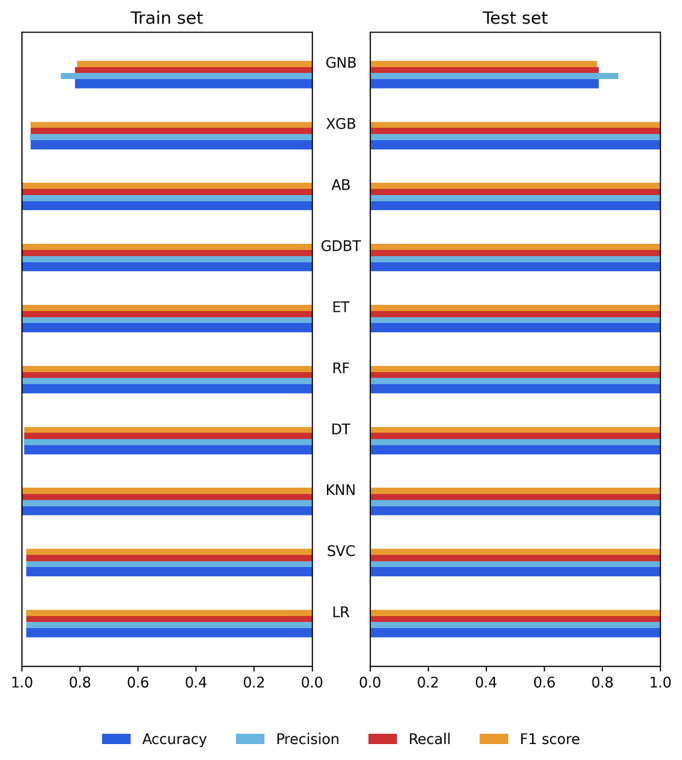 | b)  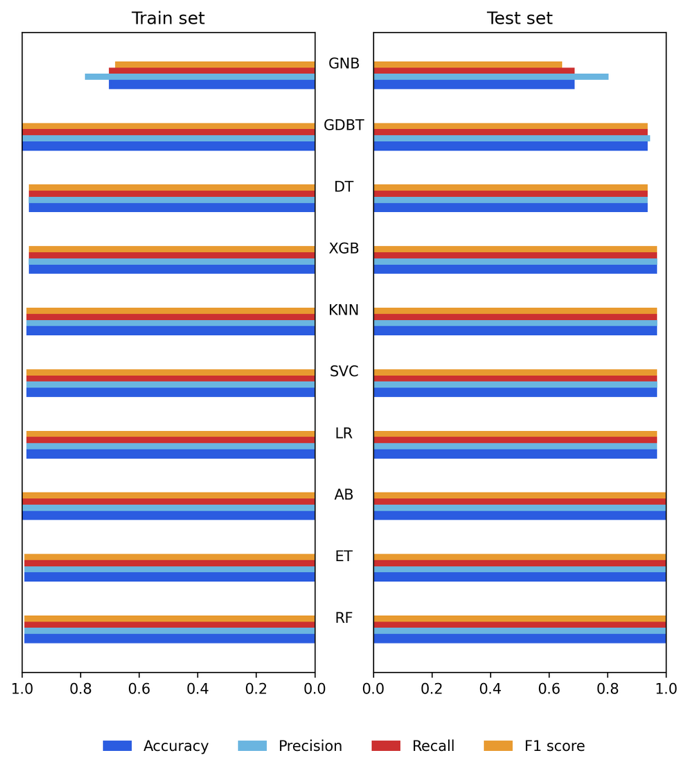 |
| --- | --- |

**Figure S15.** ML metrics classifiers models for (a) reactivity (TOF > 20 h^-1^) and (b) stability (TON > 20 cat^-1^) in family 1 dataset.

**Table S9.** Accuracy, Precision, Recall, and F1 Score of the Model in the train and Test Set test set enhanced with SMOTE of trained classification models for reactivity (TOF > 20 h^-1^) of family 1 catalysts.

| Model | Train Accuracy | Test Accuracy | Train Precision | Test Precision | Train Recall | Test Recall | Train F1 score | Test F1 score |
| --- | --- | --- | --- | --- | --- | --- | --- | --- |
| Linear model | | | | | | | | |
| LR | 0.985 | 1.000 | 0.985 | 1.000 | 0.985 | 1.000 | 0.985 | 1.000 |
| Support Vector Machine model | | | | | | | | |
| SVC | 0.985 | 1.000 | 0.985 | 1.000 | 0.985 | 1.000 | 0.985 | 1.000 |
| Nearest Neighbor model | | | | | | | | |
| KNN | 1.000 | 1.000 | 1.000 | 1.000 | 1.000 | 1.000 | 1.000 | 1.000 |
| Naive Bayes model | | | | | | | | |
| GNB | 0.824 | 0.788 | 0.869 | 0.855 | 0.824 | 0.788 | 0.818 | 0.782 |
| Tree and Ensemble models | | | | | | | | |
| DT | 0.992 | 1.000 | 0.992 | 1.000 | 0.992 | 1.000 | 0.992 | 1.000 |
| RF | 1.000 | 1.000 | 1.000 | 1.000 | 1.000 | 1.000 | 1.000 | 1.000 |
| ET | 0.992 | 1.000 | 0.992 | 1.000 | 0.992 | 1.000 | 0.992 | 1.000 |
| GDBT | 1.000 | 1.000 | 1.000 | 1.000 | 1.000 | 1.000 | 1.000 | 1.000 |
| AB | 1.000 | 1.000 | 1.000 | 1.000 | 1.000 | 1.000 | 1.000 | 1.000 |
| XGB | 0.969 | 1.000 | 0.971 | 1.000 | 0.969 | 1.000 | 0.969 | 1.000 |

**Table S10.** Accuracy, Precision, Recall, and F1 Score of the Model in the train and test set enhanced with SMOTE of trained classification models for stability (TON > 20 cat^-1^) of family 1 catalysts.

| Model | Train Accuracy | Test Accuracy | Train Precision | Test Precision | Train Recall | Test Recall | Train F1 score | Test F1 score |
| --- | --- | --- | --- | --- | --- | --- | --- | --- |
| Linear model | | | | | | | | |
| LR | 0.984 | 0.969 | 0.984 | 0.969 | 0.984 | 0.969 | 0.984 | 0.969 |
| Support Vector Machine model | | | | | | | | |
| SVC | 0.984 | 0.969 | 0.985 | 0.970 | 0.984 | 0.969 | 0.984 | 0.969 |
| Nearest Neighbor model | | | | | | | | |
| KNN | 0.984 | 0.969 | 0.984 | 0.970 | 0.984 | 0.969 | 0.984 | 0.969 |
| Naive Bayes model | | | | | | | | |
| GNB | 0.703 | 0.688 | 0.785 | 0.803 | 0.703 | 0.688 | 0.682 | 0.645 |
| Tree and Ensemble models | | | | | | | | |
| RF | 0.992 | 1.000 | 0.992 | 1.000 | 0.992 | 1.000 | 0.992 | 1.000 |
| ET | 0.992 | 1.000 | 0.992 | 1.000 | 0.992 | 1.000 | 0.992 | 1.000 |
| AB | 1.000 | 1.000 | 1.000 | 1.000 | 1.000 | 1.000 | 1.000 | 1.000 |
| XGB | 0.977 | 0.969 | 0.978 | 0.970 | 0.977 | 0.969 | 0.977 | 0.969 |
| DT | 0.977 | 0.938 | 0.978 | 0.938 | 0.977 | 0.938 | 0.977 | 0.938 |
| GBDT | 1.000 | 0.938 | 1.000 | 0.945 | 1.000 | 0.938 | 1.000 | 0.938 |

| a)  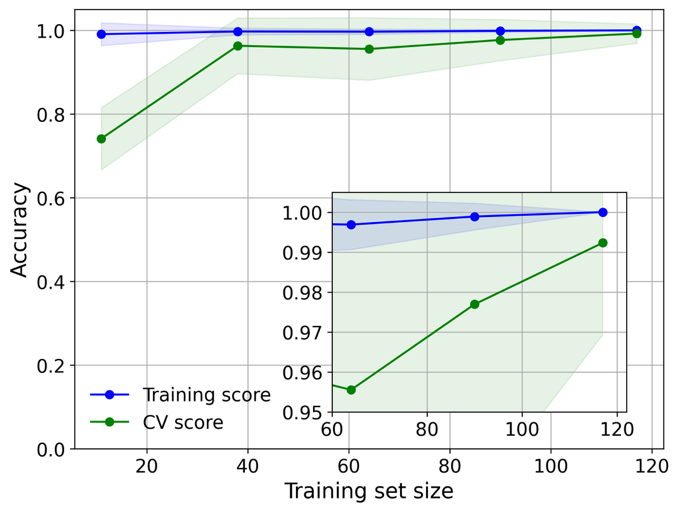 | b)  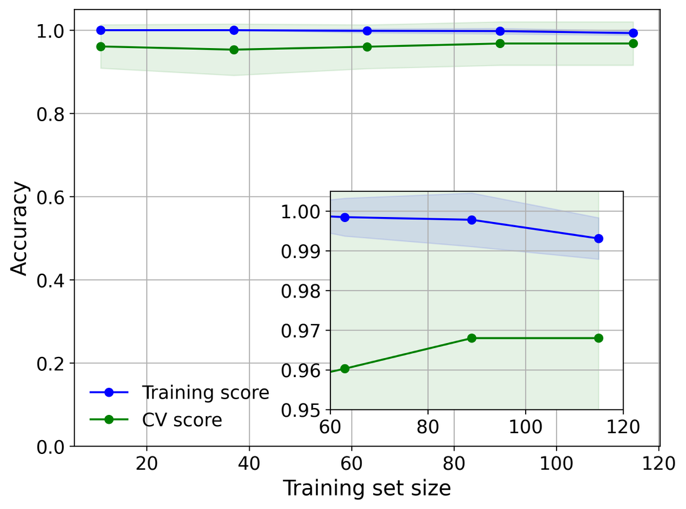 |
| --- | --- |

**Figure S16.** Learning curve (on training set) for the best performing ML classification models over family 1 catalysts dataset: a) reactivity (RF model) and (b) stability (AB model).

## Regression models for family 1 (Peters’) catalysts

The training of regression models for family 1 catalysts used two different sets of features for TOF and TON prediction. In particular, the TOF models used the following 15 features: SP Energy, Repulsion, HOMO-LUMO gap, HC1, HC2, HC3, HC4, HC5, HC6, HS0, BA4, BO1, reductant redox potential, acid pK_a_ and acid equivalents. Instead, the TON regression was performed on a slightly different set of 15 features: SP Energy, Repulsion, HC1, HC2, HC3, HC4, HC5, HC6, HS0, Löwdin d electrons, BA2, BA3, BA4, acid pK_a_ and acid equivalents.

We also explored several techniques to improve model performance, including logarithmic scaling (log10(x+1)) of target data and polynomial feature expansion, particularly for linear models. In family 1 catalysts, logarithmic scaling improved only the DT and ET models in TON prediction, where the test R^2^ goes from ~0.75-0.8 to 0.9. In contrast, polynomial expansion greatly improves linear models, raising the LC test R^2^ from 0.550 to 0.890 for TOF prediction, and from 0.581 to 0.886 for TON. While in family 2 catalysts, logarithmic scaling improved several linear models, increasing test R^2^ scores from ~0.3 to ~0.7, though several previously best-scoring tree and ensemble models exhibited slight decreases (Table S15, S16). However, back-conversion of predicted values to the original scale (y’=exp(y) – 1) didn’t improve ML models. Applying polynomial features to both original and log-scaled target data, followed by back-conversion, likewise failed to yield satisfactory improvements for linear models. These results indicate that polynomial features can capture only simple nonlinearities and are insufficient to model the complex chemical phenomena influenced by multiple experimental factors and a large range of targets. Consequently, their performance is still limited, especially compared to the much higher test accuracy achieved by the MLP model (Figure S17).

**Table S11.** Accuracy and relative error metrics of ML regression models on training and test prediction for TOF (h^-1^) of family 1 catalyst.

| Model | Train R^2^ | Test R^2^ | Train RMAE | Test RMAE | Train RRMSE | Test RRMSE | Train NRMSE | Test NRMSE |
| --- | --- | --- | --- | --- | --- | --- | --- | --- |
| ET | 0.981 | 0.900 | 0.2371 | 0.2949 | 0.1392 | 0.2959 | 0.0212 | 0.0793 |
| GPRBF | 0.972 | 0.894 | 0.2704 | 0.2949 | 0.1601 | 0.3182 | 0.0244 | 0.0853 |
| GPRMT | 0.974 | 0.894 | 0.2818 | 0.2804 | 0.1674 | 0.3173 | 0.0255 | 0.085 |
| MLP | 0.99 | 0.88 | 0.0650 | 0.4045 | 0.0705 | 0.3446 | 0.0107 | 0.0924 |
| AB | 0.995 | 0.797 | 0.0809 | 0.4926 | 0.1002 | 0.4398 | 0.0152 | 0.1179 |
| XGB | 0.944 | 0.752 | 0.3402 | 0.4799 | 0.2313 | 0.4868 | 0.0352 | 0.1305 |
| DT | 0.933 | 0.738 | 0.395 | 0.557 | 0.2556 | 0.6102 | 0.0389 | 0.1635 |
| GDBT | 0.993 | 0.732 | 0.8458 | 0.7509 | 0.6563 | 0.6304 | 0.0999 | 0.1689 |
| RC | 0.557 | 0.577 | 0.8917 | 0.7791 | 0.6671 | 0.6372 | 0.1015 | 0.1708 |
| EC | 0.548 | 0.576 | 0.8483 | 0.7435 | 0.6839 | 0.638 | 0.1041 | 0.171 |
| LC | 0.550 | 0.574 | 0.5504 | 0.7001 | 0.5579 | 0.6661 | 0.0849 | 0.1785 |
| BR | 0.527 | 0.573 | 0.7398 | 0.8574 | 0.731 | 0.74 | 0.1112 | 0.1983 |
| KNN | 0.685 | 0.534 | 1.2758 | 1.2452 | 0.9999 | 0.9778 | 0.1521 | 0.262 |
| RF | 0.491 | 0.466 | 1.1359 | 1.1394 | 0.9939 | 0.9868 | 0.1512 | 0.2645 |
| BT | -0.012 | -0.004 | 0.0809 | 0.4926 | 0.1002 | 0.4398 | 0.0152 | 0.1179 |
| GPR | 0.000 | -0.023 | 0.3402 | 0.4799 | 0.2313 | 0.4868 | 0.0352 | 0.1305 |

**Table S12.** Accuracy and relative error metrics of ML regression models on training and test prediction for TON (cat^-1^) of family 1 catalyst.

| Model | Train R^2^ | Test R^2^ | Train RMAE | Test RMAE | Train RRMSE | Test RRMSE | Train NRMSE | Test NRMSE |
| --- | --- | --- | --- | --- | --- | --- | --- | --- |
| AB | 0.900 | 0.880 | 0.1727 | 0.3093 | 0.3146 | 0.3385 | 0.0527 | 0.0907 |
| XGB | 0.879 | 0.879 | 0.3141 | 0.3459 | 0.3452 | 0.3395 | 0.0578 | 0.091 |
| MLP | 0.90 | 0.87 | 0.1755 | 0.3400 | 0.3123 | 0.3553 | 0.0523 | 0.0952 |
| GPRMT | 0.875 | 0.857 | 0.3239 | 0.3433 | 0.3509 | 0.3696 | 0.0587 | 0.099 |
| GPRBF | 0.865 | 0.848 | 0.345 | 0.3475 | 0.3657 | 0.3808 | 0.0612 | 0.102 |
| DT | 0.866 | 0.797 | 0.3526 | 0.4275 | 0.3634 | 0.4398 | 0.0608 | 0.1178 |
| GBDT | 0.882 | 0.643 | 0.2018 | 0.4647 | 0.3418 | 0.5835 | 0.0572 | 0.1563 |
| RC | 0.559 | 0.585 | 0.6829 | 0.5625 | 0.6723 | 0.6316 | 0.1125 | 0.1692 |
| LC | 0.542 | 0.581 | 0.6907 | 0.5706 | 0.6817 | 0.6348 | 0.1141 | 0.17 |
| EN | 0.542 | 0.581 | 0.6712 | 0.5695 | 0.6597 | 0.6287 | 0.1104 | 0.1684 |
| BR | 0.530 | 0.577 | 0.5655 | 0.6715 | 0.4918 | 0.6912 | 0.0823 | 0.1851 |
| ET | 0.755 | 0.498 | 0.5733 | 0.7255 | 0.6042 | 0.7237 | 0.1011 | 0.1939 |
| KNN | 0.631 | 0.450 | 0.6561 | 0.6863 | 0.7526 | 0.7241 | 0.126 | 0.1939 |
| RF | 0.427 | 0.450 | 1.1289 | 1.0161 | 0.9939 | 0.9879 | 0.1663 | 0.2646 |
| GPR | 0.000 | -0.025 | 1.0947 | 0.9946 | 0.9945 | 0.9932 | 0.1665 | 0.266 |
| BT | -0.001 | -0.036 | 0.3239 | 0.3433 | 0.3509 | 0.3696 | 0.0587 | 0.099 |

**Table S13.** All metrics for TOF regression models in family 1 complexes.

| Model | Train R^2^ | Test R^2^ | Train MAE | Test MAE | Train MSE | Test MSE | Train RMSE | Test RMSE | Train ex. variance | Test ex. variance |
| --- | --- | --- | --- | --- | --- | --- | --- | --- | --- | --- |
| Linear models | | | | | | | | | | |
| LC | 0.549 | 0.574 | 7.380 | 9.150 | 148.370 | 226.350 | 12.181 | 15.045 | 0.549 | 0.574 |
| EN | 0.548 | 0.575 | 7.330 | 9.050 | 148.710 | 225.370 | 12.195 | 15.012 | 0.548 | 0.575 |
| RC | 0.557 | 0.577 | 7.110 | 8.980 | 145.770 | 224.760 | 12.074 | 14.992 | 0.557 | 0.577 |
| BR | 0.526 | 0.573 | 7.020 | 8.730 | 155.940 | 226.890 | 12.487 | 15.063 | 0.526 | 0.573 |
| Nearest Neighbor models | | | | | | | | | | |
| KNN | 0.685 | 0.534 | 4.560 | 8.220 | 103.760 | 247.310 | 10.186 | 15.726 | 0.685 | 0.534 |
| Gaussian Process Regressor models | | | | | | | | | | |
| GPRMT | 0.974 | 0.894 | 2.240 | 3.460 | 8.550 | 56.440 | 2.924 | 7.513 | 0.974 | 0.894 |
| GPRBF | 0.972 | 0.894 | 2.330 | 3.290 | 9.340 | 56.110 | 3.056 | 7.491 | 0.972 | 0.894 |
| GPR | 0.000 | -0.023 | 9.410 | 13.380 | 329.330 | 542.840 | 18.147 | 23.299 | 0.000 | 0.000 |
| Tree and ensemble models | | | | | | | | | | |
| ET | 0.981 | 0.900 | 1.930 | 3.790 | 6.200 | 53.030 | 2.490 | 7.282 | 0.981 | 0.900 |
| DT | 0.933 | 0.738 | 2.960 | 5.720 | 22.190 | 138.910 | 4.711 | 11.786 | 0.933 | 0.738 |
| XGB | 0.944 | 0.752 | 2.430 | 5.730 | 18.400 | 131.520 | 4.290 | 11.468 | 0.944 | 0.752 |
| AB | 0.994 | 0.797 | 0.480 | 5.860 | 1.830 | 107.980 | 1.353 | 10.391 | 0.994 | 0.797 |
| GBDT | 0.994 | 0.848 | 0.470 | 6.460 | 2.240 | 142.540 | 1.497 | 11.939 | 0.994 | 0.848 |
| BT | -0.01 | -0.004 | 10.560 | 14.620 | 333.350 | 532.970 | 18.258 | 23.086 | 0.000 | 0.000 |
| RF | 0.491 | 0.466 | 5.710 | 9.190 | 178.140 | 305.220 | 12.950 | 16.831 | 0.491 | 0.466 |
| Neural network | | | | | | | | | | |
| MLP | 0.99 | 0.88 | 0.54 | 4.75 | 1.66 | 66.21 | 1.29 | 8.14 | 0.99 | 0.88 |

**Table S14.** All metrics for TON regression models in family 1 complexes.

| Model | Train R^2^ | Test R^2^ | Train MAE | Test MAE | Train MSE | Test MSE | Train RMSE | Test RMSE | Train ex. variance | Test ex. variance |
| --- | --- | --- | --- | --- | --- | --- | --- | --- | --- | --- |
| Linear models | | | | | | | | | | |
| RC | 0.559 | 0.585 | 7.310 | 8.270 | 175.550 | 220.120 | 13.250 | 14.836 | 0.559 | 0.585 |
| LC | 0.542 | 0.581 | 7.440 | 8.170 | 182.340 | 222.190 | 13.503 | 14.906 | 0.542 | 0.581 |
| EN | 0.542 | 0.581 | 7.440 | 8.170 | 182.340 | 222.190 | 13.503 | 14.906 | 0.542 | 0.581 |
| BR | 0.530 | 0.577 | 7.530 | 8.290 | 187.470 | 224.420 | 13.692 | 14.981 | 0.530 | 0.577 |
| Nearest Neighbor models | | | | | | | | | | |
| KNN | 0.631 | 0.450 | 6.250 | 10.540 | 147.240 | 291.670 | 12.134 | 17.078 | 0.635 | 0.465 |
| Gaussian Process Regressor models | | | | | | | | | | |
| GPRMT | 0.875 | 0.857 | 3.530 | 4.990 | 49.660 | 76.070 | 7.047 | 8.722 | 0.875 | 0.859 |
| GPRBF | 0.865 | 0.848 | 3.760 | 5.050 | 53.960 | 80.770 | 7.346 | 8.987 | 0.865 | 0.851 |
| GPR | 0.000 | -0.025 | 12.30 | 14.760 | 398.460 | 543.550 | 19.962 | 23.314 | 0.000 | 0.000 |
| Tree and ensemble models | | | | | | | | | | |
| ET | 0.755 | 0.498 | 6.160 | 9.750 | 97.560 | 266.050 | 9.877 | 16.311 | 0.755 | 0.512 |
| DT | 0.866 | 0.797 | 3.840 | 6.210 | 53.260 | 107.710 | 7.298 | 10.378 | 0.866 | 0.807 |
| XGB | 0.879 | 0.879 | 3.420 | 5.020 | 48.060 | 64.210 | 6.933 | 8.013 | 0.879 | 0.882 |
| AB | 0.900 | 0.880 | 1.880 | 4.490 | 39.930 | 63.820 | 6.319 | 7.989 | 0.900 | 0.881 |
| GBDT | 0.882 | 0.643 | 2.200 | 6.750 | 47.130 | 189.610 | 6.865 | 13.770 | 0.883 | 0.683 |
| BT | -0.001 | -0.036 | 11.93 | 14.440 | 398.990 | 549.370 | 19.975 | 23.439 | 0.000 | 0.000 |
| RF | 0.427 | 0.450 | 7.150 | 9.970 | 228.460 | 291.960 | 15.115 | 17.087 | 0.427 | 0.465 |
| Neural network | | | | | | | | | | |
| MLP | 0.90 | 0.87 | 1.91 | 4.94 | 39.33 | 70.29 | 6.27 | 8.38 | 0.90 | 0.87 |

**Table S15.** Metrics for the model trained on data scaled by log10(x+1) for family 1 catalysts dataset. The "scaled" label identifies the metrics obtained with prediction on a logarithmic scale, while the "back-conv" indicates the corresponding prediction when the predicted values (y) are converted back to the original scale (y’=exp(y) - 1). For comparison, the accuracy of the models for the original/unscaled data is also reported.

| Model name | TOF original | | TOF scaled | | TOF back-conv | | TON original | | TON back-conv | | TON back-conv | |
| --- | --- | --- | --- | --- | --- | --- | --- | --- | --- | --- | --- | --- |
|  | Train R^2^ | Test R^2^ | Train R^2^ | Test R^2^ | Train R^2^ | Test R^2^ | Train R^2^ | Test R^2^ | Train R^2^ | Test R^2^ | Train R^2^ | Test R^2^ |
| Linear models | | | | | | | | | | | | |
| LC | 0.550 | 0.574 | 0.563 | 0.520 | 0.413 | 0.615 | 0.542 | 0.581 | 0.488 | 0.588 | 0.419 | 0.312 |
| RC | 0.564 | 0.583 | 0.562 | 0.515 | 0.367 | 0.533 | 0.559 | 0.585 | 0.565 | 0.731 | 0.518 | 0.739 |
| BR | 0.527 | 0.573 | 0.559 | 0.515 | 0.358 | 0.516 | 0.530 | 0.577 | 0.489 | 0.578 | 0.417 | 0.292 |
| EN | 0.548 | 0.576 | 0.550 | 0.522 | 0.362 | 0.522 | 0.542 | 0.581 | 0.502 | 0.610 | 0.449 | 0.378 |
| Nearest Neighbor models | | | | | | | | | | | | |
| KNN | 0.685 | 0.534 | 0.809 | 0.371 | 0.862 | 0.785 | 0.631 | 0.450 | 0.633 | 0.696 | 0.532 | 0.331 |
| Gaussian Process Regressor models | | | | | | | | | | | | |
| GPR | 0.000 | -0.023 | 0.000 | -0.007 | -0.084 | -0.143 | 0.000 | -0.025 | 0.000 | -0.006 | -0.117 | -0.257 |
| GPRBF | 0.972 | 0.894 | 0.956 | 0.288 | 0.994 | 0.696 | 0.865 | 0.848 | 0.882 | 0.802 | 0.832 | 0.783 |
| GPRMT | 0.974 | 0.894 | 0.956 | 0.390 | 0.994 | 0.733 | 0.875 | 0.857 | 0.889 | 0.797 | 0.832 | 0.783 |
| Tree and ensemble models | | | | | | | | | | | | |
| DT | 0.934 | 0.609 | 0.753 | 0.578 | 0.867 | 0.207 | 0.866 | 0.797 | 0.7586 | 0.8739 | 0.8758 | 0.9011 |
| ET | 0.980 | 0.908 | 0.885 | 0.709 | 0.882 | 0.818 | 0.755 | 0.498 | 0.8213 | 0.9013 | 0.8724 | 0.9003 |
| GBDT | 0.994 | 0.848 | 0.816 | 0.467 | 0.488 | 0.287 | 0.882 | 0.643 | 0.7864 | 0.7999 | 0.4383 | 0.3905 |
| XGB | 0.946 | 0.751 | 0.873 | 0.706 | 0.902 | 0.750 | 0.879 | 0.879 | 0.7990 | 0.883 | 0.615 | 0.622 |
| AB | 0.990 | 0.797 | 0.954 | 0.518 | 0.995 | 0.796 | 0.900 | 0.880 | 0.9057 | 0.902 | 0.873 | 0.902 |
| BT | -0.012 | -0.004 | 0.695 | 0.552 | 0.454 | 0.440 | -0.001 | -0.036 | 0.6172 | 0.618 | 0.248 | 0.063 |
| RF | 0.459 | 0.425 | 0.683 | 0.572 | 0.419 | 0.366 | 0.427 | 0.450 | 0.5278 | 0.557 | 0.112 | -0.038 |

**Table S16**. Metrics for linear models trained on polynomial features generated from the standardized data of family 1 dataset. In the TOF poly, the original target values were used, while in TOF poly back-conv, the targets were initially scaled to log10(x+1) and then converted back to the original scale.

| Model name | TOF poly | | TOF poly  back-conv | | TON poly | | TON poly  back-conv | |
| --- | --- | --- | --- | --- | --- | --- | --- | --- |
|  | Train R^2^ | Test R^2^ | Train R^2^ | Test R^2^ | Train R^2^ | Test R^2^ | Train R^2^ | Test R^2^ |
| LC | 0.971 | 0.890 | 0.867 | 0.758 | 0.868 | 0.886 | 0.790 | 0.816 |
| RC | 0.930 | 0.830 | 0.820 | 0.757 | 0.836 | 0.836 | 0.726 | 0.779 |
| BR | 0.975 | 0.868 | 0.383 | 0.298 | 0.845 | 0.847 | 0.392 | 0.389 |
| EN | 0.968 | 0.883 | 0.758 | 0.671 | 0.867 | 0.883 | 0.822 | 0.850 |

| a)  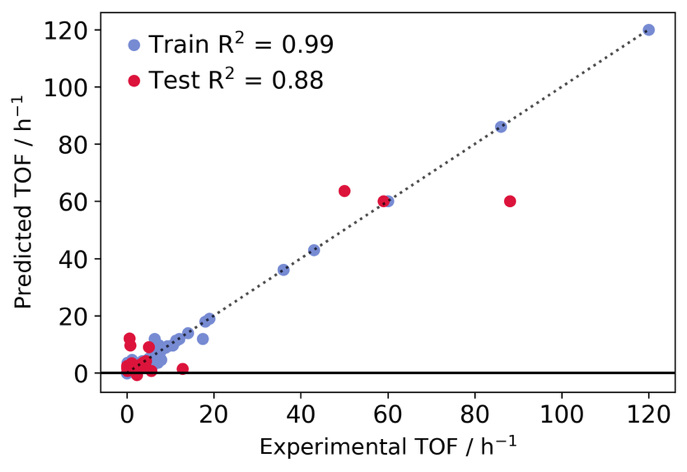 | b) 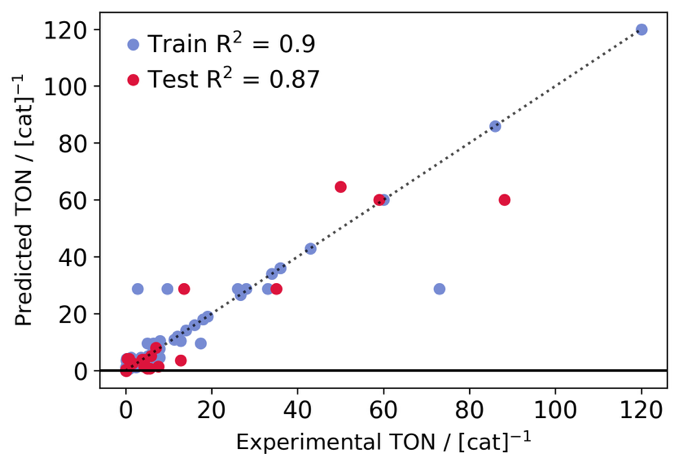 |
| --- | --- |

**Figure S17**. Accuracy plots of MLP models for (a) TOF and (b) TON for the family 1 dataset.

## Regression of Family 1 dataset with Family 2 features list

**Table S17**. Accuracy of ML regression models on training and test prediction for TOF (h-1) and TON (cat-1) of family 1 catalysts using family 2 features set.

| TOF | | | TON | | |
| --- | --- | --- | --- | --- | --- |
| Model | Train R^2^ | Test R^2^ | Model | Train R^2^ | Test R^2^ |
| AB | 0.4234 | 0.3306 | AB | 0.4370 | 0.3008 |
| GPRBF | 0.3804 | 0.2822 | KNN | 0.3924 | 0.2864 |
| GPRMT | 0.3778 | 0.2805 | GDBT | 0.3298 | 0.2385 |
| KNN | 0.1058 | 0.2502 | XGB | 0.2682 | 0.1952 |
| RC | 0.0972 | 0.0970 | RC | 0.1072 | 0.0575 |
| EC | 0.0920 | 0.0862 | DT | 0.1535 | 0.0568 |
| LC | 0.0926 | 0.0861 | LC | 0.0759 | 0.0197 |
| GDBT | 0.1029 | 0.0633 | EC | 0.0759 | 0.0197 |
| ET | 0.0671 | 0.0291 | ET | 0.0680 | 0.0152 |
| BR | 0.0566 | 0.0267 | BR | 0.0392 | 0.0117 |
| XGB | 0.0605 | 0.0260 | GPRBF | 0.0170 | -0.0071 |
| DT | 0.0327 | 0.0218 | GPRMT | 0.0168 | -0.0073 |
| BT | -0.0041 | -0.0100 | GPR | -0.0000 | -0.0248 |
| GPR | -0.0000 | -0.0226 | BT | -0.0003 | -0.0300 |
| RF | 0.0000 | -0.1129 | RF | 0.0000 | -0.1047 |

## Additional SHAP and PDP analysis on family 1 (Peters’) catalysts

| a)  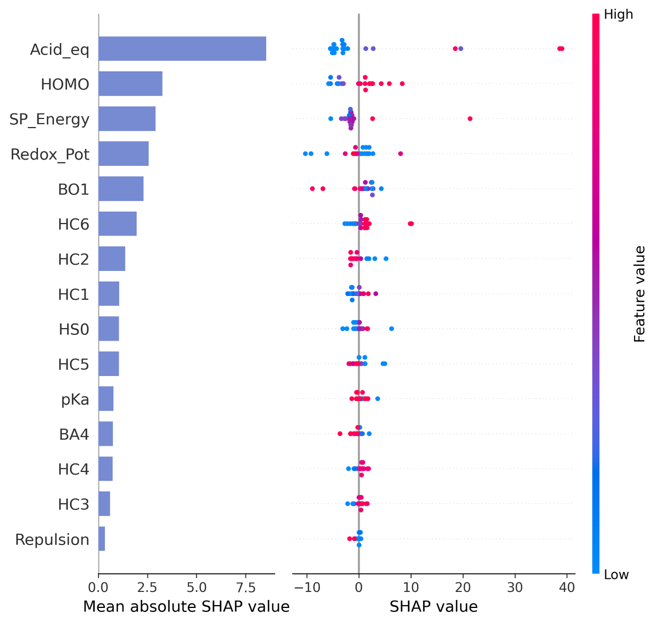 | b)  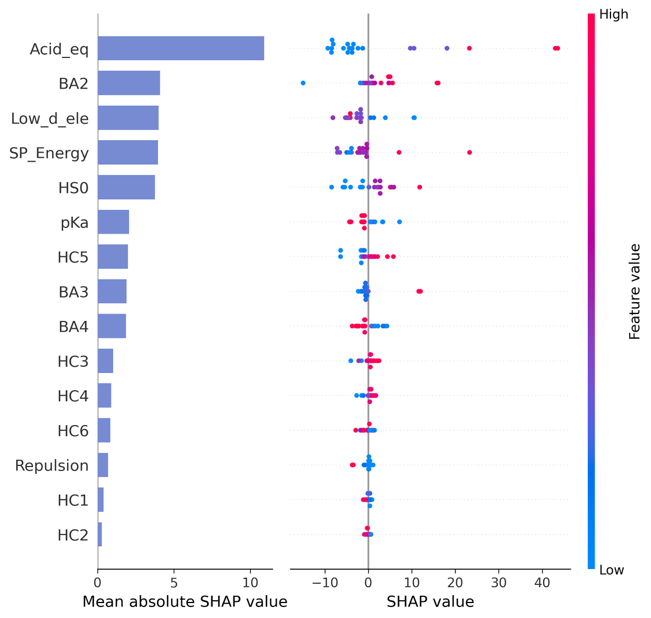 |
| --- | --- |

**Figure S18.** Feature importance and impact on the predicted value by SHAP analysis of the trained MLP models for family 1 dataset for (a) TOF and (b) TON prediction. The features are ordered by importance in the model.


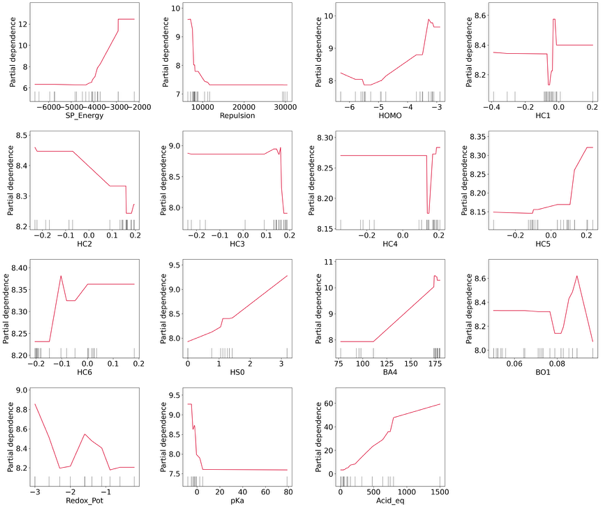


**Figure S19.** Partial dependence plots (PDP) for TOF (ET model) ML regression models for family 1 dataset. The partial dependence was calculated between the (0.05 and 0.95) percentile and it is shown in red, while the dataset values of each feature are displayed in grey.


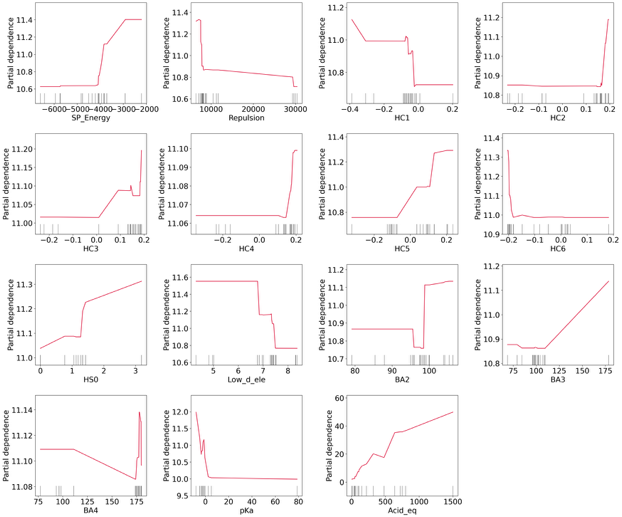


**Figure S20.** Partial dependence plots (PDP) for TON (AB model) ML regression models for family 1 dataset. The partial dependence was calculated between the (0.05 and 0.95) percentile and it is shown in red, while the dataset values of each feature are displayed in gray.

# Additional results for family 2 (Nishibayashi’s) catalysts

## Feature analysis and selection

After performing the DFT calculations and compiling the experimental conditions, a total of 62 features are obtained for each catalytic system. They include the computational descriptors listed in Table S5, the calculated redox potential of the reductant and pKa of the acid, experimental acid and reductant equivalents, and the reaction time. To identify the most informative feature subset for each dataset, we applied principal component analysis (PCA) and correlation analysis (Pearson and Spearman’s).


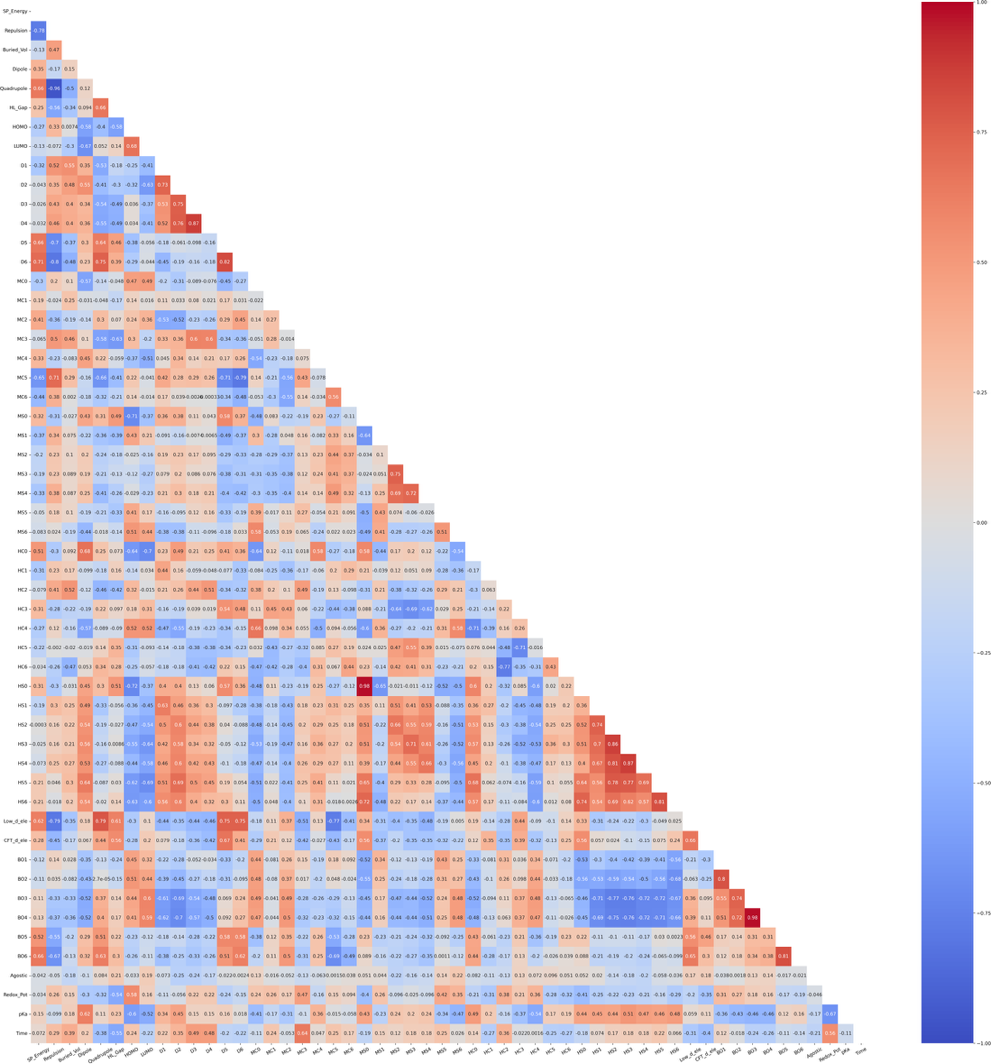


**Figure S21.** Spearman’s rank correlation matrix between the features in family 2 catalyst dataset.

## Classification models for family 2 (Nishibayashi’s) catalysts

The training of all classification models for family catalysts was carried out using these 16 features: SP Energy, repulsion, quadrupole, HOMO-LUMO gap, HC1, HC2, HC3, HC4, HC5, HC6, HS0, Löwdin d electrons, Agostic, reductant redox potential, acid pKa and reaction time. The optimized hyperparameters for the selected models are listed in Table S18.

**Table S18.** Hyperparameters of the best ML model for family 2 catalysts

| Target | Model | Best hyperparameters |
| --- | --- | --- |
| Reactivity | DT | max_depth=4, max_features='log2', min_samples_split=4, random_state=4659 |
| Stability | AB | learning_rate=1.5, n_estimators=145, random_state=4659 |
| Selectivity | RF | max_depth=28, min_samples_leaf=3, min_samples_split=7,  n_estimators=85, random_state=4659 |
| TOF | ET | max_depth = 9, min_samples_leaf = 1, min_samples_split = 2,  n_estimators = 200, random_state=42 |
|  | MLP | activation=’relu’, hidden_layer_sizes=(40,), max_iter=100000, solver= lbfgs |
| TON | GBDT | alpha=0.9, learning_rate = 0.3, max_depth = None, max_features = None,  min_samples_leaf = 2, n_estimators = 200, subsample = 0.8 |
|  | MLP | activation=’relu’, hidden_layer_sizes=(40,20,), max_iter=100000, solver= lbfgs |

**Table S19.** Accuracy of train and test set enhanced with SMOTE for trained classification models for family 2 catalysts’ reactivity, stability and selectivity.

| Reactivity | | | Stability | | | Selectivity | | |
| --- | --- | --- | --- | --- | --- | --- | --- | --- |
| Model | Train accuracy | Test accuracy | Model | Train accuracy | Test accuracy | Model | Train accuracy | Test accuracy |
| DT | 0.995 | 1.000 | AB | 0.990 | 0.962 | RF | 0.919 | 0.824 |
| GBDT | 0.996 | 1.000 | SVC | 0.969 | 0.955 | KNN | 0.909 | 0.824 |
| KNN | 0.991 | 0.993 | KNN | 0.981 | 0.955 | GBDT | 0.911 | 0.824 |
| GNB | 0.973 | 0.993 | RF | 0.979 | 0.955 | ET | 0.887 | 0.794 |
| RF | 0.996 | 0.993 | ET | 0.983 | 0.955 | AB | 0.877 | 0.794 |
| ET | 0.982 | 0.993 | GBDT | 0.992 | 0.955 | XGB | 0.874 | 0.794 |
| AB | 0.996 | 0.993 | XGB | 0.977 | 0.955 | DT | 0.800 | 0.775 |
| XGB | 0.991 | 0.993 | LR | 0.910 | 0.924 | SVC | 0.764 | 0.745 |
| SVC | 0.977 | 0.979 | DT | 0.977 | 0.924 | LR | 0.722 | 0.716 |
| LR | 0.948 | 0.950 | GNB | 0.914 | 0.909 | GNB | 0.571 | 0.578 |

| a)  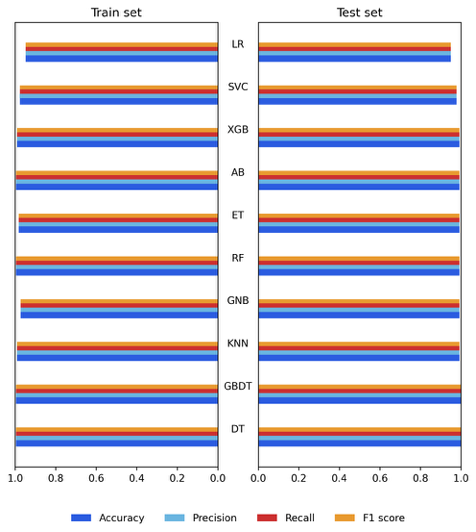 | b)  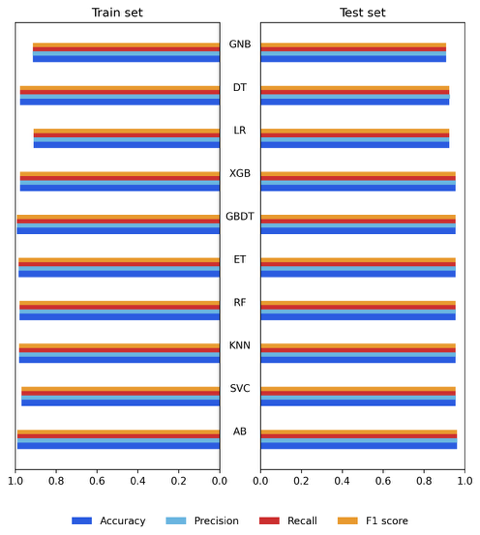 | c)  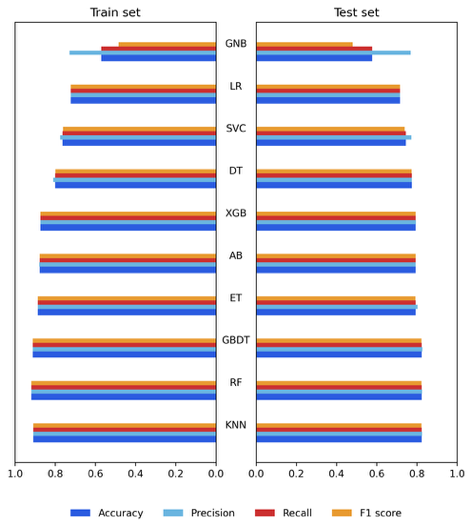 |
| --- | --- | --- |

**Figure S22.** ML metrics classifiers models for (a) reactivity, (b) stability, and (c) selectivity in family 2 dataset.

**Table S20.** Accuracy, Precision, Recall, and F1 Score of the model in the train and test set enhanced with SMOTE of trained classification models for reactivity (TOF > 200 h^-1^) of family 2 catalysts.

| Model | Train Accuracy | Test Accuracy | Train Precision | Test Precision | Train Recall | Test Recall | Train F1 score | Test F1 score |
| --- | --- | --- | --- | --- | --- | --- | --- | --- |
| Linear model | | | | | | | | |
| LR | 0.948 | 0.950 | 0.948 | 0.950 | 0.948 | 0.950 | 0.948 | 0.950 |
| Support Vector Machine model | | | | | | | | |
| SVC | 0.977 | 0.979 | 0.978 | 0.979 | 0.977 | 0.979 | 0.977 | 0.979 |
| Nearest Neighbor model | | | | | | | | |
| KNN | 0.991 | 0.993 | 0.991 | 0.993 | 0.991 | 0.993 | 0.991 | 0.993 |
| Naive Bayes model | | | | | | | | |
| GNB | 0.973 | 0.993 | 0.973 | 0.993 | 0.973 | 0.993 | 0.973 | 0.993 |
| Tree and Ensemble models | | | | | | | | |
| RF | 0.996 | 0.993 | 0.996 | 0.993 | 0.996 | 0.993 | 0.996 | 0.993 |
| ET | 0.982 | 0.993 | 0.982 | 0.993 | 0.982 | 0.993 | 0.982 | 0.993 |
| AB | 0.996 | 0.993 | 0.996 | 0.993 | 0.996 | 0.993 | 0.996 | 0.993 |
| XGB | 0.991 | 0.993 | 0.991 | 0.993 | 0.991 | 0.993 | 0.991 | 0.993 |
| DT | 0.995 | 1.000 | 0.995 | 1.000 | 0.995 | 1.000 | 0.995 | 1.000 |
| GBDT | 0.996 | 1.000 | 0.996 | 1.000 | 0.996 | 1.000 | 0.996 | 1.000 |

**Table S21.** Accuracy, Precision, Recall, and F1 Score of the model in the train and test set enhanced with SMOTE of trained classification models for stability (TON > 200 cat^-1^) of family 2 catalysts.

| Model | Train Accuracy | Test Accuracy | Train Precision | Test Precision | Train Recall | Test Recall | Train F1 score | Test F1 score |
| --- | --- | --- | --- | --- | --- | --- | --- | --- |
| Linear model | | | | | | | | |
| LR | 0.910 | 0.924 | 0.910 | 0.924 | 0.910 | 0.924 | 0.910 | 0.924 |
| Support Vector Machine model | | | | | | | | |
| SVC | 0.969 | 0.955 | 0.970 | 0.956 | 0.969 | 0.955 | 0.969 | 0.955 |
| Nearest Neighbor model | | | | | | | | |
| KNN | 0.981 | 0.955 | 0.981 | 0.956 | 0.981 | 0.955 | 0.981 | 0.955 |
| Naive Bayes model | | | | | | | | |
| GNB | 0.914 | 0.909 | 0.915 | 0.909 | 0.914 | 0.909 | 0.914 | 0.909 |
| Tree and Ensemble models | | | | | | | | |
| RF | 0.979 | 0.955 | 0.979 | 0.956 | 0.979 | 0.955 | 0.979 | 0.955 |
| ET | 0.983 | 0.955 | 0.983 | 0.956 | 0.983 | 0.955 | 0.983 | 0.955 |
| GDBT | 0.992 | 0.955 | 0.992 | 0.956 | 0.992 | 0.955 | 0.992 | 0.955 |
| XGB | 0.977 | 0.955 | 0.977 | 0.956 | 0.977 | 0.955 | 0.977 | 0.955 |
| DT | 0.977 | 0.924 | 0.977 | 0.928 | 0.977 | 0.924 | 0.977 | 0.924 |
| AB | 0.990 | 0.962 | 0.990 | 0.963 | 0.990 | 0.962 | 0.990 | 0.962 |

**Table S22.** Accuracy, Precision, Recall, and F1 Score of the model in the train and test set, enhanced with SMOTE of trained classification models for selectivity (TON ratio > 0.75) of family 2 catalysts.

| Model | Train Accuracy | Test Accuracy | Train Precision | Test Precision | Train Recall | Test Recall | Train F1 score | Test F1 score |
| --- | --- | --- | --- | --- | --- | --- | --- | --- |
| Linear model | | | | | | | | |
| LR | 0.722 | 0.716 | 0.722 | 0.716 | 0.722 | 0.716 | 0.722 | 0.716 |
| Support Vector Machine model | | | | | | | | |
| SVC | 0.764 | 0.745 | 0.776 | 0.773 | 0.764 | 0.745 | 0.761 | 0.740 |
| Nearest Neighbor model | | | | | | | | |
| KNN | 0.909 | 0.824 | 0.909 | 0.826 | 0.909 | 0.824 | 0.909 | 0.823 |
| Naive Bayes model | | | | | | | | |
| GNB | 0.571 | 0.578 | 0.728 | 0.769 | 0.571 | 0.578 | 0.484 | 0.481 |
| Tree and Ensemble models | | | | | | | | |
| RF | 0.919 | 0.824 | 0.919 | 0.826 | 0.919 | 0.824 | 0.919 | 0.823 |
| GBDT | 0.911 | 0.824 | 0.912 | 0.828 | 0.911 | 0.824 | 0.911 | 0.823 |
| ET | 0.887 | 0.794 | 0.887 | 0.804 | 0.887 | 0.794 | 0.887 | 0.793 |
| AB | 0.877 | 0.794 | 0.879 | 0.794 | 0.877 | 0.794 | 0.877 | 0.794 |
| XGB | 0.874 | 0.794 | 0.874 | 0.795 | 0.874 | 0.794 | 0.874 | 0.794 |
| DT | 0.800 | 0.775 | 0.810 | 0.775 | 0.800 | 0.775 | 0.799 | 0.774 |

| a)  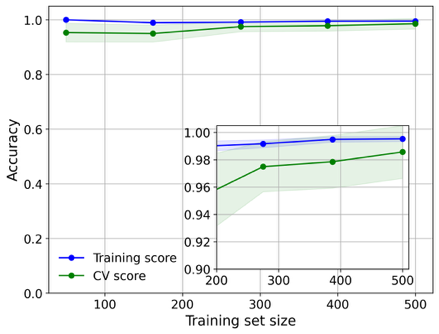 | b)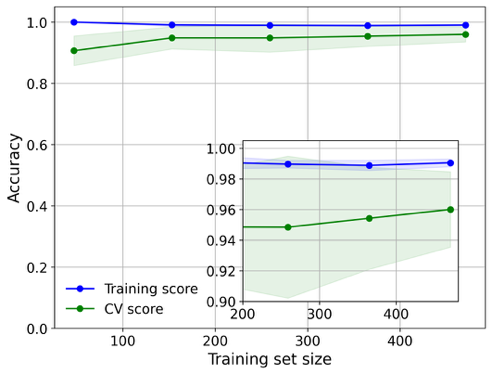 | c) 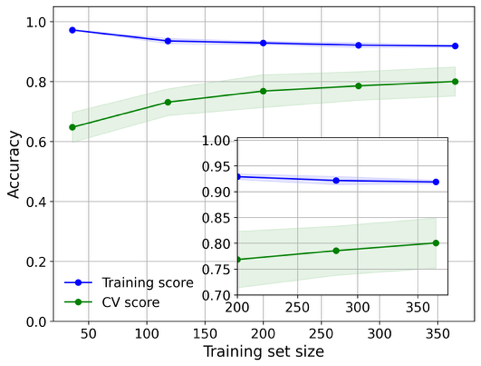 |
| --- | --- | --- |

**Figure S23.** Learning curve (on training set) for the best performing ML classification models over family 2 catalyst dataset: a) reactivity (DT model), b) stability (AB model), and c) selectivity (RF model).

## Regression models for family 2 (Nishibayashi’s) catalysts

The training of all regression models for family 2 catalyst was carried out using the same set of 16 features: SP Energy, repulsion, quadrupole, HOMO-LUMO gap, HC1, HC2, HC3, HC4, HC5, HC6, HS0, Löwdin d electrons, Agostic, reductant redox potential, acid pKa and reaction time. The same dataset was used in the MLP for TOF, while in the feature list of MLP for TON the MS0 (Mulliken spin population on the metal) replaced HS0 and the molecular dipole moment, D1 and D2 bond distances have been included too.

**Table S23.** Accuracy and relative error metrics of ML regression models on training and test prediction for TOF (h^-1^) and TON (cat^-1^) of family 2 catalyst.

| Model | Train R^2^ | Test R^2^ | Train RMAE | Test RMAE | Train RRMSE | Test RRMSE | Train NRMSE | Test NRMSE |
| --- | --- | --- | --- | --- | --- | --- | --- | --- |
| MLP | 1.00 | 0.97 | 0.105 | 0.082 | 0.108 | 0.061 | 0.013 | 0.008 |
| ET | 0.997 | 0.962 | 0.048 | 0.178 | 0.058 | 0.193 | 0.007 | 0.027 |
| DT | 1.000 | 0.954 | 0.006 | 0.201 | 0.008 | 0.212 | 0.001 | 0.029 |
| XGB | 0.965 | 0.871 | 0.268 | 0.512 | 0.188 | 0.357 | 0.022 | 0.049 |
| AB | 1.000 | 0.821 | 0.006 | 0.388 | 0.008 | 0.421 | 0.001 | 0.058 |
| GPRMT | 0.991 | 0.749 | 0.130 | 0.982 | 0.094 | 0.498 | 0.011 | 0.068 |
| GPRBF | 0.987 | 0.747 | 0.150 | 1.043 | 0.113 | 0.500 | 0.013 | 0.069 |
| KNN | 0.899 | 0.692 | 0.367 | 0.634 | 0.317 | 0.551 | 0.038 | 0.076 |
| GBDT | 1.000 | 0.657 | 0.006 | 0.517 | 0.008 | 0.582 | 0.001 | 0.080 |
| BT | 0.630 | 0.580 | 0.788 | 0.911 | 0.608 | 0.644 | 0.072 | 0.088 |
| RF | 0.620 | 0.544 | 0.742 | 0.915 | 0.615 | 0.671 | 0.073 | 0.092 |
| LC | 0.372 | 0.348 | 1.638 | 1.659 | 0.791 | 0.803 | 0.094 | 0.110 |
| EN | 0.370 | 0.347 | 1.657 | 1.700 | 0.798 | 0.812 | 0.095 | 0.111 |
| RC | 0.361 | 0.332 | 1.762 | 1.914 | 0.993 | 0.991 | 0.118 | 0.136 |
| BR | 0.011 | 0.004 | 1.781 | 1.907 | 0.998 | 0.994 | 0.119 | 0.136 |
| GPR | 0.000 | 0.000 | 0.048 | 0.178 | 0.058 | 0.193 | 0.007 | 0.027 |

**Table S24.** Accuracy and relative error metrics of ML regression models on training and test prediction for TON (cat^-1^) of family 2 catalyst.

| Model | Train R^2^ | Test R^2^ | Train RMAE | Test RMAE | Train RRMSE | Test RRMSE | Train NRMSE | Test NRMSE |
| --- | --- | --- | --- | --- | --- | --- | --- | --- |
| GBDT | 0.996 | 0.991 | 0.054 | 0.102 | 0.062 | 0.093 | 0.004 | 0.024 |
| AB | 0.996 | 0.981 | 0.050 | 0.142 | 0.062 | 0.137 | 0.004 | 0.036 |
| MLP | 0.97 | 0.92 | 0.081 | 0.290 | 0.060 | 0.273 | 0.004 | 0.072 |
| XGB | 0.719 | 0.908 | 0.517 | 0.385 | 0.529 | 0.299 | 0.033 | 0.079 |
| DT | 0.671 | 0.823 | 0.308 | 0.360 | 0.573 | 0.416 | 0.036 | 0.110 |
| GPRMT | 0.993 | 0.755 | 0.102 | 0.620 | 0.082 | 0.489 | 0.005 | 0.129 |
| RF | 0.375 | 0.746 | 0.809 | 0.562 | 0.789 | 0.498 | 0.050 | 0.131 |
| BT | 0.379 | 0.729 | 0.793 | 0.617 | 0.787 | 0.514 | 0.049 | 0.136 |
| GPRBF | 0.968 | 0.717 | 0.253 | 0.577 | 0.178 | 0.525 | 0.011 | 0.138 |
| ET | 0.420 | 0.705 | 0.532 | 0.476 | 0.761 | 0.536 | 0.048 | 0.141 |
| KNN | 0.844 | 0.624 | 0.270 | 0.555 | 0.395 | 0.605 | 0.025 | 0.160 |
| LC | 0.286 | 0.385 | 1.575 | 1.169 | 0.844 | 0.775 | 0.053 | 0.204 |
| RC | 0.273 | 0.359 | 1.539 | 1.187 | 0.851 | 0.791 | 0.054 | 0.208 |
| EN | 0.171 | 0.237 | 1.683 | 1.485 | 0.995 | 0.979 | 0.063 | 0.258 |
| BR | 0.007 | 0.018 | 1.699 | 1.494 | 0.999 | 0.989 | 0.063 | 0.261 |
| GPR | 0.000 | -0.003 | 0.517 | 0.385 | 0.529 | 0.299 | 0.033 | 0.079 |

**Table S25.** All metrics for TOF regression models in family 2 complexes.

| Model | Train R^2^ | Test R^2^ | Train MAE | Test MAE | Train MSE | Test MSE | Train RMSE | Test RMSE | Train ex. variance | Test ex. variance | |
| --- | --- | --- | --- | --- | --- | --- | --- | --- | --- | --- | --- |
| Linear models | | | | | | | | | | |  |
| LC | 0.372 | 0.348 | 2233.510 | 2056.590 | 20274370.740 | 22099724.710 | 4502.707 | 4701.034 | 0.372 | 0.349 | |
| EN | 0.370 | 0.347 | 2244.450 | 2079.210 | 20334290.940 | 22124932.560 | 4509.356 | 4703.715 | 0.370 | 0.348 | |
| RC | 0.361 | 0.332 | 2259.930 | 2107.200 | 20633808.400 | 22622866.540 | 4542.445 | 4756.350 | 0.361 | 0.333 | |
| BR | 0.011 | 0.004 | 2402.190 | 2372.390 | 31946400.640 | 33730731.910 | 5652.115 | 5807.816 | 0.011 | 0.005 | |
| Nearest Neighbor models | | | | | | | | | | |  |
| KNN | 0.899 | 0.692 | 500.610 | 786.110 | 3249298.760 | 10431281.810 | 1802.581 | 3229.749 | 0.900 | 0.694 | |
| Gaussian Process Regressor models | | | | | | | | | | |  |
| GPRMT | 0.991 | 0.749 | 176.990 | 1216.720 | 285482.950 | 8498252.120 | 534.306 | 2915.176 | 0.991 | 0.754 | |
| GPRBF | 0.987 | 0.747 | 204.970 | 1292.610 | 412921.680 | 8574171.780 | 642.590 | 2928.169 | 0.987 | 0.753 | |
| GPR | 0.000 | 0.000 | 2429.090 | 2363.020 | 32290545.850 | 33897460.730 | 5682.477 | 5822.153 | 0.372 | 0.349 | |
| Tree and ensemble models | | | | | | | | | | |  |
| ET | 0.997 | 0.962 | 65.010 | 220.360 | 107226.930 | 1281911.570 | 327.455 | 1132.215 | 0.997 | 0.963 | |
| DT | 1.000 | 0.954 | 8.360 | 249.460 | 2054.390 | 1541760.820 | 45.325 | 1241.677 | 1.000 | 0.955 | |
| XGB | 0.965 | 0.871 | 365.840 | 634.570 | 1143899.75 | 4376369.500 | 1069.532 | 2091.977 | 0.965 | 0.871 | |
| AB | 1.000 | 0.821 | 7.750 | 481.120 | 2192.950 | 6081246.170 | 46.829 | 2466.018 | 1.000 | 0.824 | |
| GBDT | 1.000 | 0.657 | 8.160 | 640.990 | 2054.120 | 11604701.070 | 45.322 | 3406.567 | 1.000 | 0.665 | |
| BT | 0.630 | 0.580 | 1074.480 | 1128.950 | 11954022.790 | 14242886.800 | 3457.459 | 3773.975 | 0.630 | 0.580 | |
| RF | 0.620 | 0.544 | 1012.430 | 1134.570 | 12266060.470 | 15442118.130 | 3502.294 | 3929.646 | 0.621 | 0.545 | |
| Neural network | | | | | | | | | | | |
| MLP | 1.00 | 0.97 | 9.21 | 259.98 | 2040.83 | 804276.3 | 45.18 | 896.81 | 1.00 | 0.97 | |

**Table S26.** All metrics for TON regression models in family 2 complexes.

| Model | Train R^2^ | Test R^2^ | Train MAE | Test MAE | Train MSE | Test MSE | Train RMSE | Test RMSE | Train ex. variance | Test ex. variance |
| --- | --- | --- | --- | --- | --- | --- | --- | --- | --- | --- |
| Linear models | | | | | | | | | | |
| LC | 0.286 | 0.385 | 1364.210 | 1163.190 | 10105971.760 | 2962253.170 | 3178.989 | 1721.120 | 0.286 | 0.385 |
| RC | 0.273 | 0.359 | 1333.510 | 1181.110 | 10292210.340 | 3087384.290 | 3208.147 | 1757.095 | 0.273 | 0.359 |
| EN | 0.171 | 0.237 | 1344.700 | 1291.460 | 11737835.290 | 3672083.080 | 3426.052 | 1916.268 | 0.171 | 0.237 |
| BR | 0.007 | 0.018 | 1457.960 | 1477.950 | 14062051.290 | 4729395.230 | 3749.940 | 2174.717 | 0.007 | 0.018 |
| Nearest Neighbor models | | | | | | | | | | |
| KNN | 0.844 | 0.624 | 233.820 | 552.100 | 2210431.160 | 1809435.190 | 1486.752 | 1345.152 | 0.844 | 0.624 |
| Gaussian Process Regressor models | | | | | | | | | | |
| GPRMT | 0.993 | 0.755 | 88.580 | 617.150 | 95371.520 | 1178223.950 | 308.823 | 1085.460 | 0.993 | 0.755 |
| GPRBF | 0.968 | 0.717 | 219.300 | 574.590 | 449263.440 | 1361874.110 | 670.271 | 1166.994 | 0.968 | 0.717 |
| GPR | 0.000 | -0.003 | 1471.380 | 1486.650 | 14157270.540 | 4831399.920 | 3762.615 | 2198.045 | -0.000 | -0.003 |
| Tree and ensemble models | | | | | | | | | | |
| DT | 0.671 | 0.823 | 266.390 | 358.750 | 4661815.400 | 853495.330 | 2159.124 | 923.848 | 0.671 | 0.823 |
| ET | 0.420 | 0.705 | 461.010 | 473.890 | 8215638.270 | 1419132.360 | 2866.293 | 1191.273 | 0.420 | 0.705 |
| GBDT | 0.996 | 0.991 | 47.080 | 101.670 | 54845.660 | 42280.650 | 234.192 | 205.623 | 0.996 | 0.991 |
| XGB | 0.719 | 0.908 | 447.880 | 382.990 | 3971301.500 | 442443.440 | 1992.812 | 665.164 | 0.720 | 0.908 |
| AB | 0.996 | 0.981 | 42.910 | 141.020 | 54367.080 | 92343.390 | 233.167 | 303.881 | 0.996 | 0.981 |
| BT | 0.379 | 0.729 | 686.620 | 614.500 | 8786351.670 | 1306527.500 | 2964.178 | 1143.034 | 0.380 | 0.729 |
| RF | 0.375 | 0.746 | 701.000 | 559.380 | 8847663.190 | 1225025.990 | 2974.502 | 1106.809 | 0.375 | 0.746 |
| Neural network | | | | | | | | | | |
| MLP | 1.00 | 0.92 | 71.11 | 261.47 | 54628.19 | 403812.66 | 233.73 | 635.46 | 1.00 | 0.92 |

**Table S27.** Metrics for the model trained on data scaled by log10(x+1) for family 2 catalysts dataset. The "scaled" label identifies the metrics obtained with prediction on a logarithmic scale, while the "back-conv" indicates the corresponding prediction when the predicted values (y) are converted to the original scale (y’=exp(y) - 1). For comparison, the accuracy of the models for the original/unscaled data is also reported.

| Model name | TOF original | | TOF scaled | | TOF back-conv | | TON original | | TON back-conv | | TON back-conv | |
| --- | --- | --- | --- | --- | --- | --- | --- | --- | --- | --- | --- | --- |
|  | Train R^2^ | Test R^2^ | Train R^2^ | Test R^2^ | Train R^2^ | Test R^2^ | Train R^2^ | Test R^2^ | Train R^2^ | Test R^2^ | Train R^2^ | Test R^2^ |
| Linear models | | | | | | | | | | | | |
| LC | 0.372 | 0.348 | 0.688 | 0.722 | 0.065 | 0.079 | 0.286 | 0.385 | 0.607 | 0.734 | 0.047 | 0.189 |
| RC | 0.361 | 0.332 | 0.687 | 0.715 | 0.058 | 0.074 | 0.273 | 0.359 | 0.618 | 0.750 | 0.067 | 0.259 |
| BR | 0.011 | 0.004 | 0.685 | 0.706 | 0.053 | 0.070 | 0.171 | 0.237 | 0.615 | 0.744 | 0.063 | 0.241 |
| EN | 0.370 | 0.347 | 0.688 | 0.724 | 0.064 | 0.078 | 0.007 | 0.018 | 0.607 | 0.734 | 0.047 | 0.189 |
| Nearest Neighbor models | | | | | | | | | | | | |
| KNN | 0.899 | 0.692 | 0.940 | 0.761 | 0.906 | 0.702 | 0.375 | 0.746 | 0.887 | 0.822 | 0.710 | 0.613 |
| Gaussian Process Regressor models | | | | | | | | | | | | |
| GPR | 0.000 | 0.000 | 0.000 | -0.030 | -0.057 | -0.045 | -0.051 | -0.198 | -0.000 | -0.003 | 0.000 | -0.024 |
| GPRBF | 0.987 | 0.747 | 0.948 | 0.850 | 0.496 | 0.363 | 0.783 | 0.801 | 0.379 | 0.729 | 0.936 | 0.797 |
| GPRMT | 0.991 | 0.749 | 0.949 | 0.854 | 0.494 | 0.359 | 0.783 | 0.783 | 0.968 | 0.717 | 0.934 | 0.801 |
| Tree and ensemble models | | | | | | | | | | | | |
| DT | 1.000 | 0.954 | 0.982 | 0.898 | 1.000 | 0.954 | 0.996 | 0.981 | 0.924 | 0.869 | 0.676 | 0.944 |
| ET | 0.997 | 0.962 | 0.972 | 0.937 | 0.975 | 0.944 | 0.671 | 0.823 | 0.929 | 0.862 | 0.908 | 0.889 |
| GBDT | 1.000 | 0.657 | 0.981 | 0.932 | 0.995 | 0.986 | 0.996 | 0.991 | 0.960 | 0.890 | 0.628 | 0.945 |
| XGB | 0.965 | 0.871 | 0.963 | 0.933 | 0.952 | 0.965 | 0.719 | 0.908 | 0.848 | 0.848 | 0.848 | 0.848 |
| AB | 1.000 | 0.821 | 0.980 | 0.836 | 0.978 | 0.883 | 0.993 | 0.755 | 0.968 | 0.901 | 0.995 | 0.905 |
| BT | 0.580 | 0.580 | 0.764 | 0.761 | 0.032 | 0.043 | 0.007 | 0.018 | 0.741 | 0.750 | 0.168 | 0.452 |
| RF | 0.420 | 0.705 | 0.738 | 0.752 | 0.158 | 0.419 | 0.420 | 0.705 | 0.738 | 0.752 | 0.158 | 0.419 |

**Table S28.** Metrics for linear models trained on polynomial features generated from the standardized data of family 2 dataset. In the TOF poly, the original target values were used, while in TOF poly back-conv, the targets were initially scaled to log10(x+1) and then converted back to the original scale.

| Model name | TOF poly | | TOF poly back-conv | | TON poly | | TON poly back-conv | |
| --- | --- | --- | --- | --- | --- | --- | --- | --- |
|  | Train R^2^ | Test R^2^ | Train R^2^ | Test R^2^ | Train R^2^ | Test R^2^ | Train R^2^ | Test R^2^ |
| LC | 0.589 | 0.510 | 0.550 | 0.497 | 0.782 | 0.275 | 0.773 | 0.876 |
| RC | 0.580 | 0.549 | 0.618 | 0.638 | 0.872 | -1.032 | 0.662 | 0.802 |
| BR | 0.528 | 0.515 | 0.589 | 0.604 | 0.460 | 0.675 | 0.731 | 0.867 |
| EN | 0.535 | 0.522 | 0.461 | 0.423 | 0.643 | 0.822 | 0.641 | 0.872 |

| a)  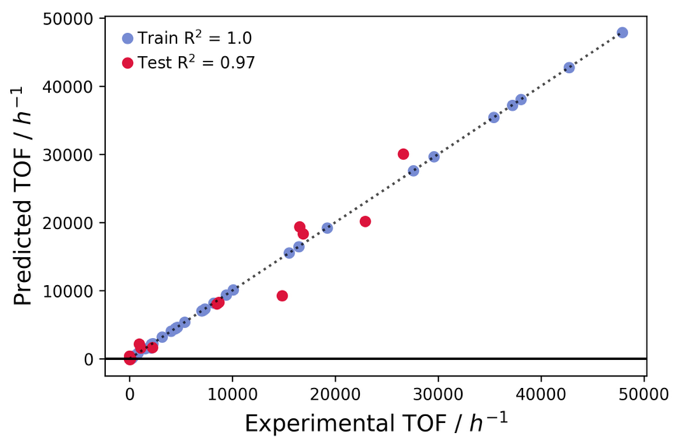 | b)  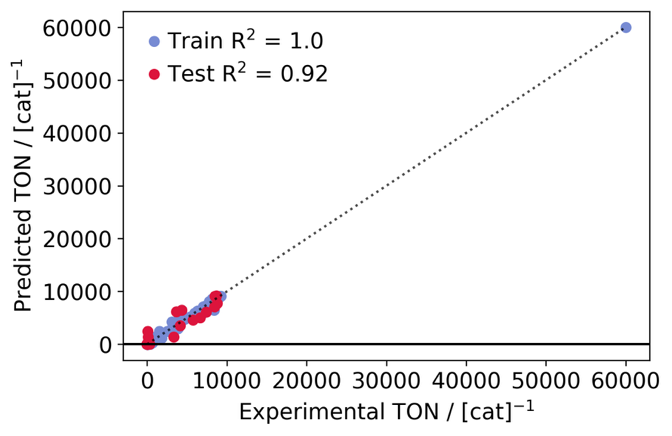 |
| --- | --- |

**Figure S24.** Accuracy plots of MLP models for (a) TOF and (b) TON for family 2 dataset.

## Regression of Family 2 dataset with Family 1 features list

**Table S29**. Accuracy of ML regression models on training and test prediction for TOF (h-1) and TON (cat-1) of family 2 catalysts using family 1 features set.

| TOF | | | TON | | |
| --- | --- | --- | --- | --- | --- |
| Model | Train R^2^ | Test R^2^ | Model | Train R^2^ | Test R^2^ |
| KNN | 0.4998 | 0.4998 | DT | 0.9782 | 0.8831 |
| ET | 0.4843 | 0.4843 | GDBT | 0.9781 | 0.8795 |
| DT | 0.5050 | 0.5050 | XGB | 0.9682 | 0.8778 |
| GPRBF | 0.5534 | 0.5534 | ET | 0.9737 | 0.8639 |
| GPRMT | 0.5660 | 0.5660 | AB | 0.9781 | 0.8624 |
| AB | 0.5674 | 0.5674 | GPRMT | 0.9712 | 0.8552 |
| GDBT | 0.5022 | 0.5022 | KNN | 0.5379 | 0.8527 |
| RC | 0.2846 | 0.2846 | GPRBF | 0.9698 | 0.8415 |
| LC | 0.2850 | 0.2850 | RF | 0.3876 | 0.8254 |
| EC | 0.2850 | 0.2850 | RC | 0.9651 | 0.7850 |
| XGB | 0.3336 | 0.3336 | LC | 0.9649 | 0.7825 |
| BR | 0.1517 | 0.1517 | EC | 0.9648 | 0.7815 |
| BT | 0.0604 | 0.0604 | BR | 0.9641 | 0.7717 |
| RF | 0.0643 | 0.0643 | BT | 0.4406 | 0.6516 |
| GPR | 0.0000 | -0.0005 | GPR | 0.0000 | -0.0444 |

## Additional SHAP and PDP analysis on family 2 (Nishibayashi’s) catalysts

| a)  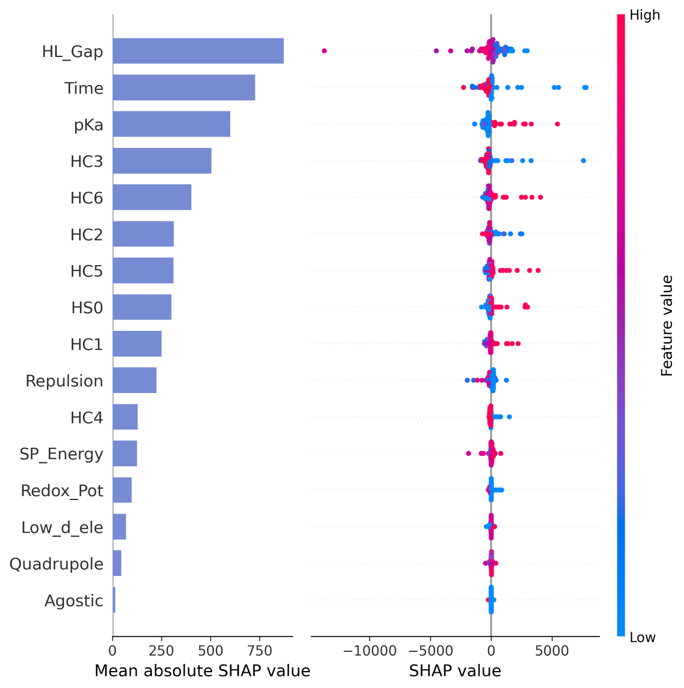 | b)  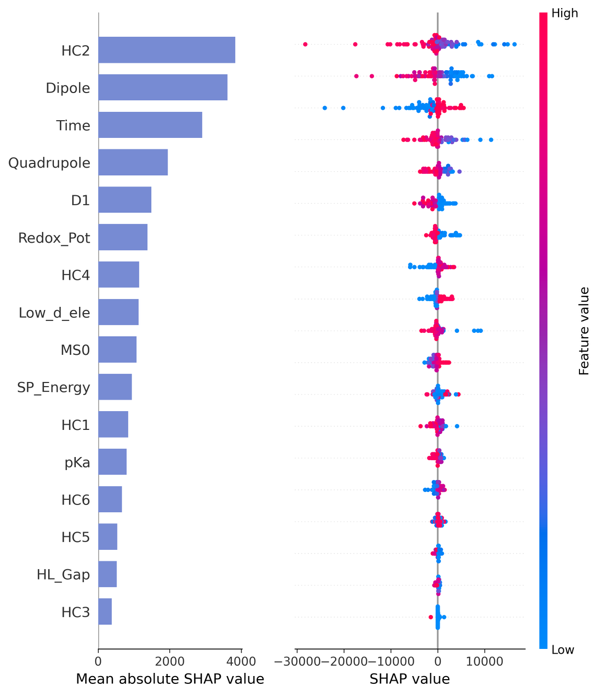 |
| --- | --- |

**Figure S25.** Feature importance and impact on the predicted value by SHAP analysis of the trained MLP models for family 2 dataset for (a) TOF and (b) TON prediction.


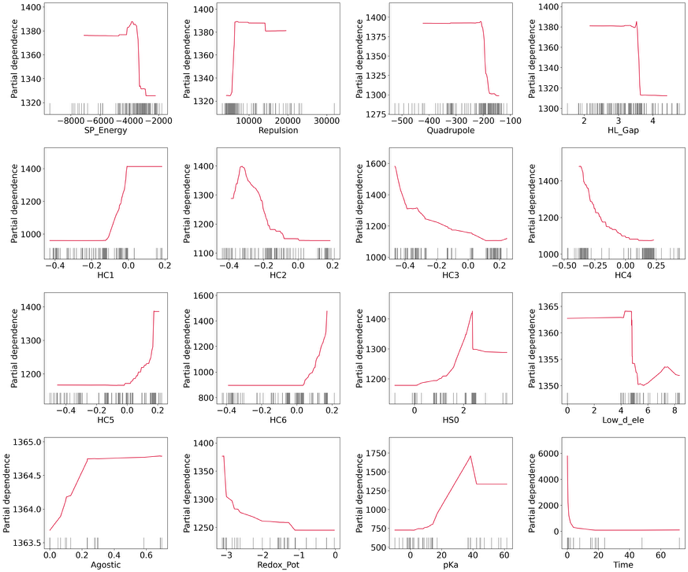


**Figure S26.** Partial dependence plots (PDP) for TOF (ET model) ML regression models for family 2 dataset. The partial dependence was calculated between the (0.05 and 0.95) percentile and it is shown in red, while the dataset values of each feature are displayed in gray.


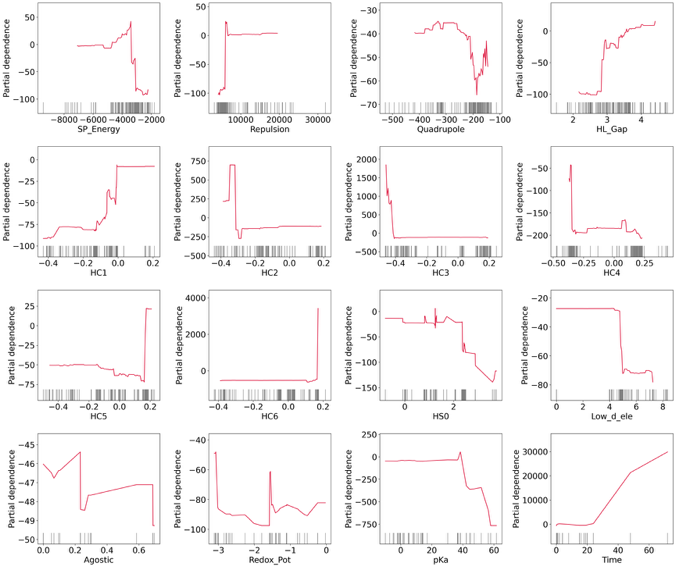


**Figure S27.** Partial dependence plots (PDP) for TON (GDBT model) ML regression models for family 2 dataset. The partial dependence was calculated between the (0.05 and 0.95) percentile and it is shown in red, while the dataset values of each feature are displayed in gray.

# Combined models

**Table S30.** All metrics for ML regression models for TOF over family 1 and 2 combined datasets.

| Model | Train R^2^ | Test R^2^ | Train RMAE | Test RMAE | Train RRMSE | Test RRMSE | Train NRMSE | Test NRMSE |
| --- | --- | --- | --- | --- | --- | --- | --- | --- |
| DT | 0.96 | 0.95 | 0.129 | 0.244 | 0.195 | 0.215 | 0.023 | 0.024 |
| GDBT | 1.0 | 0.86 | 0.0003 | 0.390 | 0.0003 | 0.371 | 0.0001 | 0.042 |
| RF | 0.07 | 0.06 | 1.413 | 1.363 | 0.965 | 0.965 | 0.114 | 0.109 |
| ET | 0.98 | 0.93 | 0.133 | 0.235 | 0.129 | 0.259 | 0.015 | 0.029 |
| AB | 1.0 | 0.85 | 0.0002 | 0.417 | 0.0003 | 0.391 | 0.0001 | 0.044 |
| XGB | 1.0 | 0.91 | 0.001 | 0.288 | 0.0005 | 0.298 | 0.0001 | 0.034 |

**Table S31.** All metrics for ML regression models for TON over family 1 and 2 combined datasets.

| Model | Train R^2^ | Test R^2^ | Train RMAE | Test RMAE | Train RRMSE | Test RRMSE | Train NRMSE | Test NRMSE |
| --- | --- | --- | --- | --- | --- | --- | --- | --- |
| DT | 0.98 | 0.88 | 0.204 | 0.307 | 0.152 | 0.347 | 0.009 | 0.074 |
| GDBT | 0.98 | 0.90 | 0.150 | 0.307 | 0.141 | 0.312 | 0.008 | 0.066 |
| RF | 0.40 | 0.80 | 0.774 | 0.714 | 0.775 | 0.446 | 0.046 | 0.095 |
| ET | 0.98 | 0.91 | 0.194 | 0.296 | 0.144 | 0.302 | 0.009 | 0.064 |
| AB | 0.98 | 0.90 | 0.168 | 0.284 | 0.146 | 0.315 | 0.009 | 0.067 |
| XGB | 0.88 | 0.89 | 0.346 | 0.386 | 0.349 | 0.323 | 0.021 | 0.069 |

# Energy span regression models

**Table S32.** All metrics for energy span regression models over family 1 datasets.

| Model | Train R^2^ | Test R^2^ | Train RMAE | Test RMAE | Train RRMSE | Test RRMSE | Train NRMSE | Test NRMSE |
| --- | --- | --- | --- | --- | --- | --- | --- | --- |
| DT | 0.74 | 0.57 | 0.0529 | 0.0861 | 0.5082 | 0.6381 | 0.1035 | 0.1996 |
| BT | 0.35 | 0.23 | 0.0864 | 0.1112 | 0.8032 | 0.8548 | 0.1635 | 0.2674 |
| GBDT | 0.7 | 0.59 | 0.0553 | 0.0857 | 0.5426 | 0.6209 | 0.1105 | 0.1942 |
| RF | 0.63 | 0.32 | 0.0707 | 0.1117 | 0.6049 | 0.804 | 0.1231 | 0.2515 |
| ET | 0.72 | 0.62 | 0.0567 | 0.081 | 0.5215 | 0.6017 | 0.1062 | 0.1882 |
| AB | 0.75 | 0.58 | 0.0514 | 0.0887 | 0.5008 | 0.6285 | 0.102 | 0.1966 |
| XGB | 0.42 | 0.25 | 0.0853 | 0.1088 | 0.7566 | 0.8439 | 0.154 | 0.264 |

**Table S33.** All metrics for energy span regression models over family 2 datasets.

| Model | Train R^2^ | Test R^2^ | Train RMAE | Test RMAE | Train RRMSE | Test RRMSE | Train NRMSE | Test NRMSE |
| --- | --- | --- | --- | --- | --- | --- | --- | --- |
| DT | 0.93 | 0.75 | 0.0356 | 0.0595 | 0.2693 | 0.4965 | 0.0831 | 0.1625 |
| BT | 0.67 | 0.63 | 0.1035 | 0.1 | 0.5729 | 0.6007 | 0.1768 | 0.1966 |
| GBDT | 0.93 | 0.76 | 0.0319 | 0.0603 | 0.2614 | 0.4899 | 0.0807 | 0.1604 |
| RF | 0.67 | 0.64 | 0.1012 | 0.0955 | 0.5759 | 0.5977 | 0.1778 | 0.1957 |
| ET | 0.93 | 0.8 | 0.0333 | 0.0595 | 0.2623 | 0.4401 | 0.081 | 0.1441 |
| AB | 0.93 | 0.82 | 0.0325 | 0.0539 | 0.2634 | 0.4196 | 0.0813 | 0.1373 |
| XGB | 0.93 | 0.79 | 0.0377 | 0.0617 | 0.2682 | 0.4604 | 0.0828 | 0.1507 |

| a)  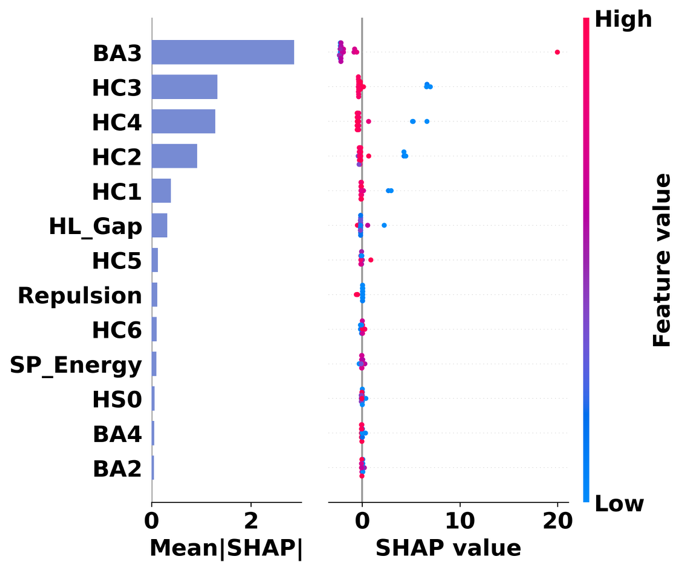 | b)  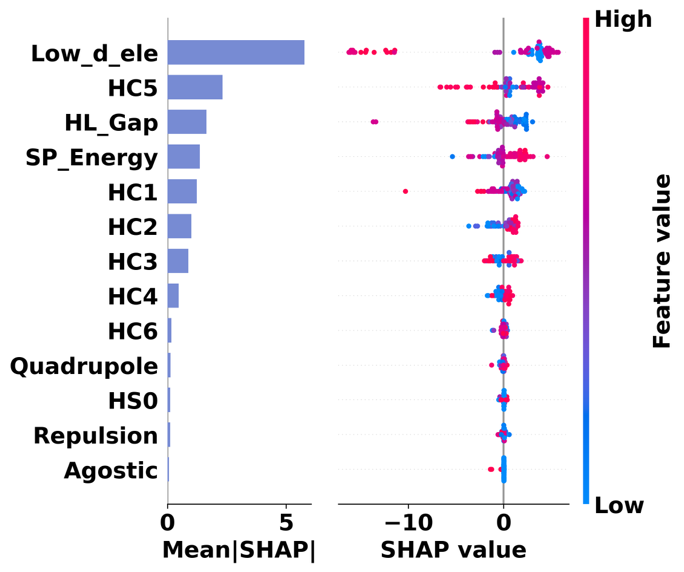 |
| --- | --- |

**Figure S28.** Feature importance and impact on the predicted value by SHAP analysis of the trained ML models for energy span prediction for (a) family 1 (ET model) and (b) family 2 dataset (AB model).

# Prediction of candidate catalysts


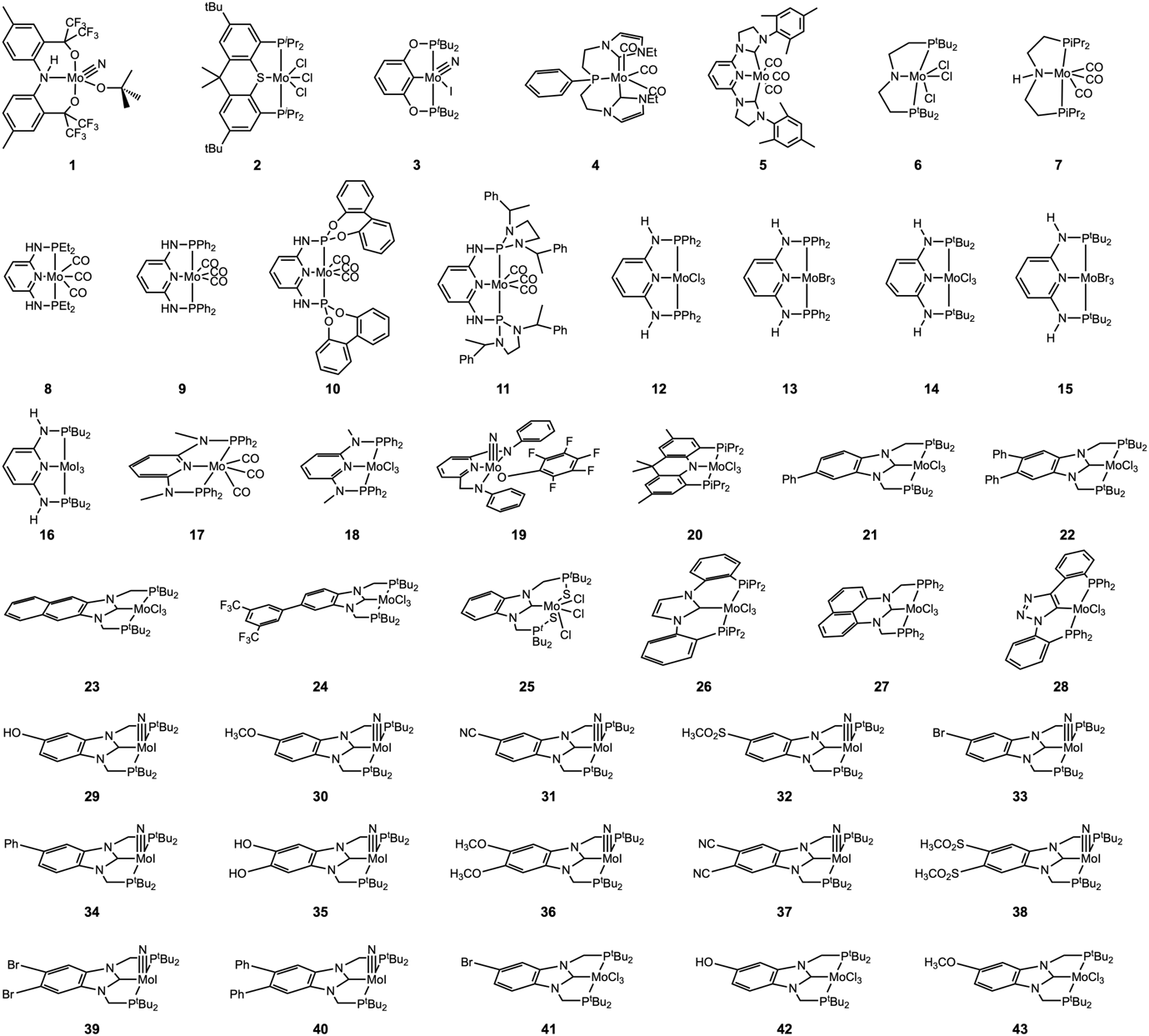


**Figure S29.** Structures of the reported complexes applied for the prediction of TOF and TON in the nitrogen reduction reaction.

**Table S34.** Prediction of TOF and TON for the external dataset of structures represented in Figure S28 using different experimental conditions for each candidate, inspired by structure similarity in the database. The predictions were obtained with the three best performing models for TOF (ET, DT and MLP) and TON (GDBT, AB and MLP) in family 2. In bold are the seven candidates with a high score.

| Structure name | Differentiated experimental conditions ^a^ | | | | | |
| --- | --- | --- | --- | --- | --- | --- |
|  | TOF [h^-1^] | | | TON | | |
|  | ET | DT | MLP | GDBT | AB | MLP |
| 1 | 3893.71 | 2.45 | -819.48 | -8.21 | 26.80 | 16.09 |
| 2 | 2691.60 | 2.78 | 9.05 | -445.78 | 6.60 | 16.09 |
| 3 | 28.74 | 3.60 | 76.39 | 131.42 | 59.00 | 25.27 |
| 4 | 3236.82 | 9.53 | -98.79 | -533.11 | 7.60 | **375.59** |
| 5 | 1792.87 | 9.53 | 13.86 | -548.68 | 7.30 | 8.33 |
| 6 | 7.26 | 2.25 | -71.92 | 158.30 | 41.50 | 11.20 |
| 7 | 0.99 | 0.01 | 0.00 | -286.87 | 0.90 | -19.13 |
| 8 | 8.58 | 0.01 | -1.56 | -786.61 | 39.40 | **213.64** |
| 9 | 8.47 | 0.01 | -0.57 | 52.20 | 50.00 | -12.54 |
| 10 | 16.00 | 0.01 | -1.14 | 344.33 | 50.00 | -21.02 |
| 11 | 23.47 | 0.01 | 0.06 | 336.43 | 50.00 | 6.43 |
| 12 | 51.95 | 0.16 | 306.20 | 2331.48 | 109.20 | 13.94 |
| 13 | 2.96 | 0.16 | 10.38 | -105.96 | 8.40 | 20.38 |
| 14 | 20.72 | 0.16 | 257.78 | 1765.03 | 50.00 | 10.40 |
| 15 | 4.25 | 0.16 | 10.41 | -7.29 | 4.90 | 20.47 |
| 16 | 3.30 | 0.16 | 0.65 | -150.51 | 8.95 | 15.31 |
| 17 | 8.26 | 0.01 | -0.37 | 170.64 | 1.00 | -9.64 |
| 18 | 85.97 | 0.16 | 336.13 | 2475.07 | 109.20 | 16.09 |
| 19 | 2531.40 | 2.45 | 388.94 | -706.87 | 12.00 | 16.09 |
| 20 | 2156.73 | 9.53 | 3.36 | -171.71 | 7.70 | 40.38 |
| 21 | **12856.12** | **16440.00** | **9005.15** | **8213.55** | **2520.00** | 16.09 |
| 22 | **13057.46** | **16860.00** | **7224.81** | **8055.11** | **2520.00** | 16.09 |
| 23 | **12164.06** | **9380.00** | **47561.30** | **2571.79** | **1159.00** | 16.09 |
| 24 | **13505.11** | **16860.00** | **11110.48** | **6848.20** | **7420.00** | 16.09 |
| 25 | 4532.10 | 9.53 | 809.00 | 71.78 | 50.00 | 16.09 |
| 26 | 22.13 | 4.00 | 1.98 | -452.64 | 405.33 | -17.46 |
| 27 | 137.46 | 2.75 | 517.49 | 566.80 | 44.00 | 16.09 |
| 28 | 39.27 | 2.75 | 106.61 | -696.33 | 90.00 | 16.09 |
| 29 | 1519.03 | 30.44 | 4.18 | 52.33 | 12.20 | 16.75 |
| 30 | 1581.49 | 30.44 | 1.59 | -403.66 | 405.33 | 14.73 |
| 31 | 1511.92 | 2.78 | 2889.86 | -9.07 | 7.70 | 20.67 |
| 32 | 1542.40 | 30.44 | 4.85 | -32.44 | 12.00 | 24.70 |
| 33 | 1712.79 | 42.28 | 6.50 | -91.63 | 18.20 | 25.18 |
| 34 | 1607.99 | 30.44 | 1.26 | -460.28 | 405.33 | 16.21 |
| 35 | 1581.59 | 30.44 | 1.20 | -389.17 | 405.33 | 13.91 |
| 36 | 1581.49 | 30.44 | 1.58 | -403.66 | 405.33 | 14.73 |
| 37 | 1552.23 | 2.78 | 4.18 | 5.00 | 11.20 | 23.11 |
| 38 | 1673.05 | 2.78 | 5.66 | -31.64 | 11.10 | 25.16 |
| 39 | 1673.10 | 42.28 | 3979.34 | -94.07 | 9.20 | 22.68 |
| 40 | 1795.95 | 2.45 | 6.16 | -13.13 | 7.90 | 18.17 |
| 41 | **13215.37** | **16440.00** | **19499.22** | **7782.98** | **6650.00** | 16.09 |
| 42 | **10858.86** | **9380.00** | **39295.33** | **4541.25** | **2520.00** | 16.09 |
| 43 | **11255.61** | **9380.00** | **34578.74** | **6385.86** | **1840.00** | 16.09 |

^a^ The specific experimental conditions selected for each candidate are listed in Table S37

**Table S35.** Selected TOF and TON predictions using the best performing models for the prediction of TOF and TON in family 2 catalysts, and compared with recent experimental data. The experimental conditions were adopted from ref. [28]: THF solvent, H_2_O as acid with 28800 eq, SmI_2_(THF)_2_ as reductant with 28800 eq, with reaction time of 5 min.

| Structure  Name | TOF | | | TON | | | |  |
| --- | --- | --- | --- | --- | --- | --- | --- | --- |
|  | ET | DT | Ref. [28] | | GDBT | AB | Ref. [28] | |
| 21 | 30487.47 | 47880.19 | 42720 ± 660 | | 3320.00 | 1840.00 | 3560 ± 54 | |
| 22 | 30535.16 | 47880.19 | 37080 ± 1320 | | 3118.38 | 1840.00 | 3090 ± 65 | |
| 23 | 29765.28 | 35400.14 | 49200 ± 5640 | | 1617.31 | 1386.00 | 4460 ± 470 | |
| 24 | 31422.48 | 37200.15 | 54240 ± 6960 | | 2535.08 | 3560.00 | 4520 ± 580 | |
| 41 | 30779.67 | 37200.15 | - | | 3061.10 | 3170.00 | - | |
| 42 | 26033.27 | 35400.14 | - | | 2148.39 | 1600.00 | - | |
| 43 | 26523.58 | 35400.14 | - | | 3442.97 | 1840.00 | - | |

**Table S36.** Selected TOF and TON predictions using the best performing models for the prediction of TOF and TON in family 2 catalysts, and compared with recent experimental data. The experimental conditions were adopted from ref. [28]: THF solvent, H_2_O as acid with 28800 eq, SmI_2_(THF)_2_ as reductant with 28800 eq, with reaction time of 2 h.

| Structure  Name | TOF | | | TON | | | |  |
| --- | --- | --- | --- | --- | --- | --- | --- | --- |
|  | ET | DT | Ref. [28] | | GDBT | AB | Ref. [28] | |
| 21 | 3695.30 | 4620.00 | - | | 9313.53 | 2520.00 | 8160 ± 330 | |
| 22 | 3707.82 | 4620.00 | - | | 9014.88 | 2520.00 | 8490 ± 400 | |
| 23 | 3538.92 | 4025.00 | - | | 3073.78 | 1159.00 | 8674 ± 490 | |
| 24 | 3819.20 | 4620.00 | - | | 7626.47 | 8610.00 | 8740 ± 290 | |
| 41 | 3726.03 | 4620.00 | - | | 8783.72 | 8050.00 | - | |
| 42 | 3311.94 | 4025.00 | - | | 7135.86 | 2520.00 | - | |
| 43 | 3369.45 | 4025.00 | - | | 7475.75 | 2520.00 | - | |

**Table S37.** The different experimental conditions applied for the prediction of TOF and TON for the external dataset of structures.

| Structure name | Solvent | Reductant | Reductant equivalents | Acid | Acid equivalents | Temperature [°C] | Time  [h] |
| --- | --- | --- | --- | --- | --- | --- | --- |
| 1 | THF | SmI_2_(THF)_2_ | 28800 | H_2_O | 28800 | 25 | 0.5 |
| 2 | THF | SmI_2_(THF)_2_ | 28800 | H_2_O | 28800 | 25 | 0.5 |
| 3 | diethylether | KC_8_ | 8000 | [H(OEt_2_)_2_] | 7360 | -78 | 1 |
| 4 | THF | SmI_2_(THF)_2_ | 28800 | H_2_O | 28800 | 25 | 0.5 |
| 5 | THF | SmI_2_(THF)_2_ | 28800 | H_2_O | 28800 | 25 | 0.5 |
| 6 | toluene | CoCp^*^_2_ | 36 | [ColH] | 48 | 25 | 20 |
| 7 | toluene | CoCp^*^_2_ | 36 | [ColH] | 48 | 25 | 20 |
| 8 | toluene | CoCp_2_ | 36 | [LutH] | 48 | 25 | 20 |
| 9 | toluene | CoCp_2_ | 36 | [LutH] | 48 | 25 | 20 |
| 10 | toluene | CoCp_2_ | 36 | [LutH] | 48 | 25 | 20 |
| 11 | toluene | CoCp_2_ | 36 | [LutH] | 48 | 25 | 20 |
| 12 | toluene | CoCp_2_ | 36 | [LutH] | 48 | 25 | 20 |
| 13 | toluene | CoCp_2_ | 36 | [LutH] | 48 | 25 | 20 |
| 14 | toluene | CoCp_2_ | 36 | [LutH] | 48 | 25 | 20 |
| 15 | toluene | CoCp_2_ | 36 | [LutH] | 48 | 25 | 20 |
| 16 | toluene | CoCp_2_ | 36 | [LutH] | 48 | 25 | 20 |
| 17 | toluene | CoCp_2_ | 36 | [LutH] | 48 | 25 | 20 |
| 18 | toluene | CoCp_2_ | 36 | [LutH] | 48 | 25 | 20 |
| 19 | THF | SmI_2_(THF)_2_ | 28800 | H_2_O | 28800 | 25 | 0.5 |
| 20 | THF | SmI_2_(THF)_2_ | 28800 | H_2_O | 28800 | 25 | 0.5 |
| 21 | THF | SmI_2_(THF)_2_ | 28800 | H_2_O | 28800 | 25 | 0.5 |
| 22 | THF | SmI_2_(THF)_2_ | 28800 | H_2_O | 28800 | 25 | 0.5 |
| 23 | THF | SmI_2_(THF)_2_ | 28800 | H_2_O | 28800 | 25 | 0.5 |
| 24 | THF | SmI_2_(THF)_2_ | 28800 | H_2_O | 28800 | 25 | 0.5 |
| 25 | THF | SmI_2_(THF)_2_ | 28800 | H_2_O | 28800 | 25 | 0.5 |
| 26 | toluene | CoCp^*^_2_ | 180 | [ColH] | 240 | 25 | 20 |
| 27 | toluene | CoCp^*^_2_ | 180 | [ColH] | 240 | 25 | 20 |
| 28 | toluene | CoCp^*^_2_ | 180 | [ColH] | 240 | 25 | 20 |
| 29 | THF | SmI_2_(THF)_2_ | 28800 | H_2_O | 28800 | 25 | 0.5 |
| 30 | THF | SmI_2_(THF)_2_ | 28800 | H_2_O | 28800 | 25 | 0.5 |
| 31 | THF | SmI_2_(THF)_2_ | 28800 | H_2_O | 28800 | 25 | 0.5 |
| 32 | THF | SmI_2_(THF)_2_ | 28800 | H_2_O | 28800 | 25 | 0.5 |
| 33 | THF | SmI_2_(THF)_2_ | 28800 | H_2_O | 28800 | 25 | 0.5 |
| 34 | THF | SmI_2_(THF)_2_ | 28800 | H_2_O | 28800 | 25 | 0.5 |
| 35 | THF | SmI_2_(THF)_2_ | 28800 | H_2_O | 28800 | 25 | 0.5 |
| 36 | THF | SmI_2_(THF)_2_ | 28800 | H_2_O | 28800 | 25 | 0.5 |
| 37 | THF | SmI_2_(THF)_2_ | 28800 | H_2_O | 28800 | 25 | 0.5 |
| 38 | THF | SmI_2_(THF)_2_ | 28800 | H_2_O | 28800 | 25 | 0.5 |
| 39 | THF | SmI_2_(THF)_2_ | 28800 | H_2_O | 28800 | 25 | 0.5 |
| 40 | THF | SmI_2_(THF)_2_ | 28800 | H_2_O | 28800 | 25 | 0.5 |
| 41 | THF | SmI_2_(THF)_2_ | 28800 | H_2_O | 28800 | 25 | 0.5 |
| 42 | THF | SmI_2_(THF)_2_ | 28800 | H_2_O | 28800 | 25 | 0.5 |
| 43 | THF | SmI_2_(THF)_2_ | 28800 | H_2_O | 28800 | 25 | 0.5 |

**Table S38.** Prediction of TOF and TON for the external dataset of structures represented in Figure S28 using the general optimal conditions. The predictions were obtained with the three best performing models for TOF (ET, DT and MLP) and TON (GDBT, AB and MLP) in family 2. In bold are the candidates with a high score.

| Structure name | General optimal conditions ^a^ | | | | | |
| --- | --- | --- | --- | --- | --- | --- |
|  | TOF [h^-1^] | | | TON | | |
|  | ET | DT | MLP | GDBT | AB | MLP |
| 1 | 3893.71 | 2.45 | -819.48 | -8.21 | 26.80 | 16.09 |
| 2 | 2691.60 | 2.78 | 9.05 | -445.78 | 6.60 | 16.09 |
| 3 | 2034.93 | 2.78 | 5.41 | 77.89 | 59.00 | 23.04 |
| 4 | 3236.82 | 9.53 | -98.79 | -533.11 | 7.60 | 375.59 |
| 5 | 1792.87 | 9.53 | 13.86 | -548.68 | 7.30 | 8.33 |
| 6 | 5457.53 | 18.25 | 3688.55 | 402.25 | 89.00 | 16.09 |
| 7 | 3445.83 | 9.53 | -273.04 | -368.54 | 6.80 | 781.72 |
| 8 | 5126.20 | **9380.00** | -404.79 | 3394.96 | 44.10 | **5925.91** |
| 9 | 5098.82 | **9380.00** | -211.80 | 3035.07 | 1600.00 | 840.02 |
| 10 | 5137.59 | **9380.00** | -309.08 | 4281.52 | 5730.00 | **1847.16** |
| 11 | 5465.19 | **15520.00** | -148.02 | 4265.47 | **7020.00** | 19.02 |
| 12 | 8059.50 | 2.83 | **46031.63** | 4879.50 | 4130.00 | 243.75 |
| 13 | 2682.48 | 42.28 | 11.64 | -52.48 | 26.80 | 28.71 |
| 14 | 5271.60 | 18.25 | **11040.01** | 2036.83 | 2520.00 | 16.09 |
| 15 | 2075.92 | 42.28 | 11.98 | -41.04 | 51.00 | 29.15 |
| 16 | 1828.36 | 0.62 | 3.51 | 7.63 | 13.80 | 29.61 |
| 17 | 5083.52 | **9380.00** | -197.58 | 4204.81 | 5730.00 | 681.41 |
| 18 | 8283.68 | 2.83 | **37094.20** | **6208.47** | **7310.00** | 16.09 |
| 19 | 2531.40 | 2.45 | 388.94 | -706.87 | 12.00 | 16.09 |
| 20 | 2156.73 | 9.53 | 3.36 | -171.71 | 7.70 | 40.38 |
| 21 | **12856.12** | **16440.00** | 9005.15 | **8213.55** | 2520.00 | 16.09 |
| 22 | **13057.46** | **16860.00** | 7224.81 | **8055.11** | 2520.00 | 16.09 |
| 23 | **12164.06** | **9380.00** | **47561.30** | 2571.79 | 1159.00 | 16.09 |
| 24 | **13505.11** | **16860.00** | **11110.48** | **6848.20** | **7420.00** | 16.09 |
| 25 | 4532.10 | 9.53 | 809.00 | 71.78 | 50.00 | 16.09 |
| 26 | 3409.86 | 0.62 | 1.32 | -378.10 | 1299.00 | 28.28 |
| 27 | 9890.87 | 9.53 | **19725.43** | 209.14 | 50.00 | 16.09 |
| 28 | 6355.29 | 9.53 | 697.88 | 5513.96 | **7420.00** | 16.09 |
| 29 | 1519.03 | 30.44 | 4.18 | 52.33 | 12.20 | 16.75 |
| 30 | 1581.49 | 30.44 | 1.59 | -403.66 | 405.33 | 14.73 |
| 31 | 1511.92 | 2.78 | 2889.86 | -9.07 | 7.70 | 20.67 |
| 32 | 1542.40 | 30.44 | 4.85 | -32.44 | 12.00 | 24.70 |
| 33 | 1712.79 | 42.28 | 6.50 | -91.63 | 18.20 | 25.18 |
| 34 | 1607.99 | 30.44 | 1.26 | -460.28 | 405.33 | 16.21 |
| 35 | 1581.59 | 30.44 | 1.20 | -389.17 | 405.33 | 13.91 |
| 36 | 1581.49 | 30.44 | 1.58 | -403.66 | 405.33 | 14.73 |
| 37 | 1552.23 | 2.78 | 4.18 | 5.00 | 11.20 | 23.11 |
| 38 | 1673.05 | 2.78 | 5.66 | -31.64 | 11.10 | 25.16 |
| 39 | 1673.10 | 42.28 | 3979.34 | -94.07 | 9.20 | 22.68 |
| 40 | 1795.95 | 2.45 | 6.16 | -13.13 | 7.90 | 18.17 |
| 41 | **13215.37** | **16440.00** | **19499.22** | **7782.98** | **6650.00** | 16.09 |
| 42 | **10858.86** | **9380.00** | **39295.33** | 4541.25 | 2520.00 | 16.09 |
| 43 | **11255.61** | **9380.00** | **34578.74** | **6385.86** | 1840.00 | 16.09 |

^a^ THF solvent, H_2_O as acid with 1000 eq, SmI_2_(THF)_2_ as reductant with 1000 eq, 0.5 h of reaction time.

**Table S39.** Prediction of TOF and TON for the external dataset of structures represented in Figure S28 using general optimal conditions and 25 h for reaction time. The predictions were obtained with the three best performing models for TOF (ET, DT and MLP) and TON (GDBT, AB and MLP) in family 2. In bold are the candidates with a high score.

| Structure name | Optimal conditions with longer reaction time^a^ | | | | | |
| --- | --- | --- | --- | --- | --- | --- |
|  | TOF [h^-1^] | | | TON | | |
|  | ET | DT | MLP | GDBT | AB | MLP |
| 1 | 43.36 | 2.45 | 5.84 | 2262.88 | 40.50 | 16.09 |
| 2 | 21.76 | 2.78 | 8.17 | -1124.33 | 9.30 | 297.44 |
| 3 | 32.20 | 2.78 | 6.21 | 1919.18 | 59.00 | 296.21 |
| 4 | 55.00 | 9.53 | -166.05 | 4021.12 | 7.20 | 8972.00 |
| 5 | 13.01 | 9.53 | 9.25 | 3875.90 | 5.00 | 830.21 |
| 6 | 99.06 | 18.25 | 75.16 | 2845.86 | 84.00 | 370.55 |
| 7 | 66.42 | 9.53 | -299.49 | 2979.39 | 6.60 | **11890.67** |
| 8 | 121.27 | 5.46 | -353.29 | 3272.94 | 39.40 | **36829.37** |
| 9 | 121.16 | 5.46 | -139.35 | 4247.64 | 44.10 | **25177.32** |
| 10 | 156.00 | 5.46 | -154.79 | 4680.94 | 44.67 | **30342.73** |
| 11 | 182.97 | 30.67 | 5.10 | 4004.80 | 50.00 | **17415.64** |
| 12 | 193.70 | 2.83 | 139.21 | **8556.71** | 109.20 | 396.94 |
| 13 | 15.40 | 42.28 | 12.80 | 3492.13 | 26.80 | 21.72 |
| 14 | 82.53 | 18.25 | 105.45 | 7277.89 | 90.00 | 499.50 |
| 15 | 15.94 | 42.28 | 12.80 | 4312.18 | 41.50 | 22.53 |
| 16 | 10.66 | 0.62 | 3.23 | 3056.75 | 26.80 | 106.48 |
| 17 | 129.08 | 5.46 | -73.25 | 4857.93 | 44.67 | **25154.97** |
| 18 | 225.49 | 2.83 | 156.13 | **9437.80** | 109.20 | 642.28 |
| 19 | 24.29 | 2.45 | 11.12 | 1890.78 | 20.50 | 225.20 |
| 20 | 21.94 | 9.53 | 4.47 | 176.54 | 11.20 | 540.35 |
| 21 | **658.25** | **833.33** | 286.03 | **11302.67** | 553.33 | 1034.04 |
| 22 | **693.22** | 30.67 | **318.88** | **10060.59** | 388.00 | 785.40 |
| 23 | **615.85** | 5.46 | 235.34 | 6664.79 | **1159.00** | 1065.30 |
| 24 | **708.30** | 30.67 | **372.37** | 5890.00 | 104.50 | 16.09 |
| 25 | 141.01 | 9.53 | 8.64 | 5555.70 | 51.00 | 858.02 |
| 26 | 106.56 | 0.62 | 2.43 | 1643.04 | **1299.00** | **13199.90** |
| 27 | 479.39 | 9.53 | **455.26** | 1014.51 | 50.00 | 495.99 |
| 28 | 205.44 | 9.53 | 5.95 | 4738.04 | 90.00 | **68788.96** |
| 29 | 15.13 | 30.44 | 3.91 | 1464.29 | 50.00 | 271.55 |
| 30 | 14.55 | 30.44 | 13.50 | 1331.93 | 405.33 | 271.68 |
| 31 | 16.78 | 2.78 | 3.51 | -2096.76 | 39.40 | 563.10 |
| 32 | 20.09 | 30.44 | 5.01 | -1691.66 | 50.00 | 310.70 |
| 33 | 24.23 | 42.28 | 8.20 | -333.03 | 50.00 | 17.38 |
| 34 | 15.20 | 30.44 | 14.26 | 866.02 | 405.33 | 312.66 |
| 35 | 14.35 | 30.44 | 12.77 | 1045.97 | 405.33 | 248.15 |
| 36 | 14.55 | 30.44 | 13.50 | 1331.93 | 405.33 | 271.97 |
| 37 | 19.33 | 2.78 | 4.04 | -1769.04 | 50.00 | 278.37 |
| 38 | 23.53 | 2.78 | 6.55 | -726.49 | 50.00 | 340.42 |
| 39 | 23.77 | 42.28 | 9.63 | -634.52 | 30.00 | 16.09 |
| 40 | 15.80 | 2.45 | 5.28 | 174.84 | 7.60 | 350.84 |
| 41 | **678.46** | **833.33** | 48.88 | 7859.59 | 553.33 | 16.09 |
| 42 | **425.69** | 5.46 | 274.94 | 6822.20 | 109.20 | 1371.13 |
| 43 | **546.70** | 5.46 | 279.95 | 7643.89 | 388.00 | 1352.66 |

^a^ THF solvent, H_2_O as acid with 1000 eq, SmI_2_(THF)_2_ as reductant with 1000 eq, 25 h of reaction time.

# Transfer learning

| a)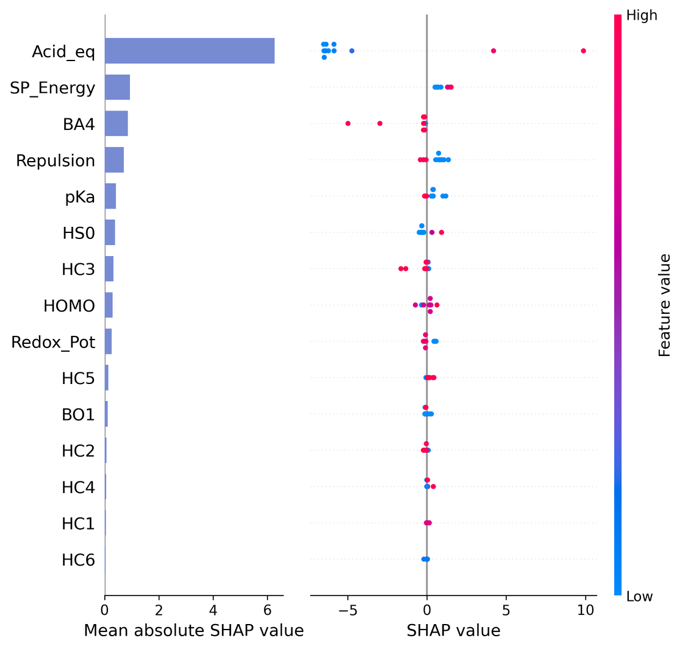 | b)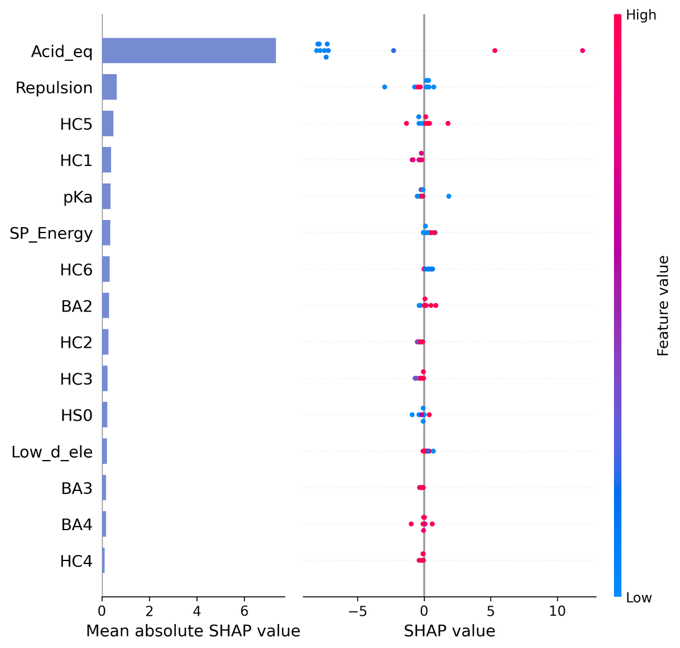 |
| --- | --- |

**Figure S30.** SHAP feature analysis for (a) TOF and (b) TON with family 1 ET and AB models over the external validation dataset (family 3 catalysts).

| 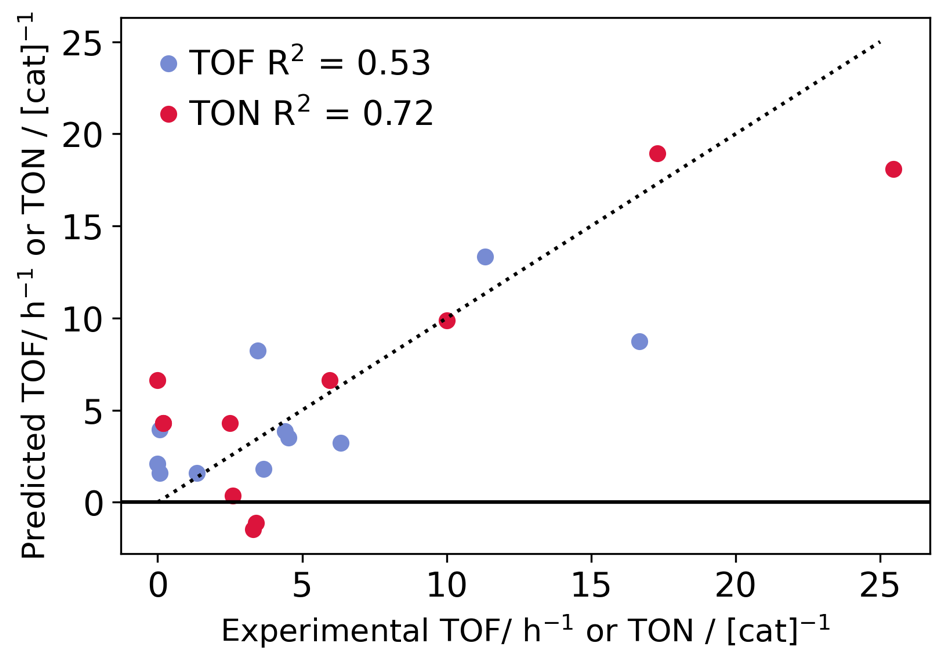 |
| --- |

**Figure S31.** TOF and TON prediction accuracy in transfer learning of MLP family 1 models, followed by a linear regression calibration, on an external dataset from family 3 catalysts.

# Overall workflow

The workflow can be run in three different forms, invoked by the ‘*-xyz*’, *‘-smi*’ and ‘*-write*’ command line flags. In the ‘*-write*’ mode, the input file information is used only to generate the molecular structure of the complex and to write its SMILES. On the other hand, the ‘*-xyz*’ and ‘*-smi*’ flags activate the full workflow. The only difference between the two lies in the first step: in the ‘*-smi*’ case, the XYZ coordinates are generated starting from the SMILES of the isolated ligands, while in the ‘*-xyz*’ mode, the XYZ coordinate file must be provided as input. Once the structure is defined, it undergoes a multilevel geometry optimization at UFF, GFN2-xTB, B97-3c and PBE0-D4/def2-SVP level of theory. All DFT calculations are carried out in ORCA with the CPCM implicit solvation model. The optimization is followed by a final single-point calculation at PBE0-D4/def-TZVPD level. Then, other possible spin multiplicities are generated according to crystal field theory, based on the metal formal oxidation state. For each spin state, an additional geometry optimization at PBE0-D4/def2-SVP and a corresponding single-point calculation are performed. Finally, the lowest spin state is identified, and a set of molecular descriptors is parsed. The extracted descriptors are then used to predict the catalytic activity parameters using the trained ML classification and regression models.

**Structure generation**. The *generation.py* script allows the creation of coordination compounds based on the most common topologies of proposed NRR catalysts. In particular, we developed a customized version of the stk library by inclusion of new topologies and types of ligands, compared to the original one. The XYZ structure of a potential catalyst is generated from a simple input file specifying the following: structure name, metal atom, solvent name, total molecular charge, metal formal oxidation state, SMILES of each ligand (with appropriate dummy atoms) and experimental conditions to use in the ML prediction. The *generation.py* script parses this information and transfers it to the implemented Generator class, which has a function for each topology. Taking the OBD case as an example process starts by defining the stk building blocks for the metal center, bridging dinitrogen and the additional dinitrogen ligand. Then, the function divides based on the type of ligand in PNP, PCP and 5PNP. In the PNP case, the tridentate ligand building block is obtained with the SMILES string parsed from the input file and the binding sites are identified by the presence of dummy atoms in the SMILES. The complex structure is generated by placing the building blocks according to the predefined topology and connecting the binding sites to the metal centre. Then, the generated structure is minimized at the UFF level in pybel with a maximum of 10000 steps. The final geometry is printed as an XYZ coordinate file, and a summary of the information needed to run the following quantum chemical calculations is printed at the end of the input file. Additionally, *generator.py* outputs the full catalyst SMILES.

**Structure description**. Quantum chemical calculations are handled by four modules, written in either bash or Python. The first step is the *calculate.sh*, which carries out a multilevel geometry optimization and a final single point calculation in the lowest possible spin multiplicity. The initial geometry optimization is done at the semiempirical GFN2-xTB level, followed by a frequency calculation at the same level of theory. If the calculation converges and the structure is a minimum, the workflow proceeds; otherwise, it attempts a second GFN2-xTB optimization starting from a structure distorted along the imaginary mode vector. Then, the structure is optimized at a B97-3c level in ORCA, with the CPCM solvation model and 32 cores parallelization. The input files for all quantum chemical calculations are generated automatically by filling a predefined template with the input file information. Any error detected in the quantum chemical calculation leads to the exit from the whole workflow to prevent propagation of incorrect structures. The final optimization step is done at the PBE0-D4/def2-SVP level with CPCM implicit solvation in ORCA, followed by a final single-point calculation at the PBE0-D4/def2-TZVPD level. The next step is assessing other possible spin multiplicities for the complex, handled by *spin.py*. Based on the metal atom and its oxidation state from the initial input file, it calculates the possible spin states according to crystal field theory. If multiple metal atoms are present, the possible multiplicities are generated for each atom, and all the possible combinations are computed. The resulting multiplicities are given as output and passed to *higher_state.sh* to run additional quantum chemical calculations. In particular, for each multiplicity, a geometry optimization at PBE0-D4/def2-SVP level is carried out from the lowest multiplicity optimized structure, and a final single point is done at PBE0-D4/def2-TZVPD level.

**Data analysis***.* The results obtained by the single-point calculations on different multiplicities are parsed by *analysis.py*, which identifies the most stable multiplicity state. Then, *extract_data.py* parses the output file and extracts several molecular descriptors. The functions needed for the data extraction are bundled in the *ExtractData* class. Mulliken and Hirshfeld analysis, bond orders, bond angles and bond distances data are selected only for the six closest atoms to each metal centre, which are obtained by a specifically designed function. The HOMO-LUMO gap and the Mulliken analysis functions have two different forms, corresponding to unrestricted (UHF, spin multiplicity > 1) or restricted (RHF, spin multiplicity = 1) calculations. In the UHF approach, the HOMO-LUMO gap is computed as the smallest energy difference between the highest occupied and lowest unoccupied orbitals, without considering if they have the same or opposite spin state (α or β). For the Mulliken analysis, the difference is that only the UHF calculation presents spin population data. The buried volume is computed by the DBSTEP library, while other functions parse molecular descriptors such as dipole moment and repulsion energy. At the end, the data extracted is printed to a formatted text file.

**ML prediction**. Finally, the file with the extracted data is parsed by the *DataPrep* class in the *ML_predict.py* script, and the data are arranged in an ordered dataframe. Then, depending on the complex family, the data are passed to functions in the *ML_prediction* class, which uses the trained regressor and classification ML models to predict the catalytic activity parameters. The results are printed both in the log file of the workflow and in a specific text file.

In the *ML_prediction* class, the input data from DFT calculations are randomly divided into a training set and a testing set, with a ratio of 8:2. To demonstrate the stability and accuracy of the ML model, a 10-fold CV was applied to all algorithms to find the optimal combination of hyperparameters for all models. The hyperparameters of each algorithmic model were optimized by grid search with nested CV in order to prevent training overfitting. The SMOTE method was used for data enhancement, considering the imbalance of the data samples for the classification models. The accuracy evaluation metrics for the ML models include four measures: R^2^, MAE, MSE, and RMSE. Shapley Additive Explanations (SHAP) analysis was conducted on the model. SHAP stands out as one of the widely accepted methods for elucidating ML models. In this approach, each feature is assigned an importance scale, where a higher absolute SHAP value denotes a more substantial contribution to the outcomes of ML models. Moreover, a positive or negative SHAP value signifies that the feature exerts a positive or negative effect on the prediction. Additionally, partial dependence plots (PDP) have been calculated for the regression models. The PDP represents the relationship between the target and a feature value, allowing for complementing the SHAP analysis by identifying if this relationship is linear, monotonic or a more complex function. Thus, it can provide a quantitative estimation of the optimal range of a feature.

The entire workflow has been tested on the supercomputer with 32 cores with high efficiency and smoothly. The workflow implemented with the external software, includ-ing ORCA, xTB, and several Python modules: stk (and mods.py), RDKit, pybel, scipy, numpy, pandas, itertools, scikit-learn (v. 1.6.1), dbstep, pickle, etc. Among them, xTB and ORCA are used to carry out quantum-chemical calculations. The ML scripts and their analysis use scikit-learn (v. 1.6.1), xgboost and shap libraries; the models are exported and loaded in the final workflow step by pickle.

# References

(1) Arashiba, K.; Miyake, Y.; Nishibayashi, Y. A Molybdenum Complex Bearing PNP-Type Pincer Ligands Leads to the Catalytic Reduction of Dinitrogen into Ammonia. *Nature Chem* **2011**, *3* (2), 120–125. https://doi.org/10.1038/nchem.906.

(2) Kuriyama, S.; Arashiba, K.; Tanaka, H.; Matsuo, Y.; Nakajima, K.; Yoshizawa, K.; Nishibayashi, Y. Direct Transformation of Molecular Dinitrogen into Ammonia Catalyzed by Cobalt Dinitrogen Complexes Bearing Anionic PNP Pincer Ligands. *Angewandte Chemie International Edition* **2016**, *55* (46), 14291–14295. https://doi.org/10.1002/anie.201606090.

(3) Beasley, C. H.; Duletski, O. L.; Stankevich, K. S.; Arulsamy, N.; Mock, M. T. Catalytic Dinitrogen Reduction to Hydrazine and Ammonia Using Cr(N_2_ )_2_ (Diphosphine)_2_ Complexes. *Dalton Trans.* **2024**, *53* (15), 6496–6500. https://doi.org/10.1039/D4DT00702F.

(4) Yandulov, D. V.; Schrock, R. R. Reduction of Dinitrogen to Ammonia at a Well-Protected Reaction Site in a Molybdenum Triamidoamine Complex. *J. Am. Chem. Soc.* **2002**, *124* (22), 6252–6253. https://doi.org/10.1021/ja020186x.

(5) Anderson, J. S.; Rittle, J.; Peters, J. C. Catalytic Conversion of Nitrogen to Ammonia by an Iron Model Complex. *Nature* **2013**, *501* (7465), 84–87. https://doi.org/10.1038/nature12435.

(6) Adamo, C.; Barone, V. Toward Reliable Density Functional Methods without Adjustable Parameters: The PBE0 Model. *The Journal of Chemical Physics* **1999**, *110* (13), 6158–6170. https://doi.org/10.1063/1.478522.

(7) Becke, A. D. Density-Functional Thermochemistry. III. The Role of Exact Exchange. *The Journal of Chemical Physics* **1993**, *98* (7), 5648–5652. https://doi.org/10.1063/1.464913.

(8) Lee, C.; Yang, W.; Parr, R. G. Development of the Colle-Salvetti Correlation-Energy Formula into a Functional of the Electron Density. *Phys. Rev. B* **1988**, *37* (2), 785–789. https://doi.org/10.1103/PhysRevB.37.785.

(9) Zhao, Y.; Truhlar, D. G. The M06 Suite of Density Functionals for Main Group Thermochemistry, Thermochemical Kinetics, Noncovalent Interactions, Excited States, and Transition Elements: Two New Functionals and Systematic Testing of Four M06-Class Functionals and 12 Other Functionals. *Theor Chem Account* **2008**, *120* (1–3), 215–241. https://doi.org/10.1007/s00214-007-0310-x.

(10) Perdew, J. P.; Tao, J.; Staroverov, V. N.; Scuseria, G. E. Meta-Generalized Gradient Approximation: Explanation of a Realistic Nonempirical Density Functional. *The Journal of Chemical Physics* **2004**, *120* (15), 6898–6911. https://doi.org/10.1063/1.1665298.

(11) Chai, J.-D.; Head-Gordon, M. Long-Range Corrected Hybrid Density Functionals with Damped Atom–Atom Dispersion Corrections. *Phys. Chem. Chem. Phys.* **2008**, *10* (44), 6615–6620. https://doi.org/10.1039/B810189B.

(12) Brandenburg, J. G.; Bannwarth, C.; Hansen, A.; Grimme, S. B97-3c: A Revised Low-Cost Variant of the B97-D Density Functional Method. *The Journal of Chemical Physics* **2018**, *148* (6), 064104. https://doi.org/10.1063/1.5012601.

(13) Grimme, S.; Hansen, A.; Ehlert, S.; Mewes, J.-M. r2SCAN-3c: A “Swiss Army Knife” Composite Electronic-Structure Method. *The Journal of Chemical Physics* **2021**, *154* (6), 064103. https://doi.org/10.1063/5.0040021.

(14) Schrödinger, LLC. The PyMOL Molecular Graphics System, Version 1.8, 2015.

(15) Rappe, A. K.; Casewit, C. J.; Colwell, K. S.; Goddard, W. A.; Skiff, W. M. UFF, a Full Periodic Table Force Field for Molecular Mechanics and Molecular Dynamics Simulations. *J. Am. Chem. Soc.* **1992**, *114* (25), 10024–10035. https://doi.org/10.1021/ja00051a040.

(16) O’Boyle, N. M.; Banck, M.; James, C. A.; Morley, C.; Vandermeersch, T.; Hutchison, G. R. Open Babel: An Open Chemical Toolbox. *Journal of Cheminformatics* **2011**, *3* (1), 33. https://doi.org/10.1186/1758-2946-3-33.

(17) Bannwarth, C.; Ehlert, S.; Grimme, S. GFN2-xTB—An Accurate and Broadly Parametrized Self-Consistent Tight-Binding Quantum Chemical Method with Multipole Electrostatics and Density-Dependent Dispersion Contributions. *J. Chem. Theory Comput.* **2019**, *15* (3), 1652–1671. https://doi.org/10.1021/acs.jctc.8b01176.

(18) Bannwarth, C.; Caldeweyher, E.; Ehlert, S.; Hansen, A.; Pracht, P.; Seibert, J.; Spicher, S.; Grimme, S. Extended tight‐binding Quantum Chemistry Methods. *WIREs Comput Mol Sci* **2021**, *11* (2), e1493. https://doi.org/10.1002/wcms.1493.

(19) Neese, F.; Wennmohs, F.; Becker, U.; Riplinger, C. The ORCA Quantum Chemistry Program Package. *The Journal of Chemical Physics* **2020**, *152* (22), 224108. https://doi.org/10.1063/5.0004608.

(20) Neese, F. Software Update: The ORCA Program System—Version 6.0. *WIREs Computational Molecular Science* **2025**, *15* (2), e70019. https://doi.org/10.1002/wcms.70019.

(21) Namazian, M.; Coote, M. L. Accurate Calculation of Absolute One-Electron Redox Potentials of Some *Para* -Quinone Derivatives in Acetonitrile. *J. Phys. Chem. A* **2007**, *111* (30), 7227–7232. https://doi.org/10.1021/jp0725883.

(22) Namazian, M.; Lin, C. Y.; Coote, M. L. Benchmark Calculations of Absolute Reduction Potential of Ferricinium/Ferrocene Couple in Nonaqueous Solutions. *J. Chem. Theory Comput.* **2010**, *6* (9), 2721–2725. https://doi.org/10.1021/ct1003252.

(23) Itabashi, T.; Mori, I.; Arashiba, K.; Eizawa, A.; Nakajima, K.; Nishibayashi, Y. Effect of Substituents on Molybdenum Triiodide Complexes Bearing PNP-Type Pincer Ligands toward Catalytic Nitrogen Fixation. *Dalton Trans.* **2019**, *48* (10), 3182–3186. https://doi.org/10.1039/C8DT04975K.

(24) Kuriyama, S.; Arashiba, K.; Nakajima, K.; Matsuo, Y.; Tanaka, H.; Ishii, K.; Yoshizawa, K.; Nishibayashi, Y. Catalytic Transformation of Dinitrogen into Ammonia and Hydrazine by Iron-Dinitrogen Complexes Bearing Pincer Ligand. *Nat Commun* **2016**, *7* (1), 12181. https://doi.org/10.1038/ncomms12181.

(25) Arashiba, K.; Eizawa, A.; Tanaka, H.; Nakajima, K.; Yoshizawa, K.; Nishibayashi, Y. Catalytic Nitrogen Fixation via Direct Cleavage of Nitrogen–Nitrogen Triple Bond of Molecular Dinitrogen under Ambient Reaction Conditions. *Bulletin of the Chemical Society of Japan* **2017**, *90* (10), 1111–1118. https://doi.org/10.1246/bcsj.20170197.

(26) Arashiba, K.; Kinoshita, E.; Kuriyama, S.; Eizawa, A.; Nakajima, K.; Tanaka, H.; Yoshizawa, K.; Nishibayashi, Y. Catalytic Reduction of Dinitrogen to Ammonia by Use of Molybdenum–Nitride Complexes Bearing a Tridentate Triphosphine as Catalysts. *J. Am. Chem. Soc.* **2015**, *137* (17), 5666–5669. https://doi.org/10.1021/jacs.5b02579.

(27) Lundberg, S. M.; Erion, G.; Chen, H.; DeGrave, A.; Prutkin, J. M.; Nair, B.; Katz, R.; Himmelfarb, J.; Bansal, N.; Lee, S.-I. From Local Explanations to Global Understanding with Explainable AI for Trees. *Nat Mach Intell* **2020**, *2* (1), 56–67. https://doi.org/10.1038/s42256-019-0138-9.

(28) Yamazaki, Y.; Nakaya, K.; Ishitobi, Y.; Nakamura, T.; Egi, A.; Tanaka, H.; Yoshizawa, K.; Nishibayashi, Y. Highly Efficient Molybdenum-Catalyzed Catalytic Ammonia Pro-Duction: Synergistic Effect of Electron-Withdrawing and Extension of π-Conjugation. Chemistry November 21, 2025. https://doi.org/10.26434/chemrxiv-2025-klrt8.
